# What keeps gig workers motivated? A typology of the intrinsic-extrinsic motivation interplay in the digital gig economy

**DOI:** 10.1371/journal.pone.0345881

**Published:** 2026-09-22

**Authors:** Yuthara Subasinghe, Jameel Shuhaib, Senuri Kelly, Rashini Maleesha, Krishantha Wisenthige, Lochana Perera

**Affiliations:** 1 Department of Business Management, Sri Lanka Institute of Information Technology, Malabe, Sri Lanka; 2 Department of Information Management, Sri Lanka Institute of Information Technology, Malabe, Sri Lanka; Indira School of Business Studies, INDIA

## Abstract

The rise of digital platforms has intensified scholarly interest in understanding how gig workers remain motivated. This paper explores the influence of digital skills in shaping gig worker motivation and engagement, with particular focus given to the dynamic interplay between intrinsic and extrinsic motivation. A qualitative research design was employed, utilising snowball sampling to garner insights from platform-based gig workers in Sri Lanka. A total of 14 semi-structured interviews were conducted with gig workers primarily engaging in Upwork and Fiverr, with the results being analysed using thematic analysis. The findings reveal that digital skills function beyond technical competencies by being psychological enablers of autonomy, competence, and relatedness. Similarly, intrinsic and extrinsic motivational drivers can interact in a reinforcing or controlling manner creating distinct motivation patterns that shape engagement. Platform providers can utilise these insights to design reinforcement oriented motivational features such as informational feedback systems and transparent performance metrics. The motivation typology offers guidance in developing engagement strategies tailored to different motivational profiles, which will foster economic growth aligned with SDG 8, ensuring decent employment opportunities. Limitations include the focus on the Sri Lankan context which limits the findings to South Asia while restricting broader generalisability. Future research could benefit from employing representative samples across various cultural contexts. This study contributes to the limited empirical work surrounding the psychological mechanisms linking digital skills and gig worker engagement. It offers new insights through the introduction of a motivation interplay matrix and typology which provides a framework for understanding gig worker engagement.

## 1. Introduction

The gig economy has a layered history, with the term “gig” originating from jazz performers in 1915. The business model of providing temporary labour to corporations first took hold in the 1940’s to address workforce shortages of World War II [1]. Over the decades, this model has evolved from a niche labour solution to a dominant global force, fundamentally reshaping the nature of work [2,3]. Gig work is defined as a temporary, project-based employment characterized by work flexibility, task-specific compensation, and non-membership in a traditional organization, primarily facilitated through digital platforms [4]. This emerging model of work emphasizes short-term, platform-driven contracts, reflecting a profound move away from the stability of traditional employment [5]. Technological advancements and digital platforms have accelerated the rapid expansion of the gig economy, transforming it from a niche sector into a major segment of the global labour market, offering unprecedented flexibility and accessibility for workers worldwide [6,7]. According to recent estimates by the World Bank [8], states that the gig economy represents approximately 12% of the global labour force, highlighting its growing prominence as a substantial employment sector worldwide. The global estimates underscore its scale as this trend was significantly accelerated by the COVID-19 pandemic’s normalisation of remote work [9]. Gig work is broadly divided into platform-based and location-based types. Platform-based gig work refers to digitally mediated employment carried out remotely via online platforms such as Upwork or Fiverr. The most commonly undertaken gigs in platform-based gig work ranges from graphic design, web development, content writing, and project-based gig work [7]. Location-based gig work, by contrast, involves tasks that require the gig worker to be physically present in a certain place [9]. Accordingly, the scope of the present study is limited to platform-based gig workers due to the growing prominence of digitally mediated freelance work in the modern labour market [10]. In the context of Sri Lanka, the digital gig economy presents a vital opportunity for income generation, employment diversification, and leveraging of the country’s increasingly digitally literate youth population [11]. Successful and sustained engagement in this highly competitive globalised field can depend upon numerous factors. A critical factor that potentially determines a worker’s ability to participate, compete, and thrive is the digital skills they possess [12,13]. [13] broadly characterizes digital skills as understanding the potential of digital technologies and having the capability to interact with them in an efficient and effective manner. This elaborates that digital skills refer to broader competencies beyond the ability to use a computer. Consequently [14], and [15], suggests that digital skills play a significant role in shaping gig economy participation, where digitally proficient individuals unveil a greater tendency to undertake gig work. However, although there is a direct relationship between digital skills and engagement in the gig economy, this relationship can be affected by critical psychological and behavioural factors. Within the gig economy, motivation has been identified as a critical factor influencing worker commitment, performance quality, and continued platform engagement [16]. Despite these theoretical propositions, research has not yet deeply examined how gig workers themselves perceive and experience the relationship between digital skills, motivation, and gig worker engagement. Existing literature reveals critical limitations that examine intrinsic and extrinsic motivation in isolation, with limited exploration into how these motivational forces interact and coexist to sustain engagement. Recent investigations conducted by [17], examines the relationship between specific variables such as job autonomy, remuneration, social connection, and technology, yet overlook the role of technical competencies in influencing motivation and engagement. Moreover [16], also highlights the need of scholarly inquiry into the psychological aspect and motivational structures of gig work, providing a foundation for building more engaging and fair work environments. Critically, quantitative methodologies have been acknowledged as insufficient for capturing diverse, nuanced perspectives of gig workers regarding how digital skills shape motivational experiences [18]. This collection of limitations reveals a critical gap: prior research has not adequately explored how digital skills may shape gig worker motivation and engagement in the digital gig economy. This study aims to address this gap by focusing on the following interrelated research questions:

(1) How do gig workers describe the role of digital skills in shaping motivational experiences in digital gig platforms?
(2) How does intrinsic and extrinsic motivation interact within the digital gig economy to sustain gig worker engagement?
(3) What motivation patterns emerge from the interplay between intrinsic and extrinsic motivation in the digital gig economy?

By investigating the complex interplay between intrinsic and extrinsic motivation within the digital gig economy, this research seeks to provide a comprehensive empirical understanding of how these motivational dimensions influence digital skills and engagement among Sri Lankan gig workers. Understanding this dynamic is critical for designing policies and platforms that sustain gig worker motivation, productivity, and well-being in an evolving labour market increasingly characterized by flexibility.

## 2. Review of existing literature

This section presents a critical analysis and interpretation of past literature surrounding digital skills, extrinsic motivation, and intrinsic motivation. To develop a strong theoretical foundation, scholarly articles were identified through academic databases such as ScienceDirect, Google Scholar, and Emerald. The main search terms included variations of “Digital skills”, “Intrinsic Motivation”, “Engagement”, “Extrinsic motivation”, and “Digital Labour”.

### 2.1. Conceptualizing digital skills and its influence in the digital gig economy

Digital skills encompass a plethora of competencies that have gained significance in recent years [19]. Despite its widespread use in organizational and gig economy research, the term “digital skills” lacks a universal definition, creating ambiguity in terms of its interpretation [20]. The evolving nature of digital skills may contribute to this existing ambiguity by increasing the need to be redefined constantly [21]. Scholars have discussed the concept of digital skills interchangeably through various terms since the early stages of technological advancements. Early literature on digital skills utilizes terms such as “Digital literacy” [22], to highlight the various skills and competencies that reflect the practical needs required to operate in a digitally transformative world. Similarly, recent studies incorporate terms such as “twenty-first-century digital skills” [23] and “twenty-first-century competencies” [24] to highlight numerous skills that encompass the broader concept of digital skills, rendering it as multifaced in nature. It is notable that mastering a digital skill set creates positive outcomes such as improvements in quality of life, higher career opportunities, and earnings [25] as digital skills serve as a crucial requirement for employability [12].

Drawing on this significance, it is crucial to examine the concept of digital skills in the context of the digital gig economy as it can significantly shape the way gig workers engage with digital platforms. Possessing a strong digital skill set enables a gig worker to effectively utilize platform tools in their favor which ultimately builds self-reliance and resilience when encountered with hardships [13]. These attributes are deemed crucial to combat the precarious nature of digital platforms and to remain competitive in the industry [14]. expands these findings by highlighting that digital platform precarity arises due to invisible labour such as the growing need for additional skills rather than an unstable job market. The study stresses the importance of developing a plethora of digital competencies in order to remain competitive and sustain engagement in the gig economy. This perspective aligns with [14] where the study finds that digitally savvy individuals may thrive in the digital gig economy in comparison to gig workers who may not possess the same level of skills [26]. evidence strengthens this broader view by showing that digital competence is not limited to basic technical ability but reflects a multidimensional capability needed to function effectively in digital environments. In particular, in this study digital transformation skills identifies skillsets such as digital work, communication, collaboration, adaptation, and evidence-based work as core components of digital competence, which supports the argument that gig workers need more than operational platform knowledge to succeed in digitally mediated work.

### 2.2. Conceptualising intrinsic motivation in the digital gig economy

Early research on motivation stems from Self Determination Theory (SDT) proposed by [27], whereby motivation is believed to manifest into two distinct fundamental forms: Intrinsic and extrinsic. Intrinsic motivation is defined as an autonomous motivation whereby individuals engage in activities out of their own choice, freewill, and volition [28]. It encompasses an individual’s inherent interest and satisfaction in completing a task, rather than a result of mere obligation. According to the principles of SDT theory, intrinsic motivation arises when the universal psychological need for autonomy, competence, and relatedness are fulfilled [28]. In the digital gig economy, intrinsic motivation typically emerges from the perceived flexibility and autonomous nature of digital gig work [16], alongside platform tools which allow for skill recognition and praise [29].

Drawing on this significance, it is essential to critically examine the mechanisms that drive intrinsic motivation to expand the understanding of its impact in the digital gig economy. Empirical studies have consistently proven that perceived autonomy as a job characteristic has a positive influence on a worker’s emotions and intrinsic motivation [30]. However, perceptions of autonomy in the digital gig economy may differ from traditional workplaces due to distinctions in the overall working environment [31]. According to the findings of [32], gig workers that engage in gig work out of inherent enjoyment, often referred to as “Dabblers”, predominantly exhibit intrinsic motivation driven by the ability to choose when they prefer to work. Despite the independence offered in gig work [3], argues that autonomy can be constrained by algorithmic management mechanisms which determine a gig worker’s access to gigs and income. The findings reveal that in such circumstances autonomy can be both empowering and a source of frustration. Thereby, the study brings new insights about the contingent nature of autonomy as it tends to be influenced by the perceived authenticity of autonomy.

Another significant component that drives intrinsic motivation is a worker’s perceived competence [27]. In the gig economy, competence may translate to positive and affirmative feedback provided by clients which allow gig workers to feel validated as their skills are recognized [33]. Due to the competitive nature of the digital gig economy, digital skills serve as a critical requirement to succeed and maintain engagement [13]. Therefore [34], elucidates that gig workers must dedicate time to consistently develop new skills and competencies as self-determination to consistently improve will drive intrinsic motivation. Furthermore, the need for relatedness serves as an equally essential component as feelings of belonging to a group contribute to achieving intrinsic motivation. Due to the nature of gig work being remote or platform based [33], suggest that feelings of relatedness may arise from client feedback or positive client interactions as it will enable gig workers to recognize their own value which ultimately develops intrinsic motivation [35].

### 2.3. Conceptualising extrinsic motivation in the digital gig economy

Early research on extrinsic motivation defines it as a construct that pertains when an activity is done in an attempt to achieve a separable outcome [36]. This could include instrumental reasons such as tangible rewards, recognition, and monetary gains [35]. In the context of the gig economy, the concept of extrinsic motivation is particularly salient as it involves monetary rewards, customer ratings, and performance-based incentives, which play a critical role in the choice to enter and engage in the gig economy [37]. According to [38], digital platforms must design compensation systems that accommodate the fact that gig workers are primarily motivated by financial rewards, as remuneration is a key driver of motivation [17]. Recent empirical evidence also suggests that payment design in gig work does not operate in isolation, because workers’ responses to incentives are shaped by their intrinsic task motivation and perceptions of control. In a field experiment on online freelancers [39], found that a payment scheme combining commission with a fixed component reduced performance among less intrinsically motivated workers but improved performance among highly intrinsically motivated workers, showing that extrinsic incentives can have different effects depending on worker motivation profiles. However [40], argues that leveraging extrinsic rewards within the workplace can encourage employees to act opportunistically and neglect aspects of their job that is not monetized [16]. contributes to this discourse with its application in the digital gig economy, whereby their findings reveal that leveraging intrinsic motivation is more beneficial for platforms.

Moreover, positive gestures such as praise and recognition are also deemed to cultivate extrinsic motivation [41]. [42] expand on these findings by revealing that higher management and supervisors must praise the employees that report to them as it critically contributes to their extrinsic motivation. In the digital gig economy, praise and recognition may arise from gamification such as badges, leaderboards, and feedback systems [29]. Gamified recognition should also be interpreted cautiously, because feedback systems may function as both motivational support and behavioural control in digital labour settings. When platform feedback is tied to visibility, ranking, or access to future work, recognition can reinforce engagement while simultaneously increasing pressure to conform to platform expectations. This makes praise and recognition a potentially mixed extrinsic driver rather than a uniformly positive one [43]. Although praise and recognition are deemed to generate extrinsic motivation, interestingly it may overlap with intrinsic motivation as it fulfils the psychological need for competence [28]. This duality supports the idea that individuals can experience intrinsic and extrinsic motivation simultaneously [35], whereby extrinsic incentives can support intrinsic motivation when they are internalized [37]. Therefore, the interplay between intrinsic and extrinsic motivation in the digital gig economy may provide valuable insights into how motivational drivers interact with digital skills to shape gig worker engagement.

### 2.4. Conceptualising engagement within digital gig work

Engagement is a multidimensional construct that has been conceptualised through behavioural, cognitive, and emotional dimensions, reflecting the extent to which individuals invest their effort, attention, and psychological resources in work-related activities [44,45]. However, in digitally mediated labour markets, engagement extends beyond psychological attachment and is increasingly reflected in workers’ continued participation within platforms. Prior research suggests that the sustainability of digital labour platforms depends not merely on workers’ temporary involvement but on their continuous and uninterrupted participation over time [29]. Similarly, gig economy scholars argue that engagement is reflected through workers’ willingness to remain active, repeatedly accept work opportunities, and sustain their involvement despite the inherent uncertainties of platform-based employment [3,43]. Drawing on this perspective, the present study conceptualises engagement as continuance engagement, defined as the extent to which gig workers sustain their participation in digital labour platforms through continued acceptance of projects, ongoing involvement in gig activities, and persistence in platform-based work overtime. This conceptualisation is particularly appropriate given that participation in gig work is largely voluntary and influenced by workers’ motivational experiences. Therefore, while factors such as creative autonomy, work commitment, client relationships, and satisfaction with platform support emerged during the interviews, these are interpreted as antecedents that facilitate engagement rather than engagement itself. Accordingly, this study focuses specifically on the behavioural outcome of sustained platform participation rather than emotional attachment, cognitive absorption, job satisfaction, or organisational commitment, thereby examining how intrinsic and extrinsic motivational forces contribute to the continued participation of gig workers within the digital gig economy.

## 3. Materials and methods

This study utilises a qualitative method by developing thematic maps for each variable in order to derive meaningful insights. A qualitative basis was chosen as it enables researchers to investigate and seek answers through social experiences [46]. The interview series enabled participants to express their experiences through the usage of open-ended questions, giving them the freedom to voice their opinions surrounding their participation in the digital gig economy.

### 3.1. Population and sample

The population considered were full-time and part-time digital gig workers in Sri Lanka above the age of 18. The participants were recruited using snowball sampling whereby initial participants who met the inclusion criteria later recommended the subsequent participants [47]. This sampling technique is methodologically appropriate in situations where the target population is difficult to reach through conventional sampling techniques [48]. The absence of a standard count of digital gig workers in Sri Lanka led to the application of a non-probability approach enabling the researcher to offer credible and meaningful results [49]. These participants had to be currently engaged in online freelancing platforms. The sample size was deemed adequate upon reaching code saturation during the interview process

### 3.2. Data collection method

Primary data was collected through 14 in-depth semi-structured interviews with open-ended questions spanning the course of two weeks, with an average session being approximately 28.4 minutes. The interviews were conducted by two researchers at a time and participants were asked broad open-ended questions that focused on skill acquisition, factors that motivated them to engage in gig work, the influence of digital skills on participation in gig work, and challenges incurred while working on digital labour platforms. Through the interview series, the researchers discovered participants’ perceptions and experiences working in online freelancing platforms. During this stage, interviews were continuously reviewed to identify the emergence of new codes and themes. Interview process was concluded when code saturation was reached approximately at the 12^th^ interview as code generation progressively declined. Table 1 illustrates the saturation table, whereby participant 12 generates only one new code (2.22%) indicating near saturation and aligning with [50] ≤5% threshold. Hence, interviews 13 and 14 were conducted to primarily strengthen existing themes and contributed to thematic robustness [50]. All participants were assigned pseudonyms to safeguard their privacy. Interviews were conducted online through Microsoft Teams with permission granted from participants to record each session. Transcripts were generated using the tool Premiere Pro from Adobe Creative Suite which were further spot-checked by the authors for accuracy and clarification, to ensure transcript verification [51]. Table 2 provides an overview of the participants’ demographics and characteristics.

**Table 1:** Saturation Table. (Source: Authors’ composition).

Interview	Participant	New Codes	Cumulative Total	New codes as %	Cumulative % of code set
1	P1	5	5	11.11%	11.11%
2	P2	6	11	13.33%	24.44%
3	P3	4	15	8.89%	33.33%
4	P4	3	18	6.67%	40.00%
5	P5	6	24	13.33%	53.33%
6	P6	4	28	8.89%	62.22%
7	P7	4	32	8.89%	71.11%
8	P8	2	34	4.44%	75.56%
9	P9	2	36	4.44%	80.00%
10	P10	2	38	4.44%	84.44%
11	P11	2	40	4.44%	88.89%
12	P12	1	41	2.22%	91.11%
13	P13	2	43	4.44%	95.56%
14	P14	2	45	4.44%	100.00%

**Table 2:** Interviewee Table. (Source: Authors’ composition).

Participation	Age	Gender	Field	Experience (Years)	Freelance preference	Interview Length
P1	24	Male	Digital Art	5.5	Part-time	32 min
P2	23	Male	Digital Art	6	Part-time	23 min
P3	24	Male	Graphic Design	1	Part-time	39 min
P4	23	Male	Interior Design	1	Full-time	26 min
P5	21	Male	Character Art	0.5	Full-time	32 min
P6	24	Male	Video Editing	2	Part-time	41 min
P7	23	Male	Web Development	2	Part-time	27 min
P8	24	Female	Social Media Management	2	Full-time	33 min
P9	24	Male	Photo Editing	2	Part-time	23 min
P10	25	Male	Graphic/UI-UX/Photography	3	Full-time	19 min
P11	25	Male	Web Development	2.5	Part-time	29 min
P12	24	Male	Graphic/Web Development	3	Part-time	22 min
P13	24	Male	Social Media Handling	2	Part-time	20 min
P14	21	Male	Software Development	2	Part-time	21 min

### 3.3. Participants

A total of 14 digital gig workers representing diverse professional backgrounds were included in this study. In terms of freelancing field, majority of the participants belonged to creative multimedia work (n = 6) and software development (n = 5), while others engaged in professional services(n = 1) and sales and marketing (n = 2). Participants showcased varying levels of freelancing experience with majority reporting 1–5 years of experience (n = 11), while others had more than 5 years of experience (n = 2) and one participant having less than 1 year of experience (n = 1). Regarding working preference, majority of the participants engaged in digital gig work part time (n = 10), with a smaller portion preferring full time engagement (n = 4). In terms of platform preference, a significant portion of participants primarily used Fiverr (n = 9), with one participant using only Upwork, and a total of four participants using both Upwork and Fiverr.

### 3.4. Data analysis

**Fig 1:**
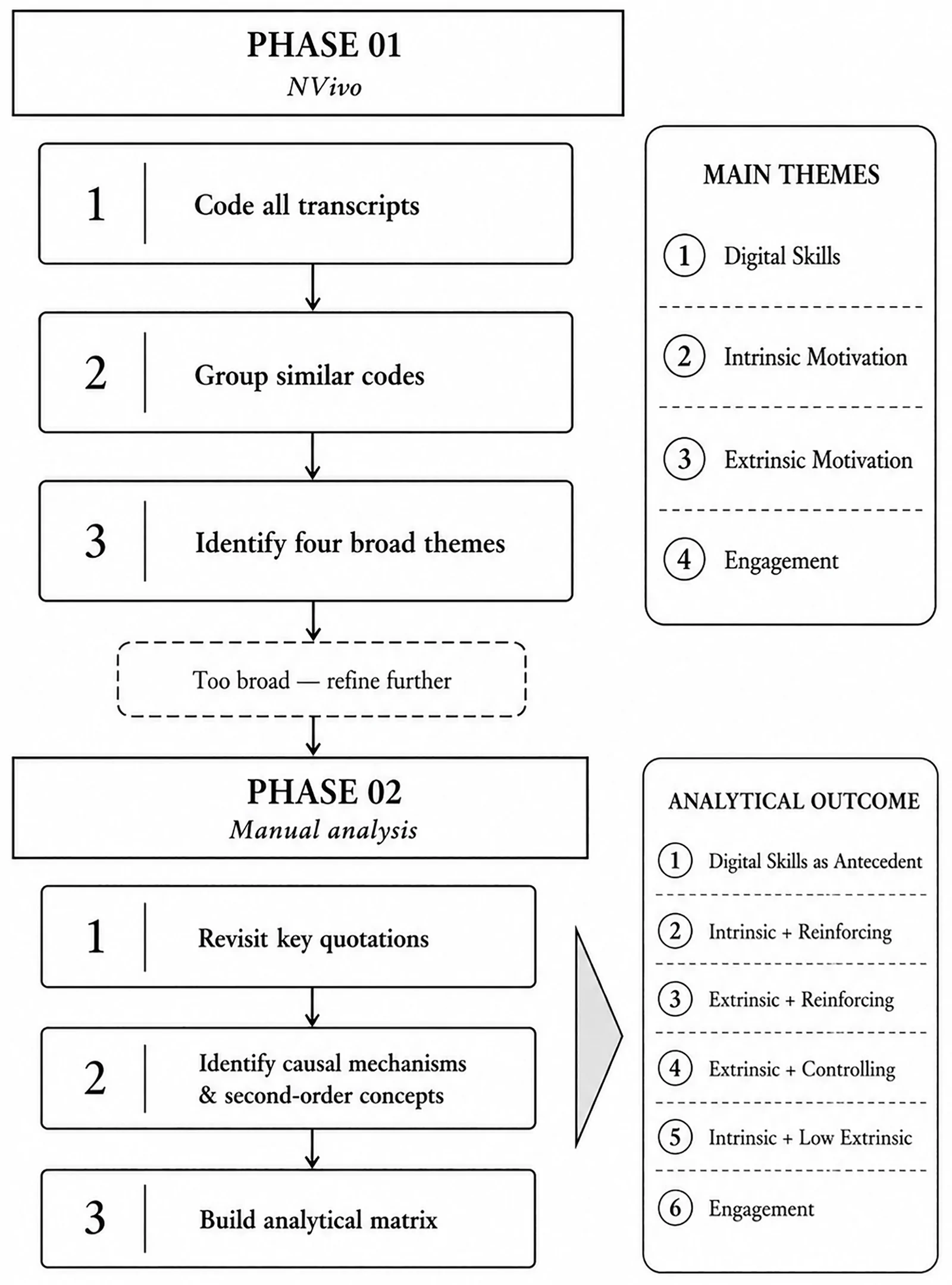
Data Analysis flow chart (Source: Authors’ creation).

Data analysis was conducted thematically [52] and was carried out in two complementary phases combining computer-assisted and manual techniques to strengthen the rigour and transparency of the process [53]. In the first phase, all interview transcripts were imported into NVivo and coded to identify recurring ideas and viewpoints illustrated by the participants. Through this, all authors worked simultaneously to ensure similar codes were grouped which formed four broad themes that captured the most prominent patterns across the dataset as supported S1 File. As these themes were relatively broad and did not fully capture the nuances within participant’s expressions, therefore, a second manual phase was commended using a spreadsheet by the main author. This enabled key quotes reflecting the primary themes to be organised and compared to surface more detailed patterns. This focused analysis refined the broad themes and led to the generation of first cycle codes which summarised the data alongside causation codes to causal relationships expressed by participants. Next, second cycle pattern codes were generated by grouping the first cycle codes into analytical labels that captured the relational configurations across participant accounts [54]. Hence, the causation codes developed served as the interaction codes through which relational patterns were observed between digital skills, motivation, and engagement as supported S2 File. This dual approach achieves the most robust results [53,55], with NVivo supporting efficient data management and manual analysis enabling deeper, more rigorous interpretation. Fig 1 illustrates the data analysis process with clarity.

### 3.5. Ethical considerations

This study rigorously adhered to established ethical standards surrounding primary research that incorporates human participants. An ethical approval was secured from Sri Lanka Institute of Information Technology Ethics Review Committee prior to data collection. The ethical approval reference no: SLIIT/ERC/SBS/2025/20 grants permission to conduct this study in all phases including participant recruitment and data analysis. In line with the institution’s ethics compliance process, all interview transcripts have been submitted to the Ethics Review Committee head, Professor Wasantha Rajapaksha. Hence, all transcripts have been deposited in the institutional repository. Additionally, all participants interviewed were thoroughly informed regarding details surrounding the study’s purpose, contribution, data usage, and the voluntary right to withdraw participation during any stage of the interview. Consent was obtained from all participants through a verbal reaffirmation of their rights prior to conducting each interview session whereby each verbal consent was recorded.

## 4. Results

### 4.1. The role of digital skills in shaping motivational experiences

Digital skills refer to a plethora of competencies that allow individuals to thrive in digital spaces [19]. A consistent pattern observed across participants was the role of digital skills in shaping motivational experiences, whereby participants who demonstrated a stronger skill set recall their motivation being driven by confidence and professional growth. Whereas participants who identified skill deficiencies emphasised the impact of these skill gaps in hindering motivational rewards. The most notable evidence of this pattern occurred as participants correlated their level of skills to their ability to engage in more gigs. Participant 5, a character artist, noted: *“I think I will be able to take up more kinds of gigs. I’m very into video editing and all that stuff, but I feel like I’m not pretty proficient in that. But I feel like if I was, I can”.* This excerpt captures how the degree of skill proficiency not only amplifies task performance but also impacts the psychological conditions that drive motivation. In such circumstances, gig workers who are unable to undertake desired gigs due to their limited skills are deprived of not only the potential income, but also the competence satisfaction arising from successful task completion. This will ultimately lead to a loss of both intrinsic and extrinsic motivational rewards simultaneously.

Conversely, participants that demonstrated stronger digital skills notably highlighted contrasting motivational experiences whereby skill mastery allows for higher efficiency when completing tasks. Participant 5 also noted: *“Alot of, like the shortcuts and stuff I use are my muscle memory. So like, it makes my work a lot faster because I don’t have to think, I can just go on”.* The excerpt demonstrates how expertise in digital tools can minimise cognitive load and encourage a faster output. In such instances, skill mastery emerges as a valuable tool that will free the gig worker’s attention to aspects such as creativity and quality which will ultimately contribute to intrinsic drive. Furthermore, participants also highlighted the significance of proactive skill development as a voluntary practice rather than an externally pressurised demand. Participant 14 espoused: *“I think it helps me to like, build up my, like, knowledge and technical skills, that I use, in like, day to day. It helps me a lot to do my work because I’m doing extra projects for different clients. So, it will be like a new experience for me”*. This excerpt emphasises how client-driven skill development emerges from the need to take on different clients for professional fulfilment rather than external pressure. Hence, the extrinsic drive to earn additional income through varied gigs is internalised through the intrinsic desire for continuous upskilling, aligning with the findings of [37] which demonstrates that extrinsic incentives support intrinsic motivation when internalised. Similarly, participant 8 reflects on the aspirational aspect of skill development by stating: *“Sometimes I feel like when it comes to video editing, I usually take like, a lot of time, and sometimes I wonder whether it is because I don’t know so much or something, but I feel like if I learn more, I feel like it will be easier and faster for me to do get stuff done”*. The excerpt captures how self-awareness of skill deficiencies generates the motivation to self-improve and develop stronger skills with the intention of enhancing efficiency. This self-directed practice can aid the gig worker to promptly finish tasks allowing them the liberty to take on more gigs. This sentiment is shared across participant 1, a creative arts freelancer, who reflects on the value of digital skills in enhancing the marketability of their gig profiles: *“It’s definitely my art skills. Like, I get more and more of more of my clients based on my portfolios”.* The excerpt demonstrates how a strong digital skill set is visibly evident through completed gigs which can increase visibility among clients. This will enhance gig opportunities and lead to amplified market success that fulfils extrinsic motivational needs by generating higher gig income.

### 4.2. The interaction between intrinsic and extrinsic motivation

The findings reveal distinct patterns of interactions among intrinsic and extrinsic motivation, aligning with the findings of [35], which demonstrates that individuals can experience intrinsic and extrinsic motivation simultaneously. The participant accounts showcase two broad interaction patterns whereby they either align and mutually amplify engagement or dominate one another’s presence and influence engagement. It is notable that the interaction between both motivational forces is heavily shaped by digital skills which is instrumental in shaping perceptions of competency, feedback, and income.

### 4.3. Mutual existence among both motivational forces

This interaction pattern appeared as the most prevalent across the sample, where it is characterised by the mutual presence of both intrinsic and extrinsic motivation. The defining feature of this interaction is the simultaneous existence of both motivational conditions that converge to enhance engagement. The first dynamic appeared in accounts where workers recall genuine enjoyment and personal satisfaction arising from the gig itself, with external rewards supporting the overall motivational experience. Participant 14 coherently expressed this notion: *“Passion and other that that the money that I get after finishing the project influences the gigs I take”.* This excerpt demonstrates how engagement in gig work is primarily fueled by intrinsic passion whereby external rewards such as income earned supports this intrinsic drive by serving as a validation of competency. Participant 10 shares a similar perspective: *“The reason I prefer freelancing is mostly the freedom. Because when we are starting a nine to five, it’s like we are living for money, but actually we need money to live”.* The excerpt captures how the presence of autonomy in gig work, serves as the primary source of motivation which alters the relationship between work and money. The comparison of gig work to a 9−5 job highlights the distinction between working for financial survival vs working for passion where money is the ultimate result. In this instance, although the gig worker is primarily motivated by intrinsic needs, the presence of external rewards supports the overall motivational experience. Moreover, client feedback and praise shared the same notion where participants recall how positive feedback amplifies perceptions of competency. Participant 6, a video editor, notes: *“I think I’m very confident in my video editing skills with the praise I have been given. Yeah, the praise people have given me and also like what the clients have talked about. It has given me more confidence in my abilities as a video editor, especially using Adobe”.* This excerpt denotes the relationship between skill-based confidence and external validation, where a gig workers’ confidence is deeply rooted in their skillset which fuels their intrinsic drive. Hence, external praise from clients serves as a source of validation that strengthens feelings of competency and skill-based confidence. It is notable that this reciprocal loop was only attainable due to the existing skills of the gig worker which deemed sufficient to confidently produce a praiseworthy output. Participant 1 expands on this discourse by emphasising the importance of client feedback for professional development: *“I actually find it encouraging when they give me feedback that is positive or negative because that means that they really like care about the money that they invested in me. And they want you to turn good. So, if they give me an edit, that means that they genuinely love the thing I give them, you know. So it’s very motivating actually, the fact that they’re like invested in that way”.* The excerpt reflects on how both positive and negative feedback is entertained, with negative feedback being viewed as an opportunity for learning and growth. Accordingly, any constructive criticism received serves as an external validation with the perception that the client’s motive is rooted in self-improvement with the gig workers’ best interest in mind. Under such circumstances, intrinsic drive, which in this case is the gig workers sense of competency, is boosted through external praise and criticism. It is noteworthy that, once again, the gig worker’s skill set justified the client’s investment by rendering it meaningful rather than contingent.

Conversely, this dynamic also appears in extrinsic motivation where intrinsic drivers mutually support the overall motivation experience. As previously observed, digital skills serve as the principal mechanism underlying this relationship by connecting extrinsic drivers such as income to skill level. Participant 2 reflects on this notion: *“I mean, my earnings do depend on my skill level and, I guess, that would motivate me to increase my skill levels. The possibility of earning more if I do increase my skill level. So. Yeah, it does motivate me”.* The excerpt demonstrates the direct correlation between skills and gig earnings, where although income functions as a primary motivational driver, the recognition of digital skills as a critical asset simultaneously positions it as an intrinsic motivator. Hence, when income is perceived as skill dependent, it functions as a measure of professional competency rather than an external contingency. Participant 9 expands on this discourse by highlighting a dimension that is contingent to the Sri Lankan context: *“I prefer online freelancing. Because the more labor, the more money I earn. And, since the money I earn from freelancing is in dollars, it helps me a lot especially since I live in Sri Lanka with the inflation, I get more. I get more value out of dollars, when I’m doing freelancing, other than doing 9 to 5 job, because it only gives me a fixed salary. So I prefer doing freelancing”.* This excerpt captures the opportunity of earning in dollars which is particularly advantageous in the context of a developing country such as Sri Lanka. Not only does it provide gig workers with a financial gain, but it also exposes them to global markets that offer more value compared to the local context. This exposure carries intrinsic significance apart from financial value as it allows gig workers to earn rates that align with their skills and proficiencies in a context where local wages tend to undervalue digital gig work. Consequently, the professional skill validation arising from dollar earnings, strengthens the overall extrinsic motivation structure. Furthermore, participant 3 highlights the reputational aspect of this sentiment: *“Well, I’d say it’s not only the fact that I’m getting more income. It’s also the fact that, like, the more people commission me, the more they’ll tell their friends about me, and then they’ll tell their friends. It’s like a word of mouth thing that’s happening, which, I really, really like, you know, I need that for marketing because it’s the best kind of marketing, no matter what you do”.* The participant account reveals that apart from the income generated through additional gigs, the reputational significance created on their brand plays a key role in shaping motivation. Although income is largely extrinsic in origin, in such instances, it is experienced as a confirmation of professional value whereby the “word of mouth” activities serves as an affirming reminder that clients deem their work commendable enough to recommend to others.

### 4.4. Dominance among motivational forces

This interaction pattern between intrinsic and extrinsic motivation was notably concentrated among gig workers and contingent upon characteristics such as dependency on gig income and the manner in which feedback is received. This dynamic is analytically sound despite its lower occurrence as it highlights variations in how extrinsic and intrinsic motivational forces are experienced depending on the condition. The first category of gig workers resembles those who are heavily dependent on gig income due to financial pressure and income necessity. Participant 6 directly contributes to this discourse: *“Yeah, just, honestly, sometimes it’s just money. It’s like, money is like the main motivation. I mean, if there’s like, something I want to purchase for myself, like, okay, I’m going to get that. But I need to get the money for this. That’s why I want, to pick up more gigs”.* The excerpt demonstrates how gig workers who are primarily driven by financial necessity engage in gig work solely due to the pressure of earning a sufficient income. Hence, extrinsic motivation drivers such as rewards and bonuses tend to overweigh other motivational forces. Participant 5 expands this notion by reflecting on the stressful aspect: *“I know that no matter what, I’m going to get a certain amount of commission from everyone because it’s proven to be true. But there is an anxiety every month that like, what if I don’t make as much as last month. So it’s very.. it’s kind of inconsistent. So a little bit of anxiety is there”.* The excerpt captures how income volatility can shift the source of motivation from an autonomous engagement to an extrinsically dominated orientation fueled by financial needs. The participants account of “anxiety” was not a temporary occurrence but rather a consistent nature of gig work that permeates every gig interaction. Similarly, participant 8 contributes to this discourse by emphasising the impact of external evaluation such as client feedback on motivation: *“When the feedback is negative, naturally, I feel bad about it whenever I like the draft that I sent them. So whenever they want me to like sometimes completely change the whole thing. I feel bad about it and I feel unmotivated sometimes.”* This excerpt demonstrates how negative feedback can create performance anxiety replacing autonomous engagement with defensive behaviour. Gig workers that are heavily dependent on client feedback as their primary source of motivation, often view negative feedback as a threat to their competency instead of a learning opportunity. Hence, maintaining good ratings and the fear of losing clients tend to overcome any intrinsic motivational drive. A similar sentiment is shared across participant 10 who states: *“If it is a genuine mistake from my end or if they think it’s a mistake in their opinion, then it does kind of affect me because at the end of the day, it is also a representation of my work. I guess the broader truth is that the client will always want what the client wants”.* This excerpt reveals how genuine mistakes and client perceptions of mistakes may pose a threat to professional identity rather than serving as an opportunity for growth. It provides analytical significance as participant 10 also demonstrates mutual existence of both intrinsic and extrinsic motivation, suggesting that motivation is contextually shifting rather than fixed worker characteristics. It is notable that gig workers who display low intrinsic drive are often less concerned about continuous learning, display lower levels of confidence, and are more oriented towards financial rewards and positive client praise.

In contrast, the second category of gig workers represents those who primarily engage in gig work due to their genuine passion and interest for learning and evolvement. A noticeable dynamic in this condition is the role of digital skills in securing autonomous engagement with minimal dependency on external rewards. Participant 4 provides a direct account: *“I would say it’s not even about money at this point because I had client who wanted to pay me low but give me a lot of work. And I say that this is not about money but about skill, the passion that I have. And if it was about money then I would have gone and worked a 9-5 job”.* This excerpt demonstrates how intrinsic passion served as the primary basis for engaging in gig work whereby the ability to creatively apply digital skills overcame the financial benefits. The comparison to a traditional 9−5 is analytically significant as it entails that gig work offered the liberty to work with passion rather than financial necessity, a characteristic that is typically absent from corporate settings. Similarly, the role of financial independence emerged as a key factor driving autonomous engagement as stated by participant 13: *“Payments I receive from freelancing, if I work or not it’s just additional income for me. If I don’t, I still meet my needs. So, it doesn’t make any change”.* This excerpt reflects on the supplementary nature of gig income whereby external rewards are present, yet its dependency is significantly low as engagement is primarily self-driven. Since the participant fulfills their financial needs independent of gig income, motivation is sustained regardless of the presence of extrinsic drivers. Participant 11 expands the discourse on self-directed engagement by reflecting on the perks of gig work: *“So there are some reasons that I, that I prefer, online freelancing. Right. So we can set our own schedules, work from anywhere, and we can take breaks when we need. Right. So another thing that I prefer is that you are the manager. So you have the freedom to take decisions”.* This excerpt demonstrates how self-management such as the ability to decide when and where to work alongside independent decision making is intrinsically motivating. The absence of reference to any extrinsic drivers such as income and praise entails that the gig workers’ engagement is inherently driven by intrinsic forces such as autonomy.

### 4.5. The influence of motivational conditions on engagement

Engagement is defined as the active and sustained participation of gig workers in digital gig platforms and is often characterised by the persistent investment of time in gig work, decision to take on gigs, and consistent platform involvement [13]. The findings reveal that the interaction between intrinsic and extrinsic motivation significantly shapes platform engagement by functioning as mechanisms that either strengthen or weaken overall engagement. Workers that simultaneously experienced intrinsic and extrinsic motivation often notably emphasised the significance of platform familiarity in sustaining engagement. Participant 1 notes: *“If I’m looking for work, I guess it’s easier for me to look for work now because I use the platforms more. Whereas before I would struggle to find work sometimes early on. But now it’s like, like I kind of know where to go kind of thing”.* This excerpt denotes how accumulated platform familiarity minimises the hardships associated with gig discovery thereby transforming the experience from a source of struggle to an efficient process. The connection to both motivation types is notable as gig workers with intrinsic drive are more likely to invest in continuous learning to familiarise the platform which makes extrinsic drivers such as gig income feel more rewarding. Moreover, the magnitude of sustained engagement was depicted by participant 2’s account of completing more than 100 projects on the platform: *“It’s definitely more than 100 projects. Or, I don’t know, probably more than 100”*. The excerpt illustrates how investing long-term on the platform leads to accumulated experience which in turn became a core foundation for continued platform engagement. This indicates that each individual project completed strengthened platform knowledge, client relationship skills, and professional reputation, which thereby led to sustained participation at a high volume over time.

In contrast, participants whose motivation was characterised by dominance among intrinsic or extrinsic motivation often account fragile engagement that is vulnerable to disruption. Platform disruption clearly illustrated the weak engagement among gig workers whose sole motivation lies in extrinsic dominance. Participant 9 acknowledged: *“So if it if it’s a major update since since I do have a full time job, I don’t really have the full, flexibility or, the full freedom to, like, explore these tools. So, if it, it, let’s say, if it’s a major update, there would be a small time period, which I have to figure it out on my own, to search up and, to know exactly what is happening and how to, and how to handle the situation”.* The excerpt highlights how platform updates can disrupt platform familiarity and threaten continued platform engagement. Therefore, workers with poor adaptive digital skills or less relearning time experience higher vulnerability to their engagement. Conversely, participants that reflected an intrinsically dominant motivation structure demonstrates higher resilience in response to platform challenges as noted by participant 7: *“So, I think, obviously I came across certain challenges, but, I don’t remember specifics to tell you all the challenges, but, every time there is some challenge, you know, I just Google it or YouTube, I think it’s very easy to find resources to resolve these issues”*. The excerpt highlights the ease by which resolutions can be found, thereby providing analytical significance as intrinsically motivated gig workers may view platform challenges as a learning initiative rather than a threatening barrier. In such instances, technical difficulties served as an extension of existing intrinsic engagement. This serves as a notable contrast with participant 9’s vulnerability to platform changes, and directly highlights the underlying motivational environments that influence engagement. The same platform changes which improved the resilience of intrinsically motivated workers hindered the continued engagement of extrinsically controlled ones.

## 5. Discussion

The findings offer valuable insights into the role of digital skills in influencing the interaction between intrinsic and extrinsic motivation which ultimately shapes gig worker engagement. To further analyse and interpret these findings, the study proposes a motivation interplay matrix to conceptualise this interaction, followed by a motivation interplay typology to expand the conceptual findings.

### 5.1. Mapping digital skills, intrinsic, and extrinsic motivation into a motivation interplay matrix

**Fig 2:**
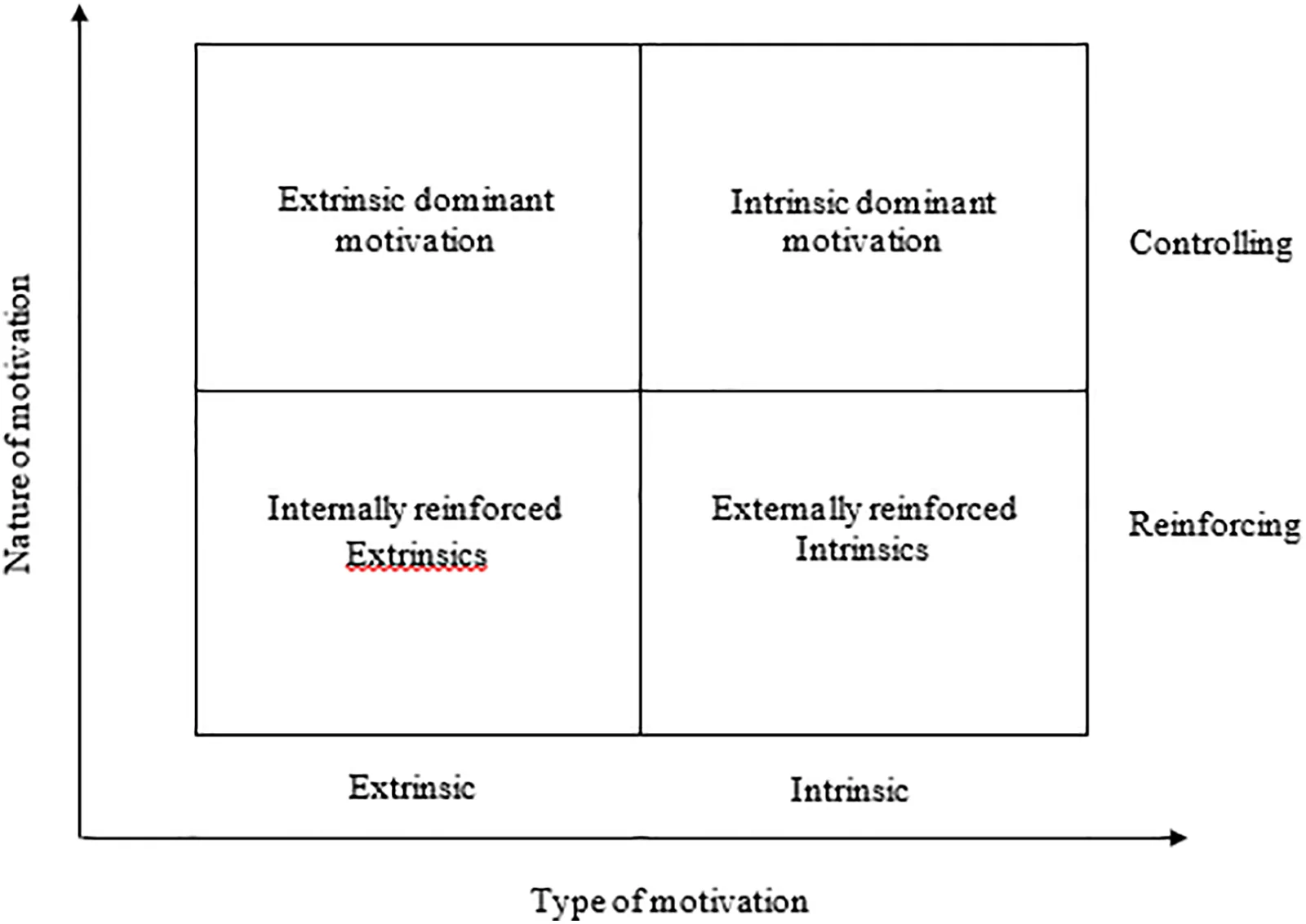
Motivation Interplay Matrix. (Source: Authors composition based on Thematic analysis results).

The findings imply that intrinsic and extrinsic motivation may act as a dynamic and interacting force, challenging past literature surrounding its dichotomous nature [27]. Across the participant accounts, digital skills emerged as a critical mechanism that shapes motivational outcomes which ultimately influences engagement in the digital gig economy. To capture this interaction in a systematic manner, this study introduces a motivation interplay matrix that conceptualises the coexistence and interaction of intrinsic and extrinsic motivation, whereby digital skills are positioned above the matrix as an antecedent mechanism. The framework is grounded in Self-Determination theory proposed by [27] alongside the critique of intrinsic and extrinsic dichotomy highlighted by [56]. The qualitative insights reflecting participants’ experiences of skill development, income, and client feedback on the overall motivational experience provided an empirical foundation to the frameworks’ development. To further develop an understanding on the interplay between intrinsic and extrinsic motivation, two critical axes were identified: 1) Nature of motivation 2) Type of motivation. Fig 2 illustrates the proposed motivation interplay matrix and Table 3 provides a linkage of the matrix quadrants to the participant accounts.

**Table 3:** Matrix evidence Table. (Source: Authors’ composition).

Quadrant	Interaction dynamic	Derivation	Participants	Representative quote
Extrinsic dominant	Extrinsic forces are experienced as pressure and an identity threat.	Derived from participant accounts describing chronic income anxiety and demotivation from negative feedback	P6, P5, P8, P10	*“Yeah, just, honestly, sometimes it’s just money. It’s like, money is like the main motivation. I mean, if there’s like, something I want to purchase for myself, like, okay, I’m going to get that. But I need to get the money for this. That’s why I want, to pick up more gigs”*
Intrinsic dominant	Intrinsic motivation is dominant to the point that extrinsic forces have no controlling power	Derived from participant accounts explicitly highlighting the importance of passion led work and strong financial independence	P4, P13,P11	*“Payments I receive from freelancing, if I work or not it’s just additional income for me. If I don’t, I still meet my needs. So, it doesn’t make any change”*
Internally reinforced extrinsics	Extrinsic rewards are internalised as signals of professional competence	Derived from participant accounts describing extrinsic rewards and intrinsic drivers in jointly enhancing motivation.	P2, p9, p3	*“I mean, my earnings do depend on my skill level and, I guess, that would motivate me to increase my skill levels. The possibility of earning more if I do increase my skill level. So. Yeah, it does motivate me”*
Externally reinforced intrinsics	Intrinsic motivation serves as the primary source of motivation whereby extrinsic rewards and feedback reinforce the motivation structure	Derived from participant accounts describing intrinsic drivers and extrinsic rewards in jointly enhancing motivation.	P14, p10, p6, p1	*“Passion and other that that the money that I get after finishing the project influences the gigs I take”*

#### 5.1.1. Digital skills as an antecedent mechanism.

Prior research often characterises digital skills as a technical capability or key labour market asset that is crucial to build resilience in digital platforms [13]. However, the current findings offer a novel perspective by demonstrating its function as an antecedent mechanism that shapes the way gig workers experience motivation. Gig workers with advanced proficiencies recall their motivational experiences being characterised by confidence, creativity, and professional growth. Whereas gig workers who recognised skill gaps reflected on how skill deficiencies constrained potential motivational rewards. The findings of this study substantiate the antecedent positioning of digital skills through three particular sub-mechanisms present across multiple participant accounts.

The first sub-mechanism is competence enablement whereby digital skills emerged as a critical factor that cultivates perceptions of competence and influences whether external forces are experienced as an affirmation or a threat. According to SDT theory, perceived competence serves as a critical element that drives autonomous motivation [27]. Consequently, the current findings demonstrate that engaging in gig work alone does not guarantee motivational rewards as proficient digital skills serve as the structural condition whereby perceptions of competence are developed. Participant 5 reflects on this sentiment by emphasising how a skill deficiency in video editing prevented them from taking on more gigs, which hindered both income and competence satisfaction from completing tasks. Similarly, participant 1 contributes to this discourse by sharing a similar view from the opposite direction whereby the gig workers’ strong art skills played a critical role in attracting clients. In such instances, strong digital skills established the condition for recurrent and motivationally rewarding gig interactions.

Moreover, cognitive liberation emerged as the second sub-mechanism referring to the motivational condition that arises when digital skills exhibit exceptional proficiency to the point of automaticity, thereby minimising cognitive load and directing it towards intrinsic dimensions of gig work. Hence, cognitive liberation determines the depth and quality of motivational rewards once the competence threshold has been met. For instance, participant 5’s account of digital tool mastery reflects on how strong proficiencies aided in producing muscle memory which ultimately contributed to a more efficient output. This indicates that when technical execution requires minimal attention, the motivational space of the gig interaction expands allowing gig workers to experience the intrinsic components of completing the task.

Motivational recursion emerged as the third sub-mechanism referring to the awareness of skill development in influencing the overall motivational experience. This awareness encourages persistent, self-directed skill development that amplifies motivation overtime. Participant 14 highlights this sub-dimension by reflecting on the significance of proactive skill development to meet various client needs rather than as an external requirement. Each client presents a new learning opportunity that enhances skill growth and ultimately contributes to a more rewarding future engagement. Similarly, participant 8’s account of recognising that stronger skills would enable a more efficient task completion connects the anticipated efficiency to a deeper motivational experience. Hence, skill development progressively shifts gig workers towards an autonomous motivational experience overtime.

#### 5.1.2. Nature of motivation: Reinforcing vs Controlling.

This axis captures the regulatory nature of motivation which can be narrowed down to two distinct forms: reinforcing and controlling. Reinforcing refers to instances whereby intrinsic and extrinsic motivation interact synergistically by supporting one another. Early literature proposes the idea that extrinsic motivation can combine with intrinsic motivation when a high initial level of intrinsic motivation exists [56]. This is applicable in the context of the gig economy as [37] finds that extrinsic incentives such as pay, and client feedback were often internalised and viewed as measures of competence and skills. Therefore, intrinsically motivated individuals may simultaneously experience extrinsic drivers which can reinforce internal enjoyment through mechanisms such as autonomy, competence, or relatedness. Conversely, this dynamic may also occur reciprocally whereby intrinsic motivation may sustain external drive, culminating a mutual reinforcement. Although extrinsic motivation is often characterized as being driven by external incentives [35], recent studies suggest that extrinsic rewards can become internalized ultimately leading to autonomous engagement [57].

Moreover, the Controlling dimension refers to forms of motivation, either intrinsic or extrinsic, that are heavily dominant, and pressuring as opposed to reinforcing or interacting with one another. This controlling nature of motivation is said to arise in chaotic or neglectful environments which can create pressure externally [58]. External pressure may arise from the need to meet deadlines, evaluations, or earn rewards which can force compliance over self-endorsement [28]. For instance, gig workers may engage in gig work purely to secure their financial needs and avoid negative consequences. Therefore, the controlling dimension allows for the distinction between extrinsic rewards that are detrimental to autonomy, and those which can be internalized. However, intrinsic control may arise when workers are primarily motivated by the inherent satisfaction gained from their work as opposed to financial rewards or pressures [36]. Drawing on SDT, gig workers who prefer high levels of autonomy, skill mastery, and learning are less likely to be influenced by external factors such as rewards and incentives.

#### 5.1.3. Type of motivation: Intrinsic vs Extrinsic.

This axis captures the source of motivation ranging from extrinsic to intrinsic motivation, drawn from SDT theory [27]. It helps highlight the source behind what motivates gig workers such as internal satisfaction or external outcomes. The intrinsic dimension captures intrinsic motivation which refers to an autonomous motivation where individuals engage in activities due to their inherent interest and satisfaction in completing a task as opposed to fulfilling obligations [36]. Whereas the extrinsic dimension captures extrinsic motivation which arises when an activity is conducted in an attempt to achieve a separable outcome [36]. Importantly, this axis highlights that gig workers may often experience intrinsic and extrinsic motivation simultaneously by capturing the dominance of each motivation form rather than its exclusivity.

### 5.2. Quadrants of the Motivation interplay matrix

The Motivation Interplay Matrix conceptualises the interplay of gig worker motivation along two dimensions: regulatory nature of motivation and source of motivation. This intersection manifests into four distinct quadrants with each displaying the trajectory of how gig workers interact, experience, and respond to motivational forces in digital platforms.

#### 5.2.1. Extrinsic and controlling.

This quadrant represents motivation that is extrinsically dominant and controlling in nature, where behaviour is heavily influenced by external demands or constraints. These demands may arise from external pressures such as deadlines, client feedback, and income necessity which constrains autonomy. Motivation in this quadrant is sustained through compliance and obligation instead of inherent internal endorsement. The analytical significance of this quadrant lies not in the presence of extrinsic motivation itself, but in the question of why external forces provided reinforcing experiences for some gig workers while providing controlling experiences for others. Across participant accounts, income necessity emerged as a central controlling mechanism that permeates every gig interaction due to consistent pressure. Participant 5’s account of chronic anxiety due to the uncertainty of gig earnings serves as a prominent example. as the gig worker’s reliance on platform earnings as their primary source of income, alongside skill deficiencies in video editing contribute to this controlling condition. Similarly, evaluative pressure from client feedback contributes to this quadrant by producing genuine personal distress. Participant 8’s account of receiving negative client feedback which immediately hindered motivation demonstrates that external evaluations were internalised as indicators of personal failure instead of learning opportunities. Participant 10 expands this discourse by highlighting the impact of perceived work mistakes as a threat to professional identity, sharing the same level of vulnerability as participant 5. Across both participant accounts, it is notable that the absence of proficient digital skills played a key role in the manner that evaluative feedback was received. A gig worker that possesses strong digital skills may hold a strong competence foundation which can minimise vulnerability to negative feedback. Hence, the absence of this foundation will lead to negative feedback being penetrated more deeply which will ultimately produce the identity threat described by participant 10 and demotivation experienced by participant 8. A notable feature of this quadrant is that intrinsic factors may exert minimal influence leading to instrumental engagement and heavy dependence on external enforcement. The relative scarcity of this motivational profile reflects a limitation in sample size rather than a rarity of the condition itself in gig work.

#### 5.2.2. Intrinsic and controlling.

This quadrant reflects motivation that is predominantly intrinsic and self-driven with minimal reliance on external rewards or demands. Individuals in this category are motivated by the intrinsic satisfaction gained from autonomy, skill mastery, or fulfilment with tasks completed. Financial independence from gig earnings emerged as a key structural enabler of this condition which neutralises the controlling potential of platform income. Participant 13’s account of being able to meet their financial needs regardless of gig earnings demonstrates the nature of engagement whereby gigs are undertaken for mere personal fulfilment. Hence, participant 5’s chronic anxiety due to income volatility is unapplicable to participant 13, as the financial stakes are categorically different. Similarly, selective gig curation reflected by participant 4’s account highlights how engagement in gig work is purely intrinsic driven whereby the ability to passionately apply their skills to the gigs undertaken, serves as the primary motive. Hence, it is notable that motivation in this quadrant sustains independent of external rewards, demonstrating that gig work can be a result of self-directed participation rather than a reward-based obligation.

#### 5.2.3. Intrinsic and reinforcing.

This quadrant captures a motivation structure where both intrinsic and extrinsic motivation is simultaneously present, yet engagement is primarily driven by intrinsic interest such as skill mastery, flexibility, and fulfilment with extrinsic motivational drivers functioning in a supportive and reinforcing manner. Individuals in this quadrant primarily engage in work due to inherent interest and satisfaction whereby extrinsic rewards play a supplemental and secondary role. Participant 14’s account of passion being the primary source of gig engagement with gig income playing a supporting role reflects this notion. Motivation is sustained because extrinsic factors align with internal values and interests, creating a reinforcing loop without being the primary source of motivation. Similarly, extrinsic drivers such as client feedback and rewards are perceived as informational and supplemental rather than evaluative, which strengthens feelings of competency. Participant 6’s account highlights how intrinsic skill confidence plays a key role in driving motivation whereby client praise enhances the existing skill confidence. This amplification was possible due to participant 6’s existing skill proficiency that served as a competence foundation. Hence, the external praise received served as a competence confirmation demonstrating that digital skills are crucial for the amplification of intrinsic motivation. A notable feature of this quadrant is that reinforcement occurs through affirmative growth rather than external pressures or demands.

#### 5.2.4. Extrinsic and reinforcing.

This quadrant reflects a motivation structure whereby external incentives are primary drivers of engagement, yet they are internalised as it is deemed meaningful to individuals. Although this motivation structure remains extrinsically dominant, external incentives such as rewards and client feedback are perceived by individuals as useful to their personal development and career growth. Participant 2’s account captures this directly by highlighting the significance of skill improvement for increasing potential gig income. This indicates that earnings are dependent on skill level which creates the intrinsic drive for self-improvement. Thus, external rewards may function as indicators of competence rather than pressure or control mechanisms. Participant 9’s account offers contextual significance to this quadrant by demonstrating the advantage of dollar earnings not only as a currency advantage but as a marker of participation in a global setting. The contextual amplification deepens engagement as earning in dollars provides a positioning that reinforces a sense of competency. Therefore, conceptually this quadrant highlights how extrinsic motivation can coexist with intrinsic motivation as reinforcement occurs when external rewards are viewed as supporting autonomy instead of constraining it.

### 5.3. Typology of the motivation interplay in the digital gig economy

To expand the understanding of the interplay between extrinsic and intrinsic motivation in the digital gig economy, the study proposes a typology of motivation interplay that advances the conceptual findings of the motivation interplay matrix. A typology enables the creation of classifications and categories by narrowing down a variety of examples [59], that are typically derived through conceptualisation [60]. The proposed typology in Table 4 allows for critical analysis of the motivation interplay quadrants and reflects how gig workers respond and interact with motivational cues, creating a variety of engagement outcomes in the digital gig economy.

**Table 4:** Motivation Interplay Typology. (Source: Authors’ Composition based on thematic analysis output).

Typology profile	Motivation configuration	Core characteristics	Engagement outcomes
Extrinsic Dominant profile	Dominant Extrinsic Motivation & low intrinsic influence	Engagement in primarily driven by income necessity and performance pressures creating limited space for intrinsic fulfilment	Need based and fragile engagement arising from financial burdens and pressure, often vulnerable to burnout
Intrinsic Dominant profile	Dominant Intrinsic motivation & low extrinsic influence	Engagement is primarily driven by the need for continuous learning, growth, and enjoyment. Extrinsic rewards may have limited influence in driving engagement	Self-directed engagement cultivated by the need for continuous learning and psychological fulfilment.
Internally reinforced Extrinsics	High extrinsic motivation & intrinsic reinforcement	Engagement is dominantly driven by extrinsic cues such as income and feedback which are internalised and perceived as aligning with personal goals	Strategic engagement arising from financial success being a reflection of professional identity
Externally reinforced Intrinsics	High intrinsic motivation & extrinsic reinforcement	Engagement is dominantly driven by intrinsic motivation with extrinsic factors such as client feedback and income reinforcing motivation.	High intensity, consistent engagement sustained through skill development and work commitment.

## 6. Implications for practice

### 6.1. Theoretical implications and contributions

This study offers numerous theoretical contributions to existing literature surrounding the digital skills, gig worker motivation, and platform engagement. First, the motivation interplay matrix contextualises Self-Determination Theory [27] within the platform-based gig economy of Sri Lanka by demonstrating the role of digital skills in shaping the interaction between intrinsic and extrinsic motivation. Although subsequent revisions to SDT theory have demonstrated that intrinsic and extrinsic motivation can occur on a continuum [35], existing gig economy literature characterises both motivation components as competing or substituting one another. For instance [16], suggests that leveraging intrinsic motivation is substantially more advantageous compared to extrinsic motivation. Whereas [37] argues that extrinsic motivation is particularly salient in influencing a gig workers choice to enter and engage in digital gig work. This pattern of literature with an isolated focus on intrinsic or extrinsic motivation creates a critical gap as prior studies have not empirically examined the role of digital skills in shaping how both motivation components may interact in the digital gig economy. In fact, this study responds to the suggestion of [16] who stated that “future work exploring motivation within the gig-economy should consider the dynamics between these types of motivation”, referring to intrinsic and extrinsic motivation. Therefore, the motivation interplay matrix provides fresh insights about the dynamic interplay between intrinsic and extrinsic motivation, demonstrating that both forces can coexist through mutual reinforcement or control one another’s presence. The framework contextualises SDT theory [27] and combines the critique of intrinsic extrinsic dichotomy by [56], to demonstrate that gig workers can internalise external rewards, recognition, and feedback. This contribution is particularly significant in the context of the digital gig economy as it provides a psychologically grounded understanding of motivation.

Second, the motivation interplay typology provides valuable insights with regards to gig worker engagement. Prior literature has examined the effects of gamification [29], intrinsic motivation [16], and extrinsic incentives on platform engagement, often treating engagement as a static outcome. Therefore, the motivation interplay typology contributes to existing literature by demonstrating that engagement varies from autonomous to self-regulated depending on the motivation condition. The four motivational profiles share similarities with [32] five category typology with the most relevant comparison being between the “Dabbler” profile and intrinsic dominant profile in the current study. While “Dabblers” share similarities with intrinsic dominant gig workers in terms of being less financially precarious, the current study reveals that intrinsic dominant workers are heavily governed by passion, creative autonomy, and well-being orientation which serves as a contrast to the passive non-attachment that governs “Dabblers”. Thus, the typology proposes that engagement in the digital gig economy is conceptualised as an evolving and context dependent process, influenced by interactions between digital skills and motivation. Third, despite digital skills being widely explored in terms of its technical competencies, its psychological aspect and impact remains relatively unknown. Prior research finds that digital skills serve as a core component in sustaining engagement in digital gig work by [13, 15], as digital skills are instrumental for employability and productivity [12]. While these perspectives are valuable, they often overlook the multidimensional nature of digital skills whereby skills can serve as motivational resources. This study expands past literature by demonstrating that digital skills foster autonomy, competence, and relatedness, incorporating a motivational lens to digital skills. In doing so, this study positions the relevance of digital skills beyond technical competencies into the domain of psychological engagement in digital gig work.

### 6.2. Practical implications

This study offers numerous practical implications for platform designers, policymakers, and digital gig workers. First, the motivation interplay matrix offers valuable insights to platform designers with regards to platform design and rewards schemes. Platforms can benefit from reinforcement-oriented motivation designs rather than extrinsically controlling features such as excessive algorithmic surveillance and pressure bound ratings and scores for gig worker motivation. For instance, platforms can incorporate features such as transparent performance metrics, growth-oriented feedback systems, and mechanisms for gig worker recognition that will internalise external rewards and reinforce autonomy and competence, instead of constraining it. Moreover, the motivation interplay typology presents opportunities to curate engagement strategies tailored to various motivational profiles, which will ultimately contribute to gig worker loyalty and retention within platforms. Second, this study offers actionable insights to gig workers to manage their individual motivational trajectories. It demonstrates that developing a strong digital skill set can help perceive extrinsic rewards as a reflection of competence and professional growth rather than an external motivator driven by financial necessity. This shift in perception will enhance job satisfaction and contribute to long term engagement. Third, the study provides valuable insights to policymakers to design interventions that promote inclusive and decent digital work. Platforms should be monitored to protect gig workers from excessively controlling algorithmic management which can constrain autonomy and lead to burn out. Ultimately, it will drive successful participation aligning with the scope of SDG 8.

## 7. Conclusion and limitations

This study set out to examine how digital skills may shape motivational processes and engagement in the digital gig economy. Drawing onto scholarly inquiry into the psychological aspect and motivational structures of gig work, this study explores the dynamic interplay between intrinsic and extrinsic motivation while examining the role of digital skills as a key linking mechanism between motivation and engagement. Although this study provides valuable insights into digital skills, motivation, and engagement in the digital gig economy, it is subject to several limitations. First, the research was focused solely on platform-based gig workers located in Sri Lanka, whose opinions and dialect may reflect Sri Lankan culture and working expectations. Thus, the applicability of the Motivation interplay typology to gig workers outside Sri Lanka, is relatively unknown. Second, the sample consisted of predominantly young digital gig workers concentrated in fields such as creative and technical platform work, with a large majority engaging in gig work as a part-time source of income. Therefore, the participant accounts may largely reflect the experiences of gig workers who possess greater occupational autonomy and flexibility than gig workers who exclusively depend on the platform as their primary source of income. This serves as a limitation particularly for the extrinsic-controlling motivational profile which captures the experiences of gig workers under high financial necessity. Hence, future research can offer deeper insights into this motivational profile by examining whether the current findings are replicated among workers with high platform dependence or algorithmic pressure. Third, the focus on digital platform-based gig workers limits the applicability of the findings to that specific segment of gig workers. Therefore, the findings may not account for other gig workers such as corporate gig workers, ride-share drivers, or food delivery couriers. Lastly, the cross-sectional nature of data analysed in this study provides a snapshot in time. Thus, a longitudinal perspective would offer more rich and deeper insights into motivational regulation and engagement over time. Future research could delve into the influence of algorithmic processes on intrinsic and extrinsic motivation in the digital gig economy.

## Supporting information

S1 File Nvivo codebook. (DOCX)

S2 File Codebook. (XLSX)
