# Supplementary material for: What keeps gig workers motivated? A typology of the intrinsic-extrinsic motivation interplay in the digital gig economy
Source: PLoS One. 2026 Sep 22;21(9):e0345881. doi: 10.1371/journal.pone.0345881 (PMC13596809; doi:10.1371/journal.pone.0345881)
Supplement: Supplementary file 1 [file pone.0345881.s001.docx]

| 6/18/2026 4:13 PM | | | | | | | | | | | | | | | | | |
| --- | --- | --- | --- | --- | --- | --- | --- | --- | --- | --- | --- | --- | --- | --- | --- | --- | --- |
| Coding Summary by Code | | | | | | | | | | | | | | | | | |
| Journal Paper - Motivation | | | | | | | | | | | | | | | | | |
| 6/18/2026 4:13 PM | | | | | | | | | | | | | | | | | |
|  | | | **Aggregate** |  | **Classification** |  | **Coverage** |  | **Number Of Coding References** | |  | **Reference Number** |  | **Coded By Initials** |  | **Modified On** |  |
| **Code** | | | | | | | | | | | | | | | | |  |
|  | **Codes\\Digital Skills** | | | | | | | | | | | | | | | |  |
|  | | **Document** | | | | | | | | | | | | | | |  |
|  | | | **Files\\Participant 1** | | | | | | | | | | | | | |  |
|  |  |  | No |  |  |  | 0.0267 |  | 7 | |  | | | | | |  |
|  | | |  |  |  |  |  |  |  | |  | | | | | | |
|  | | | | | | | | | | | | 1 |  | MSD |  | 6/18/2026 2:56 PM |  |
|  | I would say it, communication is a pretty important thing because, you need to be good clients and, like, try to understand, like what exactly they need, like in terms of, like, the character descriptions and things like that. So there's always, like, back and forth with, like, feedback. I like if there's a change for a specific part of the character. | | | | | | | | | | | | | | | |  |
|  |  | | | | | | | | | | | | | | | |  |
|  | | | | | | | | | | | | 2 |  | MSD |  | 6/18/2026 2:56 PM |  |
|  | communication is a big thing | | | | | | | | | | | | | | | |  |
|  |  | | | | | | | | | | | | | | | |  |
|  | | | | | | | | | | | | 3 |  | MSD |  | 6/18/2026 2:56 PM |  |
|  | studio paint. | | | | | | | | | | | | | | | |  |
|  |  | | | | | | | | | | | | | | | |  |
|  | | | | | | | | | | | | 4 |  | MSD |  | 6/18/2026 2:56 PM |  |
|  | Photoshop | | | | | | | | | | | | | | | |  |
|  |  | | | | | | | | | | | | | | | |  |
|  | | | | | | | | | | | | 5 |  | MSD |  | 6/18/2026 2:56 PM |  |
|  | drawing skills | | | | | | | | | | | | | | | |  |
|  |  | | | | | | | | | | | | | | | |  |
|  | | | | | | | | | | | | 6 |  | MSD |  | 6/18/2026 2:56 PM |  |
|  | good communication skills is, like, pretty important | | | | | | | | | | | | | | | |  |
|  |  | | | | | | | | | | | | | | | |  |
|  | | | | | | | | | | | | 7 |  | MSD |  | 6/18/2026 2:56 PM |  |
|  | I like getting to know that I had to do that, rather was something I learned and trying to like, make sure I do that next time | | | | | | | | | | | | | | | |  |
|  |  | | | | | | | | | | | | | | | |  |
|  | | | **Files\\Participant 10** | | | | | | | | | | | | | |  |
|  |  |  | No |  |  |  | 0.0243 |  | 9 | |  | | | | | |  |
|  | | |  |  |  |  |  |  |  | |  | | | | | | |
|  | | | | | | | | | | | | 1 |  | MSD |  | 6/18/2026 2:56 PM |  |
|  | graphic designing | | | | | | | | | | | | | | | |  |
|  |  | | | | | | | | | | | | | | | |  |
| Formatted Reports\\Coding Summary by Code Formatted Report | | | | | | | | | | Page 1 of 36 | | | | | | | |
| 6/18/2026 4:13 PM | | | | | | | | | | | | | | | | | |
|  | | | **Aggregate** |  | **Classification** |  | **Coverage** |  | **Number Of Coding References** | |  | **Reference Number** |  | **Coded By Initials** |  | **Modified On** |  |
|  | | | | | | | | | | | | | | | | | |
|  | | | | | | | | | | | | 2 |  | MSD |  | 6/18/2026 2:56 PM |  |
|  | ui/ux designing | | | | | | | | | | | | | | | |  |
|  |  | | | | | | | | | | | | | | | |  |
|  | | | | | | | | | | | | 3 |  | MSD |  | 6/18/2026 2:56 PM |  |
|  | social media post designing | | | | | | | | | | | | | | | |  |
|  |  | | | | | | | | | | | | | | | |  |
|  | | | | | | | | | | | | 4 |  | MSD |  | 6/18/2026 2:56 PM |  |
|  | logo designing | | | | | | | | | | | | | | | |  |
|  |  | | | | | | | | | | | | | | | |  |
|  | | | | | | | | | | | | 5 |  | MSD |  | 6/18/2026 2:56 PM |  |
|  | flyer designs | | | | | | | | | | | | | | | |  |
|  |  | | | | | | | | | | | | | | | |  |
|  | | | | | | | | | | | | 6 |  | MSD |  | 6/18/2026 2:56 PM |  |
|  | Banner designs | | | | | | | | | | | | | | | |  |
|  |  | | | | | | | | | | | | | | | |  |
|  | | | | | | | | | | | | 7 |  | MSD |  | 6/18/2026 2:56 PM |  |
|  | figma | | | | | | | | | | | | | | | |  |
|  |  | | | | | | | | | | | | | | | |  |
|  | | | | | | | | | | | | 8 |  | MSD |  | 6/18/2026 2:56 PM |  |
|  | Photoshop | | | | | | | | | | | | | | | |  |
|  |  | | | | | | | | | | | | | | | |  |
|  | | | | | | | | | | | | 9 |  | MSD |  | 6/18/2026 2:56 PM |  |
|  | Actually uh, use more the good communication helps us to do the gig more quickly and easily, both because it's clear for the both parties to what they need and what we are going to do. So the clearer the communication gets, it the easier the weekend finished work. | | | | | | | | | | | | | | | |  |
|  |  | | | | | | | | | | | | | | | |  |
|  | | | **Files\\Participant 11** | | | | | | | | | | | | | |  |
|  |  |  | No |  |  |  | 0.0055 |  | 8 | |  | | | | | |  |
|  | | |  |  |  |  |  |  |  | |  | | | | | | |
|  | | | | | | | | | | | | 1 |  | MSD |  | 6/18/2026 2:56 PM |  |
|  | software development | | | | | | | | | | | | | | | |  |
|  |  | | | | | | | | | | | | | | | |  |
|  | | | | | | | | | | | | 2 |  | MSD |  | 6/18/2026 2:56 PM |  |
|  | website development | | | | | | | | | | | | | | | |  |
|  |  | | | | | | | | | | | | | | | |  |
|  | | | | | | | | | | | | 3 |  | MSD |  | 6/18/2026 2:56 PM |  |
|  | machine learning | | | | | | | | | | | | | | | |  |
|  |  | | | | | | | | | | | | | | | |  |
|  | | | | | | | | | | | | 4 |  | MSD |  | 6/18/2026 2:56 PM |  |
|  | mobile application development | | | | | | | | | | | | | | | |  |
|  |  | | | | | | | | | | | | | | | |  |
|  | | | | | | | | | | | | 5 |  | MSD |  | 6/18/2026 2:56 PM |  |
|  | JavaScript | | | | | | | | | | | | | | | |  |
|  |  | | | | | | | | | | | | | | | |  |
|  | | | | | | | | | | | | 6 |  | MSD |  | 6/18/2026 2:56 PM |  |
|  | .Net | | | | | | | | | | | | | | | |  |
|  |  | | | | | | | | | | | | | | | |  |
| Formatted Reports\\Coding Summary by Code Formatted Report | | | | | | | | | | Page 2 of 36 | | | | | | | |
| 6/18/2026 4:13 PM | | | | | | | | | | | | | | | | | |
|  | | | **Aggregate** |  | **Classification** |  | **Coverage** |  | **Number Of Coding References** | |  | **Reference Number** |  | **Coded By Initials** |  | **Modified On** |  |
|  | | | | | | | | | | | | | | | | | |
|  | | | | | | | | | | | | 7 |  | MSD |  | 6/18/2026 2:56 PM |  |
|  | Android Studio. | | | | | | | | | | | | | | | |  |
|  |  | | | | | | | | | | | | | | | |  |
|  | | | | | | | | | | | | 8 |  | MSD |  | 6/18/2026 2:56 PM |  |
|  | JavaScript | | | | | | | | | | | | | | | |  |
|  |  | | | | | | | | | | | | | | | |  |
|  | | | **Files\\Participant 12** | | | | | | | | | | | | | |  |
|  |  |  | No |  |  |  | 0.0035 |  | 4 | |  | | | | | |  |
|  | | |  |  |  |  |  |  |  | |  | | | | | | |
|  | | | | | | | | | | | | 1 |  | MSD |  | 6/18/2026 2:56 PM |  |
|  | web development | | | | | | | | | | | | | | | |  |
|  |  | | | | | | | | | | | | | | | |  |
|  | | | | | | | | | | | | 2 |  | MSD |  | 6/18/2026 2:56 PM |  |
|  | Graphic designing | | | | | | | | | | | | | | | |  |
|  |  | | | | | | | | | | | | | | | |  |
|  | | | | | | | | | | | | 3 |  | MSD |  | 6/18/2026 2:56 PM |  |
|  | digital marketing | | | | | | | | | | | | | | | |  |
|  |  | | | | | | | | | | | | | | | |  |
|  | | | | | | | | | | | | 4 |  | MSD |  | 6/18/2026 2:56 PM |  |
|  | WordPress development | | | | | | | | | | | | | | | |  |
|  |  | | | | | | | | | | | | | | | |  |
|  | | | **Files\\Participant 13** | | | | | | | | | | | | | |  |
|  |  |  | No |  |  |  | 0.0034 |  | 5 | |  | | | | | |  |
|  | | |  |  |  |  |  |  |  | |  | | | | | | |
|  | | | | | | | | | | | | 1 |  | MSD |  | 6/18/2026 2:56 PM |  |
|  | account management | | | | | | | | | | | | | | | |  |
|  |  | | | | | | | | | | | | | | | |  |
|  | | | | | | | | | | | | 2 |  | MSD |  | 6/18/2026 2:56 PM |  |
|  | research | | | | | | | | | | | | | | | |  |
|  |  | | | | | | | | | | | | | | | |  |
|  | | | | | | | | | | | | 3 |  | MSD |  | 6/18/2026 2:56 PM |  |
|  | client communications | | | | | | | | | | | | | | | |  |
|  |  | | | | | | | | | | | | | | | |  |
|  | | | | | | | | | | | | 4 |  | MSD |  | 6/18/2026 2:56 PM |  |
|  | Capcut | | | | | | | | | | | | | | | |  |
|  |  | | | | | | | | | | | | | | | |  |
|  | | | | | | | | | | | | 5 |  | MSD |  | 6/18/2026 2:56 PM |  |
|  | TikTok | | | | | | | | | | | | | | | |  |
|  |  | | | | | | | | | | | | | | | |  |
| Formatted Reports\\Coding Summary by Code Formatted Report | | | | | | | | | | Page 3 of 36 | | | | | | | |
| 6/18/2026 4:13 PM | | | | | | | | | | | | | | | | | |
|  | | | **Aggregate** |  | **Classification** |  | **Coverage** |  | **Number Of Coding References** | |  | **Reference Number** |  | **Coded By Initials** |  | **Modified On** |  |
|  | | | **Files\\Participant 14** | | | | | | | | | | | | | |  |
|  |  |  | No |  |  |  | 0.0147 |  | 1 | |  | | | | | |  |
|  | | |  |  |  |  |  |  |  | |  | | | | | | |
|  | | | | | | | | | | | | 1 |  | S |  | 6/18/2026 3:12 PM |  |
|  | I think it helps me to like, build up my, like, knowledge and technical skills, what I use, in like, day to day. So like, it helps me a lot to do my 9 to 5 work and stuff because I'm doing extra projects for different clients. So it will be like a new experience for me. | | | | | | | | | | | | | | | |  |
|  |  | | | | | | | | | | | | | | | |  |
|  | | | **Files\\Participant 14** | | | | | | | | | | | | | |  |
|  |  |  | No |  |  |  | 0.0083 |  | 13 | |  | | | | | |  |
|  | | |  |  |  |  |  |  |  | |  | | | | | | |
|  | | | | | | | | | | | | 1 |  | MSD |  | 6/18/2026 2:56 PM |  |
|  | VBA coding | | | | | | | | | | | | | | | |  |
|  |  | | | | | | | | | | | | | | | |  |
|  | | | | | | | | | | | | 2 |  | MSD |  | 6/18/2026 2:56 PM |  |
|  | automations | | | | | | | | | | | | | | | |  |
|  |  | | | | | | | | | | | | | | | |  |
|  | | | | | | | | | | | | 3 |  | MSD |  | 6/18/2026 2:56 PM |  |
|  | Power Automate | | | | | | | | | | | | | | | |  |
|  |  | | | | | | | | | | | | | | | |  |
|  | | | | | | | | | | | | 4 |  | MSD |  | 6/18/2026 2:56 PM |  |
|  | Microsoft forms | | | | | | | | | | | | | | | |  |
|  |  | | | | | | | | | | | | | | | |  |
|  | | | | | | | | | | | | 5 |  | MSD |  | 6/18/2026 2:56 PM |  |
|  | robotic process automation | | | | | | | | | | | | | | | |  |
|  |  | | | | | | | | | | | | | | | |  |
|  | | | | | | | | | | | | 6 |  | MSD |  | 6/18/2026 2:56 PM |  |
|  | UiPath | | | | | | | | | | | | | | | |  |
|  |  | | | | | | | | | | | | | | | |  |
|  | | | | | | | | | | | | 7 |  | MSD |  | 6/18/2026 2:56 PM |  |
|  | Excel | | | | | | | | | | | | | | | |  |
|  |  | | | | | | | | | | | | | | | |  |
|  | | | | | | | | | | | | 8 |  | MSD |  | 6/18/2026 2:56 PM |  |
|  | yellow AI | | | | | | | | | | | | | | | |  |
|  |  | | | | | | | | | | | | | | | |  |
|  | | | | | | | | | | | | 9 |  | MSD |  | 6/18/2026 2:56 PM |  |
|  | Azure DevOps | | | | | | | | | | | | | | | |  |
|  |  | | | | | | | | | | | | | | | |  |
|  | | | | | | | | | | | | 10 |  | MSD |  | 6/18/2026 2:56 PM |  |
|  | Visio | | | | | | | | | | | | | | | |  |
|  |  | | | | | | | | | | | | | | | |  |
|  | | | | | | | | | | | | 11 |  | MSD |  | 6/18/2026 2:56 PM |  |
|  | Copilot studio | | | | | | | | | | | | | | | |  |
|  |  | | | | | | | | | | | | | | | |  |
|  | | | | | | | | | | | | 12 |  | MSD |  | 6/18/2026 2:56 PM |  |
|  | Coding. | | | | | | | | | | | | | | | |  |
|  |  | | | | | | | | | | | | | | | |  |
| Formatted Reports\\Coding Summary by Code Formatted Report | | | | | | | | | | Page 4 of 36 | | | | | | | |
| 6/18/2026 4:13 PM | | | | | | | | | | | | | | | | | |
|  | | | **Aggregate** |  | **Classification** |  | **Coverage** |  | **Number Of Coding References** | |  | **Reference Number** |  | **Coded By Initials** |  | **Modified On** |  |
|  | | | | | | | | | | | | | | | | | |
|  | | | | | | | | | | | | 13 |  | MSD |  | 6/18/2026 2:56 PM |  |
|  | Mostly fixing bugs. | | | | | | | | | | | | | | | |  |
|  |  | | | | | | | | | | | | | | | |  |
|  | | | **Files\\Participant 2** | | | | | | | | | | | | | |  |
|  |  |  | No |  |  |  | 0.0022 |  | 2 | |  | | | | | |  |
|  | | |  |  |  |  |  |  |  | |  | | | | | | |
|  | | | | | | | | | | | | 1 |  | MSD |  | 6/18/2026 2:56 PM |  |
|  | only my art skills | | | | | | | | | | | | | | | |  |
|  |  | | | | | | | | | | | | | | | |  |
|  | | | | | | | | | | | | 2 |  | MSD |  | 6/18/2026 2:56 PM |  |
|  | It's defenitly my art skills | | | | | | | | | | | | | | | |  |
|  |  | | | | | | | | | | | | | | | |  |
|  | | | **Files\\Participant 3** | | | | | | | | | | | | | |  |
|  |  |  | No |  |  |  | 0.0081 |  | 6 | |  | | | | | |  |
|  | | |  |  |  |  |  |  |  | |  | | | | | | |
|  | | | | | | | | | | | | 1 |  | MSD |  | 6/18/2026 2:56 PM |  |
|  | basic communication skills | | | | | | | | | | | | | | | |  |
|  |  | | | | | | | | | | | | | | | |  |
|  | | | | | | | | | | | | 2 |  | MSD |  | 6/18/2026 2:56 PM |  |
|  | Photoshop | | | | | | | | | | | | | | | |  |
|  |  | | | | | | | | | | | | | | | |  |
|  | | | | | | | | | | | | 3 |  | MSD |  | 6/18/2026 2:56 PM |  |
|  | I had to make some sacrifices. I mean, when it comes to work, I do also like stay up and finish it off. I was, like, working extra hours. I was working extra hours on my day off. | | | | | | | | | | | | | | | |  |
|  |  | | | | | | | | | | | | | | | |  |
|  | | | | | | | | | | | | 4 |  | MSD |  | 6/18/2026 2:56 PM |  |
|  | Adobe Suite | | | | | | | | | | | | | | | |  |
|  |  | | | | | | | | | | | | | | | |  |
|  | | | | | | | | | | | | 5 |  | MSD |  | 6/18/2026 2:56 PM |  |
|  | Photoshop | | | | | | | | | | | | | | | |  |
|  |  | | | | | | | | | | | | | | | |  |
|  | | | | | | | | | | | | 6 |  | MSD |  | 6/18/2026 2:56 PM |  |
|  | graphic design | | | | | | | | | | | | | | | |  |
|  |  | | | | | | | | | | | | | | | |  |
|  | | | **Files\\Participant 4** | | | | | | | | | | | | | |  |
|  |  |  | No |  |  |  | 0.0098 |  | 10 | |  | | | | | |  |
|  | | |  |  |  |  |  |  |  | |  | | | | | | |
|  | | | | | | | | | | | | 1 |  | MSD |  | 6/18/2026 2:56 PM |  |
|  | 3DX Max | | | | | | | | | | | | | | | |  |
|  |  | | | | | | | | | | | | | | | |  |
|  | | | | | | | | | | | | 2 |  | MSD |  | 6/18/2026 2:56 PM |  |
|  | SketchUp | | | | | | | | | | | | | | | |  |
|  |  | | | | | | | | | | | | | | | |  |
| Formatted Reports\\Coding Summary by Code Formatted Report | | | | | | | | | | Page 5 of 36 | | | | | | | |
| 6/18/2026 4:13 PM | | | | | | | | | | | | | | | | | |
|  | | | **Aggregate** |  | **Classification** |  | **Coverage** |  | **Number Of Coding References** | |  | **Reference Number** |  | **Coded By Initials** |  | **Modified On** |  |
|  | | | | | | | | | | | | | | | | | |
|  | | | | | | | | | | | | 3 |  | MSD |  | 6/18/2026 2:56 PM |  |
|  | V-Ray | | | | | | | | | | | | | | | |  |
|  |  | | | | | | | | | | | | | | | |  |
|  | | | | | | | | | | | | 4 |  | MSD |  | 6/18/2026 2:56 PM |  |
|  | Corona | | | | | | | | | | | | | | | |  |
|  |  | | | | | | | | | | | | | | | |  |
|  | | | | | | | | | | | | 5 |  | MSD |  | 6/18/2026 2:56 PM |  |
|  | 5D renderer | | | | | | | | | | | | | | | |  |
|  |  | | | | | | | | | | | | | | | |  |
|  | | | | | | | | | | | | 6 |  | MSD |  | 6/18/2026 2:56 PM |  |
|  | Adobe Suite | | | | | | | | | | | | | | | |  |
|  |  | | | | | | | | | | | | | | | |  |
|  | | | | | | | | | | | | 7 |  | MSD |  | 6/18/2026 2:56 PM |  |
|  | Illustrator | | | | | | | | | | | | | | | |  |
|  |  | | | | | | | | | | | | | | | |  |
|  | | | | | | | | | | | | 8 |  | MSD |  | 6/18/2026 2:56 PM |  |
|  | Photoshop | | | | | | | | | | | | | | | |  |
|  |  | | | | | | | | | | | | | | | |  |
|  | | | | | | | | | | | | 9 |  | MSD |  | 6/18/2026 2:56 PM |  |
|  | Canva | | | | | | | | | | | | | | | |  |
|  |  | | | | | | | | | | | | | | | |  |
|  | | | | | | | | | | | | 10 |  | MSD |  | 6/18/2026 2:56 PM |  |
|  | So I prefer a meeting or maybe even, professional email to be sent, because that way we are both on the safe side, and we can just say, this is what I was given. | | | | | | | | | | | | | | | |  |
|  |  | | | | | | | | | | | | | | | |  |
|  | | | **Files\\Participant 5** | | | | | | | | | | | | | |  |
|  |  |  | No |  |  |  | 0.0255 |  | 4 | |  | | | | | |  |
|  | | |  |  |  |  |  |  |  | |  | | | | | | |
|  | | | | | | | | | | | | 1 |  | MSD |  | 6/18/2026 2:56 PM |  |
|  | I'd say more likely it's it is my communication skills | | | | | | | | | | | | | | | |  |
|  |  | | | | | | | | | | | | | | | |  |
|  | | | | | | | | | | | | 2 |  | MSD |  | 6/18/2026 2:56 PM |  |
|  | So I'd say that's like one of my main things because people have like been genuinely surprised that I take so little time to respond when to me, it just seems like a given, because when it comes to money, I be like, yeah, so I did. That is my main skill from like what? That from other people. Yeah. | | | | | | | | | | | | | | | |  |
|  |  | | | | | | | | | | | | | | | |  |
|  | | | | | | | | | | | | 3 |  | S |  | 6/18/2026 3:07 PM |  |
|  | I think I will be able to take up more kinds of gigs. I'm very into video editing and all that stuff, but I feel like I'm not pretty proficient in that. But I feel like if I did, I can. So in that sense, yes, I feel like I would be able to take up, like more types of stuff. | | | | | | | | | | | | | | | |  |
|  |  | | | | | | | | | | | | | | | |  |
|  | | | | | | | | | | | | 4 |  | S |  | 6/18/2026 3:08 PM |  |
|  | a lot of, like the shortcuts and stuff I use up my muscle memory. So like, if it makes my work a lot faster because I have to think I can just go on my brain, I can go. | | | | | | | | | | | | | | | |  |
|  |  | | | | | | | | | | | | | | | |  |
|  | | | | | | | | | | | | | | | | | |
| Formatted Reports\\Coding Summary by Code Formatted Report | | | | | | | | | | Page 6 of 36 | | | | | | | |
| 6/18/2026 4:13 PM | | | | | | | | | | | | | | | | | |
|  | | | **Aggregate** |  | **Classification** |  | **Coverage** |  | **Number Of Coding References** | |  | **Reference Number** |  | **Coded By Initials** |  | **Modified On** |  |
|  | | | **Files\\Participant 6** | | | | | | | | | | | | | |  |
|  |  |  | No |  |  |  | 0.0077 |  | 5 | |  | | | | | |  |
|  | | |  |  |  |  |  |  |  | |  | | | | | | |
|  | | | | | | | | | | | | 1 |  | MSD |  | 6/18/2026 2:56 PM |  |
|  | video editing | | | | | | | | | | | | | | | |  |
|  |  | | | | | | | | | | | | | | | |  |
|  | | | | | | | | | | | | 2 |  | MSD |  | 6/18/2026 2:56 PM |  |
|  | audio editing | | | | | | | | | | | | | | | |  |
|  |  | | | | | | | | | | | | | | | |  |
|  | | | | | | | | | | | | 3 |  | MSD |  | 6/18/2026 2:56 PM |  |
|  | Canva | | | | | | | | | | | | | | | |  |
|  |  | | | | | | | | | | | | | | | |  |
|  | | | | | | | | | | | | 4 |  | MSD |  | 6/18/2026 2:56 PM |  |
|  | Adobe Premiere Pro | | | | | | | | | | | | | | | |  |
|  |  | | | | | | | | | | | | | | | |  |
|  | | | | | | | | | | | | 5 |  | MSD |  | 6/18/2026 2:56 PM |  |
|  | So you have to be really patient when you're working with these types of clients. At the same time, they're like the type of ones who like, sometimes they'll, like, always come back to you as well because of, like, the work you've done. | | | | | | | | | | | | | | | |  |
|  |  | | | | | | | | | | | | | | | |  |
|  | | | **Files\\Participant 7** | | | | | | | | | | | | | |  |
|  |  |  | No |  |  |  | 0.0149 |  | 1 | |  | | | | | |  |
|  | | |  |  |  |  |  |  |  | |  | | | | | | |
|  | | | | | | | | | | | | 1 |  | S |  | 6/18/2026 3:09 PM |  |
|  | With my with my past experience, if I have a similar project, I know, you know, it's easy for me to get this done with this time because I have a specific idea, because I have done that project, a similar project or similar, what do you call, similar tool, like, if I have done that project already with my, in my past work, obviously, I know how long it would take me to do this thing. | | | | | | | | | | | | | | | |  |
|  |  | | | | | | | | | | | | | | | |  |
|  | | | **Files\\Participant 7** | | | | | | | | | | | | | |  |
|  |  |  | No |  |  |  | 0.0377 |  | 9 | |  | | | | | |  |
|  | | |  |  |  |  |  |  |  | |  | | | | | | |
|  | | | | | | | | | | | | 1 |  | MSD |  | 6/18/2026 2:56 PM |  |
|  | frontend developing | | | | | | | | | | | | | | | |  |
|  |  | | | | | | | | | | | | | | | |  |
|  | | | | | | | | | | | | 2 |  | MSD |  | 6/18/2026 2:56 PM |  |
|  | UI/UX development | | | | | | | | | | | | | | | |  |
|  |  | | | | | | | | | | | | | | | |  |
|  | | | | | | | | | | | | 3 |  | MSD |  | 6/18/2026 2:56 PM |  |
|  | WordPress | | | | | | | | | | | | | | | |  |
|  |  | | | | | | | | | | | | | | | |  |
|  | | | | | | | | | | | | 4 |  | MSD |  | 6/18/2026 2:56 PM |  |
|  | React.js | | | | | | | | | | | | | | | |  |
|  |  | | | | | | | | | | | | | | | |  |
|  | | | | | | | | | | | | 5 |  | MSD |  | 6/18/2026 2:56 PM |  |
|  | Nod.js | | | | | | | | | | | | | | | |  |
|  |  | | | | | | | | | | | | | | | |  |
| Formatted Reports\\Coding Summary by Code Formatted Report | | | | | | | | | | Page 7 of 36 | | | | | | | |
| 6/18/2026 4:13 PM | | | | | | | | | | | | | | | | | |
|  | | | **Aggregate** |  | **Classification** |  | **Coverage** |  | **Number Of Coding References** | |  | **Reference Number** |  | **Coded By Initials** |  | **Modified On** |  |
|  | | | | | | | | | | | | | | | | | |
|  | | | | | | | | | | | | 6 |  | MSD |  | 6/18/2026 2:56 PM |  |
|  | MongoDB | | | | | | | | | | | | | | | |  |
|  |  | | | | | | | | | | | | | | | |  |
|  | | | | | | | | | | | | 7 |  | MSD |  | 6/18/2026 2:56 PM |  |
|  | Yeah, definitely. I think, you know, understanding the client requirement may not be enough in my type of work. So if the requirement is clear, it's easy for me to finish the work soon so that, you know we get less changes. So, yeah, good communication does make a difference. | | | | | | | | | | | | | | | |  |
|  |  | | | | | | | | | | | | | | | |  |
|  | | | | | | | | | | | | 8 |  | MSD |  | 6/18/2026 2:56 PM |  |
|  | Technically doing the task. All right, so, sometimes, you know, when it comes to development of certain features, you know, by by testing and everything it will usually take time to, do a certain, to, code certain features. But sometimes the client wouldn't have that understanding, and they would want to see the, the feature implemented, like right away in a day or two. | | | | | | | | | | | | | | | |  |
|  |  | | | | | | | | | | | | | | | |  |
|  | | | | | | | | | | | | 9 |  | MSD |  | 6/18/2026 2:56 PM |  |
|  | So, I think, obviously I came across certain challenges, but, I don't remember specific to tell you all the challenges, but, every time there is some challenge, you know, I just Google it or YouTube, I think it's very easy to find, resources to, resolve these issues. | | | | | | | | | | | | | | | |  |
|  |  | | | | | | | | | | | | | | | |  |
|  | | | **Files\\Participant 8** | | | | | | | | | | | | | |  |
|  |  |  | No |  |  |  | 0.0109 |  | 1 | |  | | | | | |  |
|  | | |  |  |  |  |  |  |  | |  | | | | | | |
|  | | | | | | | | | | | | 1 |  | S |  | 6/18/2026 3:10 PM |  |
|  | sometimes I feel like when it comes to video editing, I usually take like, a lot of time, and sometimes I wonder whether it is because I don't know so much or something, but I feel like if I learn more, I feel like it will be easier and faster for me to do get stuff done. | | | | | | | | | | | | | | | |  |
|  |  | | | | | | | | | | | | | | | |  |
|  | | | **Files\\Participant 8** | | | | | | | | | | | | | |  |
|  |  |  | No |  |  |  | 0.0016 |  | 4 | |  | | | | | |  |
|  | | |  |  |  |  |  |  |  | |  | | | | | | |
|  | | | | | | | | | | | | 1 |  | MSD |  | 6/18/2026 2:56 PM |  |
|  | video editing | | | | | | | | | | | | | | | |  |
|  |  | | | | | | | | | | | | | | | |  |
|  | | | | | | | | | | | | 2 |  | MSD |  | 6/18/2026 2:56 PM |  |
|  | graphic designing. | | | | | | | | | | | | | | | |  |
|  |  | | | | | | | | | | | | | | | |  |
|  | | | | | | | | | | | | 3 |  | MSD |  | 6/18/2026 2:56 PM |  |
|  | Capcut | | | | | | | | | | | | | | | |  |
|  |  | | | | | | | | | | | | | | | |  |
|  | | | | | | | | | | | | 4 |  | MSD |  | 6/18/2026 2:56 PM |  |
|  | Canva | | | | | | | | | | | | | | | |  |
|  |  | | | | | | | | | | | | | | | |  |
|  | | | **Files\\Participant 9** | | | | | | | | | | | | | |  |
|  |  |  | No |  |  |  | 0.0030 |  | 4 | |  | | | | | |  |
|  | | |  |  |  |  |  |  |  | |  | | | | | | |
|  | | | | | | | | | | | | 1 |  | MSD |  | 6/18/2026 2:56 PM |  |
|  | color gradinglike | | | | | | | | | | | | | | | |  |
|  |  | | | | | | | | | | | | | | | |  |
| Formatted Reports\\Coding Summary by Code Formatted Report | | | | | | | | | | Page 8 of 36 | | | | | | | |
| 6/18/2026 4:13 PM | | | | | | | | | | | | | | | | | |
|  | | | **Aggregate** |  | **Classification** |  | **Coverage** |  | **Number Of Coding References** | |  | **Reference Number** |  | **Coded By Initials** |  | **Modified On** |  |
|  | | | | | | | | | | | | | | | | | |
|  | | | | | | | | | | | | 2 |  | MSD |  | 6/18/2026 2:56 PM |  |
|  | photo retouching | | | | | | | | | | | | | | | |  |
|  |  | | | | | | | | | | | | | | | |  |
|  | | | | | | | | | | | | 3 |  | MSD |  | 6/18/2026 2:56 PM |  |
|  | Photoshop | | | | | | | | | | | | | | | |  |
|  |  | | | | | | | | | | | | | | | |  |
|  | | | | | | | | | | | | 4 |  | MSD |  | 6/18/2026 2:56 PM |  |
|  | Lightroom | | | | | | | | | | | | | | | |  |
|  |  | | | | | | | | | | | | | | | |  |
|  | **Codes\\Engagement** | | | | | | | | | | | | | | | |  |
|  | | **Document** | | | | | | | | | | | | | | |  |
|  | | | **Files\\Participant 1** | | | | | | | | | | | | | |  |
|  |  |  | No |  |  |  | 0.0104 |  | 1 | |  | | | | | |  |
|  | | |  |  |  |  |  |  |  | |  | | | | | | |
|  | | | | | | | | | | | | 1 |  | S |  | 6/18/2026 4:10 PM |  |
|  | I'm looking for work, I guess it's easier for me to look for work now because I use the platforms more. Whereas before I would struggle to find work sometimes early on. But now it's like, like I kind of know where to go kind of thing. | | | | | | | | | | | | | | | |  |
|  |  | | | | | | | | | | | | | | | |  |
|  | | | **Files\\Participant 1** | | | | | | | | | | | | | |  |
|  |  |  | No |  |  |  | 0.0143 |  | 3 | |  | | | | | |  |
|  | | |  |  |  |  |  |  |  | |  | | | | | | |
|  | | | | | | | | | | | | 1 |  | MSD |  | 6/18/2026 2:55 PM |  |
|  | Part-time I would say. | | | | | | | | | | | | | | | |  |
|  |  | | | | | | | | | | | | | | | |  |
|  | | | | | | | | | | | | 2 |  | MSD |  | 6/18/2026 2:55 PM |  |
|  | It kind of depends. So it's like, if I'm looking for work, let's say I get about 2 or 3 commissions sometimes, and then that if I'm working on those, I spend about, like roughly about two hours a day and I get it done like within a week. | | | | | | | | | | | | | | | |  |
|  |  | | | | | | | | | | | | | | | |  |
|  | | | | | | | | | | | | 3 |  | MSD |  | 6/18/2026 2:55 PM |  |
|  | I wouldn't say I'm too committed to the part time freelance. | | | | | | | | | | | | | | | |  |
|  |  | | | | | | | | | | | | | | | |  |
|  | | | **Files\\Participant 10** | | | | | | | | | | | | | |  |
|  |  |  | No |  |  |  | 0.0116 |  | 4 | |  | | | | | |  |
|  | | |  |  |  |  |  |  |  | |  | | | | | | |
|  | | | | | | | | | | | | 1 |  | MSD |  | 6/18/2026 2:55 PM |  |
|  | four to five hours a day. | | | | | | | | | | | | | | | |  |
|  |  | | | | | | | | | | | | | | | |  |
| Formatted Reports\\Coding Summary by Code Formatted Report | | | | | | | | | | Page 9 of 36 | | | | | | | |
| 6/18/2026 4:13 PM | | | | | | | | | | | | | | | | | |
|  | | | **Aggregate** |  | **Classification** |  | **Coverage** |  | **Number Of Coding References** | |  | **Reference Number** |  | **Coded By Initials** |  | **Modified On** |  |
|  | | | | | | | | | | | | | | | | | |
|  | | | | | | | | | | | | 2 |  | MSD |  | 6/18/2026 2:55 PM |  |
|  | around two to three | | | | | | | | | | | | | | | |  |
|  |  | | | | | | | | | | | | | | | |  |
|  | | | | | | | | | | | | 3 |  | MSD |  | 6/18/2026 2:55 PM |  |
|  | I prefer online. | | | | | | | | | | | | | | | |  |
|  |  | | | | | | | | | | | | | | | |  |
|  | | | | | | | | | | | | 4 |  | MSD |  | 6/18/2026 2:55 PM |  |
|  | Not really. I mean, Fiverr was always user friendly platform for freelancers. So personally, I haven't faced any issues. | | | | | | | | | | | | | | | |  |
|  |  | | | | | | | | | | | | | | | |  |
|  | | | **Files\\Participant 11** | | | | | | | | | | | | | |  |
|  |  |  | No |  |  |  | 0.0222 |  | 5 | |  | | | | | |  |
|  | | |  |  |  |  |  |  |  | |  | | | | | | |
|  | | | | | | | | | | | | 1 |  | MSD |  | 6/18/2026 2:55 PM |  |
|  | 2 to 2 and half years. | | | | | | | | | | | | | | | |  |
|  |  | | | | | | | | | | | | | | | |  |
|  | | | | | | | | | | | | 2 |  | MSD |  | 6/18/2026 2:55 PM |  |
|  | I did part time | | | | | | | | | | | | | | | |  |
|  |  | | | | | | | | | | | | | | | |  |
|  | | | | | | | | | | | | 3 |  | MSD |  | 6/18/2026 2:55 PM |  |
|  | 3 to 4 hours per day | | | | | | | | | | | | | | | |  |
|  |  | | | | | | | | | | | | | | | |  |
|  | | | | | | | | | | | | 4 |  | MSD |  | 6/18/2026 2:55 PM |  |
|  | per a week I completed. like 5 to 6 jobs | | | | | | | | | | | | | | | |  |
|  |  | | | | | | | | | | | | | | | |  |
|  | | | | | | | | | | | | 5 |  | MSD |  | 6/18/2026 2:55 PM |  |
|  | i prefer mostly online freelancing. So there are some reasons that I, that I prefer, for online freelancing. Right. So we can set our own schedules, work from anywhere, and we can take breaks when we need. Right. So another thing is that I prefer. So you are the manager. So you have, freedom to, take decisions. Right. So, other than that, that, that those are the those are the things that I consider, | | | | | | | | | | | | | | | |  |
|  |  | | | | | | | | | | | | | | | |  |
|  | | | **Files\\Participant 12** | | | | | | | | | | | | | |  |
|  |  |  | No |  |  |  | 0.0446 |  | 7 | |  | | | | | |  |
|  | | |  |  |  |  |  |  |  | |  | | | | | | |
|  | | | | | | | | | | | | 1 |  | MSD |  | 6/18/2026 2:55 PM |  |
|  | 2 to 3 years | | | | | | | | | | | | | | | |  |
|  |  | | | | | | | | | | | | | | | |  |
|  | | | | | | | | | | | | 2 |  | MSD |  | 6/18/2026 2:55 PM |  |
|  | just a part time freelancer | | | | | | | | | | | | | | | |  |
|  |  | | | | | | | | | | | | | | | |  |
|  | | | | | | | | | | | | 3 |  | MSD |  | 6/18/2026 2:55 PM |  |
|  | 5 to 6 hours per week | | | | | | | | | | | | | | | |  |
|  |  | | | | | | | | | | | | | | | |  |
|  | | | | | | | | | | | | 4 |  | MSD |  | 6/18/2026 2:55 PM |  |
|  | 5 to 6 per week. | | | | | | | | | | | | | | | |  |
|  |  | | | | | | | | | | | | | | | |  |
| Formatted Reports\\Coding Summary by Code Formatted Report | | | | | | | | | | Page 10 of 36 | | | | | | | |
| 6/18/2026 4:13 PM | | | | | | | | | | | | | | | | | |
|  | | | **Aggregate** |  | **Classification** |  | **Coverage** |  | **Number Of Coding References** | |  | **Reference Number** |  | **Coded By Initials** |  | **Modified On** |  |
|  | | | | | | | | | | | | | | | | | |
|  | | | | | | | | | | | | 5 |  | MSD |  | 6/18/2026 2:55 PM |  |
|  | Since this is a online platform and since we are not working with the, like directly with humans, like, like we don't have a direct communication with, with the client. It's, because it's basically text based. There were like some times that, misunderstandings with clients and they might, flag us. So, I have, like, faced certain certain issues like that. | | | | | | | | | | | | | | | |  |
|  |  | | | | | | | | | | | | | | | |  |
|  | | | | | | | | | | | | 6 |  | MSD |  | 6/18/2026 2:55 PM |  |
|  | Then when it goes as a report, and when, Fiverr reaches out to me about it, temporarily, the account gets hold. So during that time period, I don't have any, opportunity to work on any clients because the account will be on hold. So those were like few technical difficulties I have faced in the past. | | | | | | | | | | | | | | | |  |
|  |  | | | | | | | | | | | | | | | |  |
|  | | | | | | | | | | | | 7 |  | MSD |  | 6/18/2026 2:55 PM |  |
|  | Fiverr is open platform for everyone. So there are chances where people misuse those certain platforms. So they keep on updating their policies. | | | | | | | | | | | | | | | |  |
|  |  | | | | | | | | | | | | | | | |  |
|  | | | **Files\\Participant 13** | | | | | | | | | | | | | |  |
|  |  |  | No |  |  |  | 0.0206 |  | 6 | |  | | | | | |  |
|  | | |  |  |  |  |  |  |  | |  | | | | | | |
|  | | | | | | | | | | | | 1 |  | MSD |  | 6/18/2026 2:55 PM |  |
|  | For about two, three years | | | | | | | | | | | | | | | |  |
|  |  | | | | | | | | | | | | | | | |  |
|  | | | | | | | | | | | | 2 |  | MSD |  | 6/18/2026 2:55 PM |  |
|  | part time freelancing | | | | | | | | | | | | | | | |  |
|  |  | | | | | | | | | | | | | | | |  |
|  | | | | | | | | | | | | 3 |  | MSD |  | 6/18/2026 2:55 PM |  |
|  | I dedicate about three hours a day | | | | | | | | | | | | | | | |  |
|  |  | | | | | | | | | | | | | | | |  |
|  | | | | | | | | | | | | 4 |  | MSD |  | 6/18/2026 2:55 PM |  |
|  | So I would typically do about 3 to 4 videos a week | | | | | | | | | | | | | | | |  |
|  |  | | | | | | | | | | | | | | | |  |
|  | | | | | | | | | | | | 5 |  | MSD |  | 6/18/2026 2:55 PM |  |
|  | Online freelancing | | | | | | | | | | | | | | | |  |
|  |  | | | | | | | | | | | | | | | |  |
|  | | | | | | | | | | | | 6 |  | MSD |  | 6/18/2026 2:55 PM |  |
|  | Not not not that I, not that I have noticed. I'm pretty, I'm pretty. Like, I'm pretty okay with, like, apps being a little buggy. So, I, I don't really have a really noticed any problems. No, not from my end. | | | | | | | | | | | | | | | |  |
|  |  | | | | | | | | | | | | | | | |  |
|  | | | **Files\\Participant 14** | | | | | | | | | | | | | |  |
|  |  |  | No |  |  |  | 0.0112 |  | 5 | |  | | | | | |  |
|  | | |  |  |  |  |  |  |  | |  | | | | | | |
|  | | | | | | | | | | | | 1 |  | MSD |  | 6/18/2026 2:55 PM |  |
|  | Around two years | | | | | | | | | | | | | | | |  |
|  |  | | | | | | | | | | | | | | | |  |
|  | | | | | | | | | | | | 2 |  | MSD |  | 6/18/2026 2:55 PM |  |
|  | Part time. | | | | | | | | | | | | | | | |  |
|  |  | | | | | | | | | | | | | | | |  |
|  | | | | | | | | | | | | 3 |  | MSD |  | 6/18/2026 2:55 PM |  |
|  | So once I get a project, I give them a timeline like 10 to 15 days or 15 days later I will finish it off within this period | | | | | | | | | | | | | | | |  |
|  |  | | | | | | | | | | | | | | | |  |
| Formatted Reports\\Coding Summary by Code Formatted Report | | | | | | | | | | Page 11 of 36 | | | | | | | |
| 6/18/2026 4:13 PM | | | | | | | | | | | | | | | | | |
|  | | | **Aggregate** |  | **Classification** |  | **Coverage** |  | **Number Of Coding References** | |  | **Reference Number** |  | **Coded By Initials** |  | **Modified On** |  |
|  | | | | | | | | | | | | | | | | | |
|  | | | | | | | | | | | | 4 |  | MSD |  | 6/18/2026 2:55 PM |  |
|  | A month average I do around 3 to 5 projects. | | | | | | | | | | | | | | | |  |
|  |  | | | | | | | | | | | | | | | |  |
|  | | | | | | | | | | | | 5 |  | MSD |  | 6/18/2026 2:55 PM |  |
|  | Nothing so far. | | | | | | | | | | | | | | | |  |
|  |  | | | | | | | | | | | | | | | |  |
|  | | | **Files\\Participant 2** | | | | | | | | | | | | | |  |
|  |  |  | No |  |  |  | 0.0256 |  | 5 | |  | | | | | |  |
|  | | |  |  |  |  |  |  |  | |  | | | | | | |
|  | | | | | | | | | | | | 1 |  | MSD |  | 6/18/2026 2:55 PM |  |
|  | As a part time. But I used to do it as a full time. | | | | | | | | | | | | | | | |  |
|  |  | | | | | | | | | | | | | | | |  |
|  | | | | | | | | | | | | 2 |  | MSD |  | 6/18/2026 2:55 PM |  |
|  | Four hours, approximately. | | | | | | | | | | | | | | | |  |
|  |  | | | | | | | | | | | | | | | |  |
|  | | | | | | | | | | | | 3 |  | MSD |  | 6/18/2026 2:55 PM |  |
|  | It's it's definitely more than 100 projects. Or, I don't know, probably more than 100. | | | | | | | | | | | | | | | |  |
|  |  | | | | | | | | | | | | | | | |  |
|  | | | | | | | | | | | | 4 |  | S |  | 6/18/2026 4:05 PM |  |
|  | It's it's definitely more than 100 projects. Or, I don't know, probably more than 100. | | | | | | | | | | | | | | | |  |
|  |  | | | | | | | | | | | | | | | |  |
|  | | | | | | | | | | | | 5 |  | MSD |  | 6/18/2026 2:55 PM |  |
|  | Committed in the sense of. I'm pretty disciplined about my my overall time schedule. So once I get to home, I'll start drawing, drawing on whatever the work I have. I'll just do my to do list. So im deciplined about my tasks, my everyday day to day tasks. So maybe I'm, I'm, I'm quite committed to something, but I add it in my schedule. I'm 100% committed to | | | | | | | | | | | | | | | |  |
|  |  | | | | | | | | | | | | | | | |  |
|  | | | **Files\\Participant 3** | | | | | | | | | | | | | |  |
|  |  |  | No |  |  |  | 0.0043 |  | 3 | |  | | | | | |  |
|  | | |  |  |  |  |  |  |  | |  | | | | | | |
|  | | | | | | | | | | | | 1 |  | MSD |  | 6/18/2026 2:55 PM |  |
|  | Eight hours a day | | | | | | | | | | | | | | | |  |
|  |  | | | | | | | | | | | | | | | |  |
|  | | | | | | | | | | | | 2 |  | MSD |  | 6/18/2026 2:55 PM |  |
|  | At least one, 1 or 2 gigs a week, maybe more. It depends on the time. | | | | | | | | | | | | | | | |  |
|  |  | | | | | | | | | | | | | | | |  |
|  | | | | | | | | | | | | 3 |  | MSD |  | 6/18/2026 2:55 PM |  |
|  | So I haven't come across any challenges so far. | | | | | | | | | | | | | | | |  |
|  |  | | | | | | | | | | | | | | | |  |
|  | | | | | | | | | | | | | | | | | |
| Formatted Reports\\Coding Summary by Code Formatted Report | | | | | | | | | | Page 12 of 36 | | | | | | | |
| 6/18/2026 4:13 PM | | | | | | | | | | | | | | | | | |
|  | | | **Aggregate** |  | **Classification** |  | **Coverage** |  | **Number Of Coding References** | |  | **Reference Number** |  | **Coded By Initials** |  | **Modified On** |  |
|  | | | **Files\\Participant 4** | | | | | | | | | | | | | |  |
|  |  |  | No |  |  |  | 0.0143 |  | 5 | |  | | | | | |  |
|  | | |  |  |  |  |  |  |  | |  | | | | | | |
|  | | | | | | | | | | | | 1 |  | MSD |  | 6/18/2026 2:55 PM |  |
|  | Now I've been freelancing for roughly four months | | | | | | | | | | | | | | | |  |
|  |  | | | | | | | | | | | | | | | |  |
|  | | | | | | | | | | | | 2 |  | MSD |  | 6/18/2026 2:55 PM |  |
|  | Full time | | | | | | | | | | | | | | | |  |
|  |  | | | | | | | | | | | | | | | |  |
|  | | | | | | | | | | | | 3 |  | MSD |  | 6/18/2026 2:55 PM |  |
|  | It depends. Since I'm a freelancer, I schedule myself properly. So maybe maximum three, four, five hours a day. Okay. Maximum? Yeah. | | | | | | | | | | | | | | | |  |
|  |  | | | | | | | | | | | | | | | |  |
|  | | | | | | | | | | | | 4 |  | MSD |  | 6/18/2026 2:55 PM |  |
|  | I left my 9-to-5, and I started freelancing full time | | | | | | | | | | | | | | | |  |
|  |  | | | | | | | | | | | | | | | |  |
|  | | | | | | | | | | | | 5 |  | MSD |  | 6/18/2026 2:55 PM |  |
|  | Yeah. I mean, as long as there's no, international level breaching or hacking into my PC, I'm okay. | | | | | | | | | | | | | | | |  |
|  |  | | | | | | | | | | | | | | | |  |
|  | | | **Files\\Participant 5** | | | | | | | | | | | | | |  |
|  |  |  | No |  |  |  | 0.0462 |  | 7 | |  | | | | | |  |
|  | | |  |  |  |  |  |  |  | |  | | | | | | |
|  | | | | | | | | | | | | 1 |  | MSD |  | 6/18/2026 2:55 PM |  |
|  | Right now I'm a full time on. It might change in the future, but that that is what I am. That like I am full time right now. | | | | | | | | | | | | | | | |  |
|  |  | | | | | | | | | | | | | | | |  |
|  | | | | | | | | | | | | 2 |  | MSD |  | 6/18/2026 2:55 PM |  |
|  | That's the thing is, my schedule is very like it really depends. I'm very fluid with my schedule. Like, some days I work from like 10 a.m. to 10 p.m. but other days I just do, like two drawings. And then I spend the rest of the day, you know? So it's like, it really depends. But also I would say I am a little bit of a workaholic, so I do I do over work myself. | | | | | | | | | | | | | | | |  |
|  |  | | | | | | | | | | | | | | | |  |
|  | | | | | | | | | | | | 3 |  | MSD |  | 6/18/2026 2:55 PM |  |
|  | The I'd say about to, 12, 13 ish | | | | | | | | | | | | | | | |  |
|  |  | | | | | | | | | | | | | | | |  |
|  | | | | | | | | | | | | 4 |  | MSD |  | 6/18/2026 2:55 PM |  |
|  | So basically, every time that everything would go off, my, like, my progress would just all get wiped out. So. Yeah. And then just it would go off a lot because I am located in Anuradhapura. So that I guess I guess that was like a huge challenge because it's very annoying to go for the mission back then. | | | | | | | | | | | | | | | |  |
|  |  | | | | | | | | | | | | | | | |  |
|  | | | | | | | | | | | | 5 |  | MSD |  | 6/18/2026 2:55 PM |  |
|  | What I'd say would be like a part time job plus online freelancing. You know, I feel like that would be the best way to go about it for me because, it's like a mix between routine and also freed up | | | | | | | | | | | | | | | |  |
|  |  | | | | | | | | | | | | | | | |  |
|  | | | | | | | | | | | | 6 |  | MSD |  | 6/18/2026 2:55 PM |  |
|  | the more people commission me, the more they'll tell their friends about me, and then they'll tell their friends. It's like a word of mouth thing that that's happening, which, I really, really like, you know, I need that for marketing because it's it's the best kind of marketing, no matter what you do. Like what the best marketing. So I say, that kind of took me to take more gigs. | | | | | | | | | | | | | | | |  |
|  |  | | | | | | | | | | | | | | | |  |
| Formatted Reports\\Coding Summary by Code Formatted Report | | | | | | | | | | Page 13 of 36 | | | | | | | |
| 6/18/2026 4:13 PM | | | | | | | | | | | | | | | | | |
|  | | | **Aggregate** |  | **Classification** |  | **Coverage** |  | **Number Of Coding References** | |  | **Reference Number** |  | **Coded By Initials** |  | **Modified On** |  |
|  | | | | | | | | | | | | | | | | | |
|  | | | | | | | | | | | | 7 |  | MSD |  | 6/18/2026 2:55 PM |  |
|  | there's a huge wall between you two | | | | | | | | | | | | | | | |  |
|  |  | | | | | | | | | | | | | | | |  |
|  | | | **Files\\Participant 6** | | | | | | | | | | | | | |  |
|  |  |  | No |  |  |  | 0.0166 |  | 6 | |  | | | | | |  |
|  | | |  |  |  |  |  |  |  | |  | | | | | | |
|  | | | | | | | | | | | | 1 |  | MSD |  | 6/18/2026 2:55 PM |  |
|  | About, two years now | | | | | | | | | | | | | | | |  |
|  |  | | | | | | | | | | | | | | | |  |
|  | | | | | | | | | | | | 2 |  | MSD |  | 6/18/2026 2:55 PM |  |
|  | part time freelancer | | | | | | | | | | | | | | | |  |
|  |  | | | | | | | | | | | | | | | |  |
|  | | | | | | | | | | | | 3 |  | MSD |  | 6/18/2026 2:55 PM |  |
|  | 4 to 5 hours a day | | | | | | | | | | | | | | | |  |
|  |  | | | | | | | | | | | | | | | |  |
|  | | | | | | | | | | | | 4 |  | MSD |  | 6/18/2026 2:55 PM |  |
|  | There have been times where I've worked on the for ten clients a week, and then sometimes even like the number increases. | | | | | | | | | | | | | | | |  |
|  |  | | | | | | | | | | | | | | | |  |
|  | | | | | | | | | | | | 5 |  | MSD |  | 6/18/2026 2:55 PM |  |
|  | Yeah. There is, sometimes it's just sometimes that the messages don't get follow through properly. You know, like, I guess that's just like, okay, just one of the just technical difficulties that just kind of happens, like a bit of a glitch, like. Yeah. That's that's I'd say that's one thing that's happened, but. Yeah, outside of that, there haven't been much difficulties. | | | | | | | | | | | | | | | |  |
|  |  | | | | | | | | | | | | | | | |  |
|  | | | | | | | | | | | | 6 |  | MSD |  | 6/18/2026 2:55 PM |  |
|  | Sometimes you have days where you just can't find anybody | | | | | | | | | | | | | | | |  |
|  |  | | | | | | | | | | | | | | | |  |
|  | | | **Files\\Participant 7** | | | | | | | | | | | | | |  |
|  |  |  | No |  |  |  | 0.0103 |  | 1 | |  | | | | | |  |
|  | | |  |  |  |  |  |  |  | |  | | | | | | |
|  | | | | | | | | | | | | 1 |  | MSD |  | 6/18/2026 4:01 PM |  |
|  | So, I think, obviously I came across certain challenges, but, I don't remember specific to tell you all the challenges, but, every time there is some challenge, you know, I just Google it or YouTube, I think it's very easy to find, resources to, resolve these issues. | | | | | | | | | | | | | | | |  |
|  |  | | | | | | | | | | | | | | | |  |
|  | | | **Files\\Participant 7** | | | | | | | | | | | | | |  |
|  |  |  | No |  |  |  | 0.0297 |  | 7 | |  | | | | | |  |
|  | | |  |  |  |  |  |  |  | |  | | | | | | |
|  | | | | | | | | | | | | 1 |  | MSD |  | 6/18/2026 2:55 PM |  |
|  | I'm a part time freelancer. | | | | | | | | | | | | | | | |  |
|  |  | | | | | | | | | | | | | | | |  |
|  | | | | | | | | | | | | 2 |  | MSD |  | 6/18/2026 2:55 PM |  |
|  | Yeah. So, if I take, an average, like, two hours a day. | | | | | | | | | | | | | | | |  |
|  |  | | | | | | | | | | | | | | | |  |
| Formatted Reports\\Coding Summary by Code Formatted Report | | | | | | | | | | Page 14 of 36 | | | | | | | |
| 6/18/2026 4:13 PM | | | | | | | | | | | | | | | | | |
|  | | | **Aggregate** |  | **Classification** |  | **Coverage** |  | **Number Of Coding References** | |  | **Reference Number** |  | **Coded By Initials** |  | **Modified On** |  |
|  | | | | | | | | | | | | | | | | | |
|  | | | | | | | | | | | | 3 |  | MSD |  | 6/18/2026 2:55 PM |  |
|  | So if I take an average per month, I would say, about, 3 to 4 projects. | | | | | | | | | | | | | | | |  |
|  |  | | | | | | | | | | | | | | | |  |
|  | | | | | | | | | | | | 4 |  | MSD |  | 6/18/2026 2:55 PM |  |
|  | would say with flexibility and everything, I pick online freelancing. | | | | | | | | | | | | | | | |  |
|  |  | | | | | | | | | | | | | | | |  |
|  | | | | | | | | | | | | 5 |  | MSD |  | 6/18/2026 2:55 PM |  |
|  | Yeah. So as I mentioned, obviously the flexibility of it. Right. And I am someone who values my time and I think, you know, being stuck in a 9 to 5 job it will limit your ability. Then you'll have to, you know, have to, you know, stay in a certain environment and implement what is told to you. | | | | | | | | | | | | | | | |  |
|  |  | | | | | | | | | | | | | | | |  |
|  | | | | | | | | | | | | 6 |  | MSD |  | 6/18/2026 2:55 PM |  |
|  | one thing is. Yeah, definitely. Every time I take a new gig, it's like a new, project, a new client. So it's like a new learning experience. And, | | | | | | | | | | | | | | | |  |
|  |  | | | | | | | | | | | | | | | |  |
|  | | | | | | | | | | | | 7 |  | MSD |  | 6/18/2026 2:55 PM |  |
|  | Yeah, definitely. Yeah. I think you know what I mean. So far I haven't come across any major issues. So. Yeah. | | | | | | | | | | | | | | | |  |
|  |  | | | | | | | | | | | | | | | |  |
|  | | | **Files\\Participant 8** | | | | | | | | | | | | | |  |
|  |  |  | No |  |  |  | 0.0270 |  | 5 | |  | | | | | |  |
|  | | |  |  |  |  |  |  |  | |  | | | | | | |
|  | | | | | | | | | | | | 1 |  | MSD |  | 6/18/2026 2:55 PM |  |
|  | around 5 to 6 clients | | | | | | | | | | | | | | | |  |
|  |  | | | | | | | | | | | | | | | |  |
|  | | | | | | | | | | | | 2 |  | MSD |  | 6/18/2026 2:55 PM |  |
|  | one and a half years. | | | | | | | | | | | | | | | |  |
|  |  | | | | | | | | | | | | | | | |  |
|  | | | | | | | | | | | | 3 |  | MSD |  | 6/18/2026 2:55 PM |  |
|  | So usually up to, 8 to 9 hours per day. | | | | | | | | | | | | | | | |  |
|  |  | | | | | | | | | | | | | | | |  |
|  | | | | | | | | | | | | 4 |  | MSD |  | 6/18/2026 2:55 PM |  |
|  | Usually when it comes to personal connections, that depends on like their requirement I have like, two clients who I work on like monthly basis. So I give them content pieces of like 20 to 30 content pieces per month. When it comes to Fiverr and Upwork around, 8 to 10 per month. | | | | | | | | | | | | | | | |  |
|  |  | | | | | | | | | | | | | | | |  |
|  | | | | | | | | | | | | 5 |  | MSD |  | 6/18/2026 2:55 PM |  |
|  | Well, I do prefer freelancing than a 9 to 5 personally, because I do a lot of other things. So when I'm stuck in a 9 to 5, I have to beg the management for like leaves and I don’t have the freedom to, work whenever I want. Right. Which is why I, started freelancing and and which is why I'm trying to stick to it. | | | | | | | | | | | | | | | |  |
|  |  | | | | | | | | | | | | | | | |  |
|  | | | **Files\\Participant 9** | | | | | | | | | | | | | |  |
|  |  |  | No |  |  |  | 0.0586 |  | 6 | |  | | | | | |  |
|  | | |  |  |  |  |  |  |  | |  | | | | | | |
|  | | | | | | | | | | | | 1 |  | MSD |  | 6/18/2026 2:55 PM |  |
|  | I do it part time | | | | | | | | | | | | | | | |  |
|  |  | | | | | | | | | | | | | | | |  |
|  | | | | | | | | | | | | 2 |  | MSD |  | 6/18/2026 2:55 PM |  |
|  | mostly it's one gig per week | | | | | | | | | | | | | | | |  |
|  |  | | | | | | | | | | | | | | | |  |
| Formatted Reports\\Coding Summary by Code Formatted Report | | | | | | | | | | Page 15 of 36 | | | | | | | |
| 6/18/2026 4:13 PM | | | | | | | | | | | | | | | | | |
|  | | | **Aggregate** |  | **Classification** |  | **Coverage** |  | **Number Of Coding References** | |  | **Reference Number** |  | **Coded By Initials** |  | **Modified On** |  |
|  | | | | | | | | | | | | | | | | | |
|  | | | | | | | | | | | | 3 |  | MSD |  | 6/18/2026 2:55 PM |  |
|  | around 3 to 4 hours | | | | | | | | | | | | | | | |  |
|  |  | | | | | | | | | | | | | | | |  |
|  | | | | | | | | | | | | 4 |  | MSD |  | 6/18/2026 2:55 PM |  |
|  | I prefer online freelancing. Because the more labor, the more, the more money I earn. And, since since the money I earn from the, from, freelancing is in dollars. It helps me a lot since especially since, I live in Sri Lanka with the inflation, I get more. I get more value out of dollars, when I'm doing freelancing, other than doing 9 to 5 job, because it only gives me a fixed salary. So I prefer doing freelancing. | | | | | | | | | | | | | | | |  |
|  |  | | | | | | | | | | | | | | | |  |
|  | | | | | | | | | | | | 5 |  | MSD |  | 6/18/2026 2:55 PM |  |
|  | Mostly once a week. If I have more time, I do more than one gig. | | | | | | | | | | | | | | | |  |
|  |  | | | | | | | | | | | | | | | |  |
|  | | | | | | | | | | | | 6 |  | MSD |  | 6/18/2026 4:02 PM |  |
|  | So if it if it's a major update since since I do have a full time, job, I don't have, I don't really have the full, flexibility or, the full freedom to, like, explore this, these tools as, as I said, and, like, a lot. So if it, if it, let's say, if it's a major update, there would be a small time period, which I have to figure it out on my own, to search up and, to know exactly what is happening and how to, and how to handle the situation. | | | | | | | | | | | | | | | |  |
|  |  | | | | | | | | | | | | | | | |  |
|  | **Codes\\Extrinsic Motivation** | | | | | | | | | | | | | | | |  |
|  | | **Document** | | | | | | | | | | | | | | |  |
|  | | | **Files\\Participant 1** | | | | | | | | | | | | | |  |
|  |  |  | No |  |  |  | 0.0151 |  | 2 | |  | | | | | |  |
|  | | |  |  |  |  |  |  |  | |  | | | | | | |
|  | | | | | | | | | | | | 1 |  | MSD |  | 6/17/2026 9:13 PM |  |
|  | for example for clients they will like struggle like, like give a description of what exactly they want and, yeah. But for me, back then, I would find it hard to, like, pinpoint what exactly I need to do. Whereas now it's like, since I've done a lot of work, I can tell, like, oh, okay, this thing that you need that. | | | | | | | | | | | | | | | |  |
|  |  | | | | | | | | | | | | | | | |  |
|  | | | | | | | | | | | | 2 |  | MSD |  | 6/17/2026 9:13 PM |  |
|  | Just needing money | | | | | | | | | | | | | | | |  |
|  |  | | | | | | | | | | | | | | | |  |
|  | | | **Files\\Participant 10** | | | | | | | | | | | | | |  |
|  |  |  | No |  |  |  | 0.0147 |  | 2 | |  | | | | | |  |
|  | | |  |  |  |  |  |  |  | |  | | | | | | |
|  | | | | | | | | | | | | 1 |  | MSD |  | 6/17/2026 9:13 PM |  |
|  | mostly day to day tasks which I need more money. | | | | | | | | | | | | | | | |  |
|  |  | | | | | | | | | | | | | | | |  |
|  | | | | | | | | | | | | 2 |  | MSD |  | 6/17/2026 9:13 PM |  |
|  | Actually, I'm very interested because, I mean, if you do a nine to five job, we do more, I mean the work we do, and if we do the same work on a freelance platform, we can earn More | | | | | | | | | | | | | | | |  |
|  |  | | | | | | | | | | | | | | | |  |
|  | | | | | | | | | | | | | | | | | |
| Formatted Reports\\Coding Summary by Code Formatted Report | | | | | | | | | | Page 16 of 36 | | | | | | | |
| 6/18/2026 4:13 PM | | | | | | | | | | | | | | | | | |
|  | | | **Aggregate** |  | **Classification** |  | **Coverage** |  | **Number Of Coding References** | |  | **Reference Number** |  | **Coded By Initials** |  | **Modified On** |  |
|  | | | **Files\\Participant 11** | | | | | | | | | | | | | |  |
|  |  |  | No |  |  |  | 0.0162 |  | 2 | |  | | | | | |  |
|  | | |  |  |  |  |  |  |  | |  | | | | | | |
|  | | | | | | | | | | | | 1 |  | MSD |  | 6/17/2026 9:13 PM |  |
|  | Basically, the pricing. | | | | | | | | | | | | | | | |  |
|  |  | | | | | | | | | | | | | | | |  |
|  | | | | | | | | | | | | 2 |  | MSD |  | 6/17/2026 9:13 PM |  |
|  | So basically from freelancing we are getting actually so they are paying more for our work in our country some developers are getting less salary. Right. So by freelancing we are getting really valuable pricing for work that we have completed. So the pricing is really, really good. Comparing to the local software industry, industry pricing. | | | | | | | | | | | | | | | |  |
|  |  | | | | | | | | | | | | | | | |  |
|  | | | **Files\\Participant 12** | | | | | | | | | | | | | |  |
|  |  |  | No |  |  |  | 0.0145 |  | 2 | |  | | | | | |  |
|  | | |  |  |  |  |  |  |  | |  | | | | | | |
|  | | | | | | | | | | | | 1 |  | MSD |  | 6/17/2026 9:13 PM |  |
|  | Just for some extra cash. | | | | | | | | | | | | | | | |  |
|  |  | | | | | | | | | | | | | | | |  |
|  | | | | | | | | | | | | 2 |  | MSD |  | 6/17/2026 9:13 PM |  |
|  | Since I have been on a job full time, the payments I receive from freelancing. It has just been just an additional income. So if I, if I work or not, if I work, it's just additional income for me. If I don't, I still meet my needs. So it doesn't make any change. | | | | | | | | | | | | | | | |  |
|  |  | | | | | | | | | | | | | | | |  |
|  | | | **Files\\Participant 13** | | | | | | | | | | | | | |  |
|  |  |  | No |  |  |  | 0.0293 |  | 2 | |  | | | | | |  |
|  | | |  |  |  |  |  |  |  | |  | | | | | | |
|  | | | | | | | | | | | | 1 |  | MSD |  | 6/18/2026 3:48 PM |  |
|  | if it's a mistake or if they if they think it's a mistake in their opinion, then it does kind of affect me. But then at the end of the day, it because it is also a representation of my work. But at the end of the day, I guess the broader truth is that the client will always want what the client wants. | | | | | | | | | | | | | | | |  |
|  |  | | | | | | | | | | | | | | | |  |
|  | | | | | | | | | | | | 2 |  | MSD |  | 6/18/2026 3:36 PM |  |
|  | my earnings do depend on my skill level and, I guess, that that would motivate me to increase my skill levels. The possibility of earning more if I do increase my skill level. So. Yeah, it does motivate me. | | | | | | | | | | | | | | | |  |
|  |  | | | | | | | | | | | | | | | |  |
|  | | | **Files\\Participant 14** | | | | | | | | | | | | | |  |
|  |  |  | No |  |  |  | 0.0211 |  | 3 | |  | | | | | |  |
|  | | |  |  |  |  |  |  |  | |  | | | | | | |
|  | | | | | | | | | | | | 1 |  | MSD |  | 6/17/2026 9:13 PM |  |
|  | Passion and other that that the money that I get after finishing the project. | | | | | | | | | | | | | | | |  |
|  |  | | | | | | | | | | | | | | | |  |
|  | | | | | | | | | | | | 2 |  | MSD |  | 6/18/2026 3:40 PM |  |
|  | So like when whenever like a client gives me like a good feedback. Like I'm very happy with your project. Something like that. It motivates me to do more projects, like get more projects from different people and start new projects. | | | | | | | | | | | | | | | |  |
|  |  | | | | | | | | | | | | | | | |  |
|  | | | | | | | | | | | | 3 |  | MSD |  | 6/17/2026 9:13 PM |  |
|  | Yeah it definitely affects the number of gigs I'm going to take in the future. | | | | | | | | | | | | | | | |  |
|  |  | | | | | | | | | | | | | | | |  |
| Formatted Reports\\Coding Summary by Code Formatted Report | | | | | | | | | | Page 17 of 36 | | | | | | | |
| 6/18/2026 4:13 PM | | | | | | | | | | | | | | | | | |
|  | | | **Aggregate** |  | **Classification** |  | **Coverage** |  | **Number Of Coding References** | |  | **Reference Number** |  | **Coded By Initials** |  | **Modified On** |  |
|  | | | **Files\\Participant 3** | | | | | | | | | | | | | |  |
|  |  |  | No |  |  |  | 0.0088 |  | 2 | |  | | | | | |  |
|  | | |  |  |  |  |  |  |  | |  | | | | | | |
|  | | | | | | | | | | | | 1 |  | MSD |  | 6/17/2026 9:13 PM |  |
|  | Another one would be an income. Because if I don't have a, like a full set job then I need something to do right? So that would be another one. Like some kind of income. | | | | | | | | | | | | | | | |  |
|  |  | | | | | | | | | | | | | | | |  |
|  | | | | | | | | | | | | 2 |  | MSD |  | 6/17/2026 9:13 PM |  |
|  | I mean, the pay, I try not to think about, but if the pay was good definitely I would take more gigs. | | | | | | | | | | | | | | | |  |
|  |  | | | | | | | | | | | | | | | |  |
|  | | | **Files\\Participant 4** | | | | | | | | | | | | | |  |
|  |  |  | No |  |  |  | 0.0601 |  | 14 | |  | | | | | |  |
|  | | |  |  |  |  |  |  |  | |  | | | | | | |
|  | | | | | | | | | | | | 1 |  | S |  | 6/18/2026 3:31 PM |  |
|  | Since I'm a freelancer, I schedule myself properly. So maybe maximum three, four, five hours a day | | | | | | | | | | | | | | | |  |
|  |  | | | | | | | | | | | | | | | |  |
|  | | | | | | | | | | | | 2 |  | S |  | 6/18/2026 3:31 PM |  |
|  | I would be able to obviously give more quality work. | | | | | | | | | | | | | | | |  |
|  |  | | | | | | | | | | | | | | | |  |
|  | | | | | | | | | | | | 3 |  | S |  | 6/18/2026 3:31 PM |  |
|  | don't want to overboard myself with projects because, there's something called burning out. | | | | | | | | | | | | | | | |  |
|  |  | | | | | | | | | | | | | | | |  |
|  | | | | | | | | | | | | 4 |  | S |  | 6/18/2026 3:31 PM |  |
|  | if people do not have the digital making power to give me, an idea to just do this and do that, they can be like, oh, you do what you want, and then I will do something and they will be like, no, it's not what I want. So, yeah, it'll just be beating around the bush all the time. | | | | | | | | | | | | | | | |  |
|  |  | | | | | | | | | | | | | | | |  |
|  | | | | | | | | | | | | 5 |  | S |  | 6/18/2026 3:31 PM |  |
|  | it's quite straightforward. They know what to do. | | | | | | | | | | | | | | | |  |
|  |  | | | | | | | | | | | | | | | |  |
|  | | | | | | | | | | | | 6 |  | S |  | 6/18/2026 3:31 PM |  |
|  | And the others did not have any idea about any digital work, like, you know, Photoshop or InDesign or even, anything like that. So it was quite hard for me to understand what they want, but also they were also more generic, and it was quite hard for me to come up with the final design for them. | | | | | | | | | | | | | | | |  |
|  |  | | | | | | | | | | | | | | | |  |
|  | | | | | | | | | | | | 7 |  | S |  | 6/17/2026 9:13 PM |  |
|  | But then when it comes to traditional clients, they always want to bargain. | | | | | | | | | | | | | | | |  |
|  |  | | | | | | | | | | | | | | | |  |
|  | | | | | | | | | | | | 8 |  | S |  | 6/18/2026 3:31 PM |  |
|  | I say that this is not about money but about skill | | | | | | | | | | | | | | | |  |
|  |  | | | | | | | | | | | | | | | |  |
|  | | | | | | | | | | | | 9 |  | S |  | 6/18/2026 3:31 PM |  |
|  | That's exactly why I left my 9-to-5, and I started freelancing full time | | | | | | | | | | | | | | | |  |
|  |  | | | | | | | | | | | | | | | |  |
|  | | | | | | | | | | | | 10 |  | S |  | 6/18/2026 3:31 PM |  |
|  | I am quite confident with what I know. | | | | | | | | | | | | | | | |  |
|  |  | | | | | | | | | | | | | | | |  |
| Formatted Reports\\Coding Summary by Code Formatted Report | | | | | | | | | | Page 18 of 36 | | | | | | | |
| 6/18/2026 4:13 PM | | | | | | | | | | | | | | | | | |
|  | | | **Aggregate** |  | **Classification** |  | **Coverage** |  | **Number Of Coding References** | |  | **Reference Number** |  | **Coded By Initials** |  | **Modified On** |  |
|  | | | | | | | | | | | | | | | | | |
|  | | | | | | | | | | | | 11 |  | S |  | 6/17/2026 9:13 PM |  |
|  | The client was happy, so I am happy now. | | | | | | | | | | | | | | | |  |
|  |  | | | | | | | | | | | | | | | |  |
|  | | | | | | | | | | | | 12 |  | S |  | 6/18/2026 3:33 PM |  |
|  | So I was getting paid. An amount less than Rs. 100,000 a month and one month I did work freelance work at home for two hours. | | | | | | | | | | | | | | | |  |
|  |  | | | | | | | | | | | | | | | |  |
|  | | | | | | | | | | | | 13 |  | S |  | 6/18/2026 3:33 PM |  |
|  | And I got paid Rs. 150,000. | | | | | | | | | | | | | | | |  |
|  |  | | | | | | | | | | | | | | | |  |
|  | | | | | | | | | | | | 14 |  | S |  | 6/18/2026 3:33 PM |  |
|  | So that was 30 days, nine hour, nine, eight hours a day. And, that was Rs. 70,000 – 80,000. And, this was two hours and Rs. 150,000. | | | | | | | | | | | | | | | |  |
|  |  | | | | | | | | | | | | | | | |  |
|  | | | **Files\\Participant 5** | | | | | | | | | | | | | |  |
|  |  |  | No |  |  |  | 0.0155 |  | 5 | |  | | | | | |  |
|  | | |  |  |  |  |  |  |  | |  | | | | | | |
|  | | | | | | | | | | | | 1 |  | S |  | 6/17/2026 9:13 PM |  |
|  | helped me learn how to price my art for support because I was doing like 3000 and 2000 rupees back then, and now my prices will be higher | | | | | | | | | | | | | | | |  |
|  |  | | | | | | | | | | | | | | | |  |
|  | | | | | | | | | | | | 2 |  | S |  | 6/17/2026 9:13 PM |  |
|  | it's not only the fact that I'm getting more, you know, like income. | | | | | | | | | | | | | | | |  |
|  |  | | | | | | | | | | | | | | | |  |
|  | | | | | | | | | | | | 3 |  | S |  | 6/17/2026 9:13 PM |  |
|  | the more people commission me, the more they'll tell their friends about me, and then they'll tell their friends. | | | | | | | | | | | | | | | |  |
|  |  | | | | | | | | | | | | | | | |  |
|  | | | | | | | | | | | | 4 |  | S |  | 6/17/2026 9:13 PM |  |
|  | I know that no matter what, I'm going to get a certain amount of commission | | | | | | | | | | | | | | | |  |
|  |  | | | | | | | | | | | | | | | |  |
|  | | | | | | | | | | | | 5 |  | S |  | 6/17/2026 9:13 PM |  |
|  | they're friendly. They have like, there is a criticism a lot of the time and they tip a lot | | | | | | | | | | | | | | | |  |
|  |  | | | | | | | | | | | | | | | |  |
|  | | | **Files\\Participant 6** | | | | | | | | | | | | | |  |
|  |  |  | No |  |  |  | 0.0279 |  | 6 | |  | | | | | |  |
|  | | |  |  |  |  |  |  |  | |  | | | | | | |
|  | | | | | | | | | | | | 1 |  | S |  | 6/17/2026 9:13 PM |  |
|  | the praise people have given me as also like, oh, like what clients I've talked about. It has given me more confidence in my abilities as a video editor | | | | | | | | | | | | | | | |  |
|  |  | | | | | | | | | | | | | | | |  |
|  | | | | | | | | | | | | 2 |  | S |  | 6/17/2026 9:13 PM |  |
|  | you can always have good income, especially like online and all of that | | | | | | | | | | | | | | | |  |
|  |  | | | | | | | | | | | | | | | |  |
|  | | | | | | | | | | | | 3 |  | S |  | 6/17/2026 9:13 PM |  |
|  | they'll, like, always come back to you as well because of, like, the work you've done | | | | | | | | | | | | | | | |  |
|  |  | | | | | | | | | | | | | | | |  |
| Formatted Reports\\Coding Summary by Code Formatted Report | | | | | | | | | | Page 19 of 36 | | | | | | | |
| 6/18/2026 4:13 PM | | | | | | | | | | | | | | | | | |
|  | | | **Aggregate** |  | **Classification** |  | **Coverage** |  | **Number Of Coding References** | |  | **Reference Number** |  | **Coded By Initials** |  | **Modified On** |  |
|  | | | | | | | | | | | | | | | | | |
|  | | | | | | | | | | | | 4 |  | S |  | 6/18/2026 3:45 PM |  |
|  | Yeah, just, honestly, sometimes it's just money. This is like, money is like the main motivation. Okay. Yeah. I mean, if you if, like, there's, if there's, like, something I want to purchase for myself, like, okay, I'm going to get that. I need to get the money for this. And that's what, like one hour. That's what I want, like to pick up more gigs | | | | | | | | | | | | | | | |  |
|  |  | | | | | | | | | | | | | | | |  |
|  | | | | | | | | | | | | 5 |  | S |  | 6/17/2026 9:13 PM |  |
|  | If I earn big, I'm gonna earn. I'll be happy to do more. Like. Yeah, I do, I do earn decently well since I've started | | | | | | | | | | | | | | | |  |
|  |  | | | | | | | | | | | | | | | |  |
|  | | | | | | | | | | | | 6 |  | S |  | 6/17/2026 9:13 PM |  |
|  | you would tend to be motivated to, to do a lot more. If you keep collecting, like a large sums of income. And sometimes you can charge, like even more premium prices on the premium packages to other clients as well. And that way you can earn more like. | | | | | | | | | | | | | | | |  |
|  |  | | | | | | | | | | | | | | | |  |
|  | | | **Files\\Participant 7** | | | | | | | | | | | | | |  |
|  |  |  | No |  |  |  | 0.0068 |  | 2 | |  | | | | | |  |
|  | | |  |  |  |  |  |  |  | |  | | | | | | |
|  | | | | | | | | | | | | 1 |  | S |  | 6/17/2026 9:13 PM |  |
|  | I'm seeing, lots of benefit, in working as a freelancer in terms of finance | | | | | | | | | | | | | | | |  |
|  |  | | | | | | | | | | | | | | | |  |
|  | | | | | | | | | | | | 2 |  | S |  | 6/17/2026 9:13 PM |  |
|  | it affect I think my earning, definitely, makes me do more I mean, make it makes me feel, to do more. | | | | | | | | | | | | | | | |  |
|  |  | | | | | | | | | | | | | | | |  |
|  | | | **Files\\Participant 8** | | | | | | | | | | | | | |  |
|  |  |  | No |  |  |  | 0.0197 |  | 3 | |  | | | | | |  |
|  | | |  |  |  |  |  |  |  | |  | | | | | | |
|  | | | | | | | | | | | | 1 |  | S |  | 6/17/2026 9:13 PM |  |
|  | the basic answer would be to earn more obviously. | | | | | | | | | | | | | | | |  |
|  |  | | | | | | | | | | | | | | | |  |
|  | | | | | | | | | | | | 2 |  | S |  | 6/18/2026 3:43 PM |  |
|  | when the feedback is negative, naturally, I feel like. I feel bad about it whenever I like the draft that I sent them. So whenever they want me to like sometimes completely change the whole thing. I feel bad about it and I feel unmotivated sometimes | | | | | | | | | | | | | | | |  |
|  |  | | | | | | | | | | | | | | | |  |
|  | | | | | | | | | | | | 3 |  | S |  | 6/17/2026 9:13 PM |  |
|  | whenever I am paid on time and whenever I am paid the amount that I quoted for the particular work it does motivate me better. And whenever it doesn’t, it is a drawback in terms of motivation. | | | | | | | | | | | | | | | |  |
|  |  | | | | | | | | | | | | | | | |  |
|  | | | **Files\\Participant 9** | | | | | | | | | | | | | |  |
|  |  |  | No |  |  |  | 0.0631 |  | 5 | |  | | | | | |  |
|  | | |  |  |  |  |  |  |  | |  | | | | | | |
|  | | | | | | | | | | | | 1 |  | S |  | 6/18/2026 3:35 PM |  |
|  | I prefer online freelancing. Because the more labor, the more, the more money I earn. And, since since the money I earn from the, from, freelancing is in dollars. It helps me a lot since especially since, I live in Sri Lanka with the inflation, I get more. I get more value out of dollars, when I'm doing freelancing, other than doing 9 to 5 job, because it only gives me a fixed salary. So I prefer doing freelancing. | | | | | | | | | | | | | | | |  |
|  |  | | | | | | | | | | | | | | | |  |
|  | | | | | | | | | | | | 2 |  | S |  | 6/17/2026 9:13 PM |  |
|  | it's, it's the pay I get | | | | | | | | | | | | | | | |  |
|  |  | | | | | | | | | | | | | | | |  |
| Formatted Reports\\Coding Summary by Code Formatted Report | | | | | | | | | | Page 20 of 36 | | | | | | | |
| 6/18/2026 4:13 PM | | | | | | | | | | | | | | | | | |
|  | | | **Aggregate** |  | **Classification** |  | **Coverage** |  | **Number Of Coding References** | |  | **Reference Number** |  | **Coded By Initials** |  | **Modified On** |  |
|  | | | | | | | | | | | | | | | | | |
|  | | | | | | | | | | | | 3 |  | S |  | 6/17/2026 9:13 PM |  |
|  | the the more gigs I do, the more money I earn. So that's a there's a high chance of me doing more work. So that I am I am rewarded with more pay. With a better pay. Then I do a 9 to 5 job. So that's it. The more it actually motivates me to do more. Since I can earn more, yeah, that is what motivates me. | | | | | | | | | | | | | | | |  |
|  |  | | | | | | | | | | | | | | | |  |
|  | | | | | | | | | | | | 4 |  | S |  | 6/17/2026 9:13 PM |  |
|  | it has only been two years since I have started, using this platforms. Which is only a short time. So with time, I will get a better rating in those sites and a better pay and also a better clients, hopefully I get better clients in the future as well. | | | | | | | | | | | | | | | |  |
|  |  | | | | | | | | | | | | | | | |  |
|  | | | | | | | | | | | | 5 |  | S |  | 6/17/2026 9:13 PM |  |
|  | it's pretty easy to do the gigs online and to get paid instantly | | | | | | | | | | | | | | | |  |
|  |  | | | | | | | | | | | | | | | |  |
|  | **Codes\\Intrinsic Motivation** | | | | | | | | | | | | | | | |  |
|  | | **Document** | | | | | | | | | | | | | | |  |
|  | | | **Files\\Participant 1** | | | | | | | | | | | | | |  |
|  |  |  | No |  |  |  | 0.0856 |  | 12 | |  | | | | | |  |
|  | | |  |  |  |  |  |  |  | |  | | | | | | |
|  | | | | | | | | | | | | 1 |  | S |  | 6/17/2026 9:14 PM |  |
|  | communication is a pretty important thing because, you need to be good clients and, like, try to understand, like what exactly they need, like in terms of, like, the character descriptions and things like that. | | | | | | | | | | | | | | | |  |
|  |  | | | | | | | | | | | | | | | |  |
|  | | | | | | | | | | | | 2 |  | S |  | 6/17/2026 9:14 PM |  |
|  | I am also like learning 2D animation, and I'm doing that on the studio. So you get more familiar with that software. | | | | | | | | | | | | | | | |  |
|  |  | | | | | | | | | | | | | | | |  |
|  | | | | | | | | | | | | 3 |  | S |  | 6/17/2026 9:14 PM |  |
|  | learning how to use, like, color theory and stuff and like, and, I guess drawing skills started when I use, digital | | | | | | | | | | | | | | | |  |
|  |  | | | | | | | | | | | | | | | |  |
|  | | | | | | | | | | | | 4 |  | S |  | 6/17/2026 9:14 PM |  |
|  | So sketchbook wont really help me, like, get make my skills better. But it's like me putting in the effort to draw that, right? Like, like the practice would be what it is. So the software is basically just a sketchbook you know. | | | | | | | | | | | | | | | |  |
|  |  | | | | | | | | | | | | | | | |  |
|  | | | | | | | | | | | | 5 |  | S |  | 6/17/2026 9:14 PM |  |
|  | it's just like wanting to get better as an artist I’d say. It's like making my the quality of my drawings better was, a big motivator. | | | | | | | | | | | | | | | |  |
|  |  | | | | | | | | | | | | | | | |  |
|  | | | | | | | | | | | | 6 |  | S |  | 6/17/2026 9:14 PM |  |
|  | right now I am working full time and I do enjoy what I do. So it's like, I wouldn’t really give up on that to go full time for freelancing | | | | | | | | | | | | | | | |  |
|  |  | | | | | | | | | | | | | | | |  |
|  | | | | | | | | | | | | 7 |  | S |  | 6/17/2026 9:14 PM |  |
|  | Not too difficult. I guess I'm more familiar with, using those platforms now, and I've been using it for a while. | | | | | | | | | | | | | | | |  |
|  |  | | | | | | | | | | | | | | | |  |
|  | | | | | | | | | | | | 8 |  | S |  | 6/17/2026 9:14 PM |  |
|  | I guess it's easier for me to look for work now because I use the platforms more. Whereas before I would struggle to find work sometimes early on. But now it's like, like I kind of know where to go kind of thing. If that makes sense. | | | | | | | | | | | | | | | |  |
|  |  | | | | | | | | | | | | | | | |  |
| Formatted Reports\\Coding Summary by Code Formatted Report | | | | | | | | | | Page 21 of 36 | | | | | | | |
| 6/18/2026 4:13 PM | | | | | | | | | | | | | | | | | |
|  | | | **Aggregate** |  | **Classification** |  | **Coverage** |  | **Number Of Coding References** | |  | **Reference Number** |  | **Coded By Initials** |  | **Modified On** |  |
|  | | | | | | | | | | | | | | | | | |
|  | | | | | | | | | | | | 9 |  | S |  | 6/17/2026 9:14 PM |  |
|  | I wouldn't say I'm too committed to the part time freelance. It's like I do it on and off, | | | | | | | | | | | | | | | |  |
|  |  | | | | | | | | | | | | | | | |  |
|  | | | | | | | | | | | | 10 |  | S |  | 6/17/2026 9:14 PM |  |
|  | Personal preference because I do also want to do, like, personal work. And since I need time for that too, I usually just skip out of the commissions and stuff. | | | | | | | | | | | | | | | |  |
|  |  | | | | | | | | | | | | | | | |  |
|  | | | | | | | | | | | | 11 |  | S |  | 6/17/2026 9:14 PM |  |
|  | I mean, just practice I would say. Just using the softwares more and just doing that work over and over, kind of just make you more familiar with it. So yeah, it's just practice. I would say. | | | | | | | | | | | | | | | |  |
|  |  | | | | | | | | | | | | | | | |  |
|  | | | | | | | | | | | | 12 |  | S |  | 6/17/2026 9:14 PM |  |
|  | I've seen a lot of work sometimes where I just can't really do that to, but there's also been a bunch of work that I've seen where it's like, oh, I can like help out with this. | | | | | | | | | | | | | | | |  |
|  |  | | | | | | | | | | | | | | | |  |
|  | | | **Files\\Participant 10** | | | | | | | | | | | | | |  |
|  |  |  | No |  |  |  | 0.1137 |  | 11 | |  | | | | | |  |
|  | | |  |  |  |  |  |  |  | |  | | | | | | |
|  | | | | | | | | | | | | 1 |  | S |  | 6/17/2026 9:14 PM |  |
|  | more the good communication helps us to do the gig more quickly and easily, both because it's clear for the both parties to what they need and what we are going to do. So the clearer the communication gets | | | | | | | | | | | | | | | |  |
|  |  | | | | | | | | | | | | | | | |  |
|  | | | | | | | | | | | | 2 |  | S |  | 6/17/2026 9:14 PM |  |
|  | I got to know and by experience, mostly I noticed that I should do this and that and yeah, with time, I practiced some new skills. | | | | | | | | | | | | | | | |  |
|  |  | | | | | | | | | | | | | | | |  |
|  | | | | | | | | | | | | 3 |  | S |  | 6/18/2026 3:23 PM |  |
|  | The reason is the most it's freedom. Because you share nine, we are starting nine to five. It's like we are living for money, but actually we need money to live. | | | | | | | | | | | | | | | |  |
|  |  | | | | | | | | | | | | | | | |  |
|  | | | | | | | | | | | | 4 |  | S |  | 6/17/2026 9:14 PM |  |
|  | I use, the technicalities that I have. So I look into those, and if I can, if I see that, I can finish the task, then I accept it, | | | | | | | | | | | | | | | |  |
|  |  | | | | | | | | | | | | | | | |  |
|  | | | | | | | | | | | | 5 |  | S |  | 6/17/2026 9:14 PM |  |
|  | In the beginning, it took more time for me to finish the task, but with the growth and improvement and after all the years, so now it's I can finish the task with a lower time the same task. So normally, I do work for four to five hours, and it's at night, mostly. So it's really convenient. | | | | | | | | | | | | | | | |  |
|  |  | | | | | | | | | | | | | | | |  |
|  | | | | | | | | | | | | 6 |  | S |  | 6/17/2026 9:14 PM |  |
|  | the more you get familiar with the application, you will be able to finish the task quickly. And, yeah, that's the thing. So you can save more time. | | | | | | | | | | | | | | | |  |
|  |  | | | | | | | | | | | | | | | |  |
|  | | | | | | | | | | | | 7 |  | S |  | 6/17/2026 9:14 PM |  |
|  | freedom and the ability to work, being able to work anywhere, anytime. Yeah, and yeah, most of that, and mostly the flexibility, | | | | | | | | | | | | | | | |  |
|  |  | | | | | | | | | | | | | | | |  |
|  | | | | | | | | | | | | 8 |  | S |  | 6/17/2026 9:14 PM |  |
|  | Very satisfied, because I choose my own gigs, and I can select what I want to do, even if I was asked to do any tasks | | | | | | | | | | | | | | | |  |
|  |  | | | | | | | | | | | | | | | |  |
|  | | | | | | | | | | | | 9 |  | S |  | 6/17/2026 9:14 PM |  |
|  | You do what you love. So that's what keeps us right. | | | | | | | | | | | | | | | |  |
|  |  | | | | | | | | | | | | | | | |  |
|  | | | | | | | | | | | | 10 |  | S |  | 6/17/2026 9:14 PM |  |
|  | Motivation, mostly it's it's totally depends on the communication, actually. So mostly we do offer number of rounds of reviews, yeah, and so, I mean, if they ask us more, there will be sometimes that we are annoyed, but we had to end what we started. So anyways, we do keep going. | | | | | | | | | | | | | | | |  |
|  |  | | | | | | | | | | | | | | | |  |
| Formatted Reports\\Coding Summary by Code Formatted Report | | | | | | | | | | Page 22 of 36 | | | | | | | |
| 6/18/2026 4:13 PM | | | | | | | | | | | | | | | | | |
|  | | | **Aggregate** |  | **Classification** |  | **Coverage** |  | **Number Of Coding References** | |  | **Reference Number** |  | **Coded By Initials** |  | **Modified On** |  |
|  | | | | | | | | | | | | | | | | | |
|  | | | | | | | | | | | | 11 |  | S |  | 6/17/2026 9:14 PM |  |
|  | I am confident because it acts as a assistant. So it's like dividing our work so it motivates us to go for more tasks. | | | | | | | | | | | | | | | |  |
|  |  | | | | | | | | | | | | | | | |  |
|  | | | **Files\\Participant 11** | | | | | | | | | | | | | |  |
|  |  |  | No |  |  |  | 0.0786 |  | 9 | |  | | | | | |  |
|  | | |  |  |  |  |  |  |  | |  | | | | | | |
|  | | | | | | | | | | | | 1 |  | S |  | 6/17/2026 9:14 PM |  |
|  | I did part time while i doing studies i engage in these freelancing things. As, average time, I only spent, like, 3 to 4 hours per day. It depends. Right. Its on average. | | | | | | | | | | | | | | | |  |
|  |  | | | | | | | | | | | | | | | |  |
|  | | | | | | | | | | | | 2 |  | S |  | 6/17/2026 9:14 PM |  |
|  | I did projects like, mobile apps, mobile application development. So that's more of like a point of sale application. And I also build applications, for, mobile phone shops to sell their products and get customer feedback. And also I participated some, CSR projects also. | | | | | | | | | | | | | | | |  |
|  |  | | | | | | | | | | | | | | | |  |
|  | | | | | | | | | | | | 3 |  | S |  | 6/17/2026 9:14 PM |  |
|  | So adapting to new technologies is a skill for me. And other than that, there were some skills that were really helpful for me, like the time management skills | | | | | | | | | | | | | | | |  |
|  |  | | | | | | | | | | | | | | | |  |
|  | | | | | | | | | | | | 4 |  | S |  | 6/18/2026 3:54 PM |  |
|  | i prefer mostly online freelancing. So there are some reasons that I, that I prefer, for online freelancing. Right. So we can set our own schedules, work from anywhere, and we can take breaks when we need. Right. So another thing is that I prefer. So you are the manager. So you have, freedom to, take decisions. | | | | | | | | | | | | | | | |  |
|  |  | | | | | | | | | | | | | | | |  |
|  | | | | | | | | | | | | 5 |  | S |  | 6/18/2026 3:17 PM |  |
|  | So getting new challenges and doing innovative, innovative projects that, motivates me to do, more freelance. | | | | | | | | | | | | | | | |  |
|  |  | | | | | | | | | | | | | | | |  |
|  | | | | | | | | | | | | 6 |  | S |  | 6/17/2026 9:14 PM |  |
|  | I’m very familiar with JavaScript | | | | | | | | | | | | | | | |  |
|  |  | | | | | | | | | | | | | | | |  |
|  | | | | | | | | | | | | 7 |  | S |  | 6/17/2026 9:14 PM |  |
|  | i have the freedom to take decisions and so some 9-5 jobs are not remote, but we have to but let's be good. So freelancing is a fully remote job. So, that's the main reason I think, main reason for choosing freelancing rather than getting an individual. | | | | | | | | | | | | | | | |  |
|  |  | | | | | | | | | | | | | | | |  |
|  | | | | | | | | | | | | 8 |  | S |  | 6/17/2026 9:14 PM |  |
|  | according To me, that's the most important thing in freelancing that the clients feedback are really helpful to, get more jobs. So not only getting jobs by their feedback, we can, we can learn what are the things that we must improve from, our side as a developer, from ourself. | | | | | | | | | | | | | | | |  |
|  |  | | | | | | | | | | | | | | | |  |
|  | | | | | | | | | | | | 9 |  | S |  | 6/17/2026 9:14 PM |  |
|  | we have to spend our time for exploring new technologies, fully new technologies and the changes that happens in upcoming weeks. So we have to get get update with the, new technologies. | | | | | | | | | | | | | | | |  |
|  |  | | | | | | | | | | | | | | | |  |
|  | | | **Files\\Participant 12** | | | | | | | | | | | | | |  |
|  |  |  | No |  |  |  | 0.1238 |  | 15 | |  | | | | | |  |
|  | | |  |  |  |  |  |  |  | |  | | | | | | |
|  | | | | | | | | | | | | 1 |  | S |  | 6/17/2026 9:14 PM |  |
|  | That's how I got into freelancing. And, what I did was mainly was I was into web development, App development and digital marketing and graphic Designing. | | | | | | | | | | | | | | | |  |
|  |  | | | | | | | | | | | | | | | |  |
|  | | | | | | | | | | | | 2 |  | S |  | 6/17/2026 9:14 PM |  |
|  | digital marketing, I do my self research on my clients. I work on the, strategies I have to implement on them. And I use ChatGPT for research purposes, to make reports to learn about competitors. | | | | | | | | | | | | | | | |  |
|  |  | | | | | | | | | | | | | | | |  |
| Formatted Reports\\Coding Summary by Code Formatted Report | | | | | | | | | | Page 23 of 36 | | | | | | | |
| 6/18/2026 4:13 PM | | | | | | | | | | | | | | | | | |
|  | | | **Aggregate** |  | **Classification** |  | **Coverage** |  | **Number Of Coding References** | |  | **Reference Number** |  | **Coded By Initials** |  | **Modified On** |  |
|  | | | | | | | | | | | | | | | | | |
|  | | | | | | | | | | | | 3 |  | S |  | 6/17/2026 9:14 PM |  |
|  | I use YouTube, to learn about new skills when it comes to digital marketing and, designing. | | | | | | | | | | | | | | | |  |
|  |  | | | | | | | | | | | | | | | |  |
|  | | | | | | | | | | | | 4 |  | S |  | 6/17/2026 9:14 PM |  |
|  | According to my experience, I feel that I can deliver a product with value. | | | | | | | | | | | | | | | |  |
|  |  | | | | | | | | | | | | | | | |  |
|  | | | | | | | | | | | | 5 |  | S |  | 6/17/2026 9:14 PM |  |
|  | I, learned them through YouTube, through friends, through peer groups. It was all most of them was self-learning. | | | | | | | | | | | | | | | |  |
|  |  | | | | | | | | | | | | | | | |  |
|  | | | | | | | | | | | | 6 |  | S |  | 6/17/2026 9:14 PM |  |
|  | More than the communication, I would say the understanding of the client, like how they, present the requirement to me without confusion. | | | | | | | | | | | | | | | |  |
|  |  | | | | | | | | | | | | | | | |  |
|  | | | | | | | | | | | | 7 |  | S |  | 6/17/2026 9:14 PM |  |
|  | So freelancing is not always stable. So for just for a career perspective, I would stick to 9 to 5. But when I have time, I'll make myself available for freelancing as well, just to bounce back. | | | | | | | | | | | | | | | |  |
|  |  | | | | | | | | | | | | | | | |  |
|  | | | | | | | | | | | | 8 |  | S |  | 6/18/2026 3:20 PM |  |
|  | So first of all, when I get a gig requirement. I just see if I can deliver the product within the client's, timeline, and I just check my work schedule as well as my work. So if it if it fits my criteria, my if it fits my schedule, I will definitely take up the gig, Because every gig has something to learn. | | | | | | | | | | | | | | | |  |
|  |  | | | | | | | | | | | | | | | |  |
|  | | | | | | | | | | | | 9 |  | S |  | 6/17/2026 9:14 PM |  |
|  | the choice to decline something I don't like to work with. If it's a job. But if I'm assigned to a task, I have to work on it. | | | | | | | | | | | | | | | |  |
|  |  | | | | | | | | | | | | | | | |  |
|  | | | | | | | | | | | | 10 |  | S |  | 6/17/2026 9:14 PM |  |
|  | I can, work on the things I feel I can generate a better result. | | | | | | | | | | | | | | | |  |
|  |  | | | | | | | | | | | | | | | |  |
|  | | | | | | | | | | | | 11 |  | S |  | 6/17/2026 9:14 PM |  |
|  | when I get a gig, I take up the project only if I can deliver. So when I like, read the requirement, I just picture it on my mind, like how I will be delivering. So, now then I have this mind. So when I, start the project, if I complete the certain mind map, | | | | | | | | | | | | | | | |  |
|  |  | | | | | | | | | | | | | | | |  |
|  | | | | | | | | | | | | 12 |  | S |  | 6/17/2026 9:14 PM |  |
|  | So far from the with the clients that I have worked. I do have a 70% to 80% satisfaction with the work I have done because when it comes to work I do my best. | | | | | | | | | | | | | | | |  |
|  |  | | | | | | | | | | | | | | | |  |
|  | | | | | | | | | | | | 13 |  | S |  | 6/17/2026 9:14 PM |  |
|  | if I get a positive feedback, I anyways, I take it take it seriously | | | | | | | | | | | | | | | |  |
|  |  | | | | | | | | | | | | | | | |  |
|  | | | | | | | | | | | | 14 |  | S |  | 6/17/2026 9:14 PM |  |
|  | if I get a negative feedback with my work, I just first examine the project, what I have done, the product I have delivered, and I just check if their perspective is correct, if the points are correct, and if I see something I can learn from, you know, I take it and I learn it to fix it. | | | | | | | | | | | | | | | |  |
|  |  | | | | | | | | | | | | | | | |  |
|  | | | | | | | | | | | | 15 |  | S |  | 6/17/2026 9:14 PM |  |
|  | If I get constructive criticism, I just take a step back to re-examine everything and, like, if I feel that, there are certain things to learn, I take some time and learn and then go on to my next project. | | | | | | | | | | | | | | | |  |
|  |  | | | | | | | | | | | | | | | |  |
|  | | | | | | | | | | | | | | | | | |
| Formatted Reports\\Coding Summary by Code Formatted Report | | | | | | | | | | Page 24 of 36 | | | | | | | |
| 6/18/2026 4:13 PM | | | | | | | | | | | | | | | | | |
|  | | | **Aggregate** |  | **Classification** |  | **Coverage** |  | **Number Of Coding References** | |  | **Reference Number** |  | **Coded By Initials** |  | **Modified On** |  |
|  | | | **Files\\Participant 13** | | | | | | | | | | | | | |  |
|  |  |  | No |  |  |  | 0.0834 |  | 8 | |  | | | | | |  |
|  | | |  |  |  |  |  |  |  | |  | | | | | | |
|  | | | | | | | | | | | | 1 |  | S |  | 6/17/2026 9:14 PM |  |
|  | I do quite enjoy being an online freelancer because I get to meet quite a few different individuals, get to handle different personalities and traits and whatnot. And it really helps my people skills. And yeah, so I do quite enjoy it. I have been enjoying it. | | | | | | | | | | | | | | | |  |
|  |  | | | | | | | | | | | | | | | |  |
|  | | | | | | | | | | | | 2 |  | S |  | 6/17/2026 9:14 PM |  |
|  | It's easier if like there is clear communication and both parties or all parties involved understand everything about the piece of content or the schedule that is planned and so on and so forth. So clear communication does have that process, you know, happen much faster. | | | | | | | | | | | | | | | |  |
|  |  | | | | | | | | | | | | | | | |  |
|  | | | | | | | | | | | | 3 |  | S |  | 6/17/2026 9:14 PM |  |
|  | online freelancing would allow me to just be able to get gigs from anywhere, whether I'm on the run | | | | | | | | | | | | | | | |  |
|  |  | | | | | | | | | | | | | | | |  |
|  | | | | | | | | | | | | 4 |  | S |  | 6/17/2026 9:14 PM |  |
|  | proper scheduling and time management and, priority setting and stuff like that as well. Yeah, that that's how I really do manage to get all my work done by the end of the week. | | | | | | | | | | | | | | | |  |
|  |  | | | | | | | | | | | | | | | |  |
|  | | | | | | | | | | | | 5 |  | S |  | 6/17/2026 9:14 PM |  |
|  | The freedom and the fact that I can do other things while doing this. And I'm not stuck inside a building or at a desk for an ungodly number of hours. | | | | | | | | | | | | | | | |  |
|  |  | | | | | | | | | | | | | | | |  |
|  | | | | | | | | | | | | 6 |  | S |  | 6/17/2026 9:14 PM |  |
|  | I am an online freelance and not, doing a 9 to 5 job. It does help me balance, like, all my other responsibilities, because I have time to actually have other responsibilities. | | | | | | | | | | | | | | | |  |
|  |  | | | | | | | | | | | | | | | |  |
|  | | | | | | | | | | | | 7 |  | S |  | 6/17/2026 9:14 PM |  |
|  | Positive feedback does motivate me because that clearly that would indicate that I'm, I know what I'm doing. | | | | | | | | | | | | | | | |  |
|  |  | | | | | | | | | | | | | | | |  |
|  | | | | | | | | | | | | 8 |  | S |  | 6/17/2026 9:14 PM |  |
|  | my earnings do depend on my skill level and, I guess, that that would motivate me to increase my skill levels. The possibility of earning more if I do increase my skill level. So. Yeah, it does motivate me. | | | | | | | | | | | | | | | |  |
|  |  | | | | | | | | | | | | | | | |  |
|  | | | **Files\\Participant 14** | | | | | | | | | | | | | |  |
|  |  |  | No |  |  |  | 0.1336 |  | 10 | |  | | | | | |  |
|  | | |  |  |  |  |  |  |  | |  | | | | | | |
|  | | | | | | | | | | | | 1 |  | S |  | 6/17/2026 9:14 PM |  |
|  | since I do all this part time, I only get projects from individuals. I don't do projects for companies. So once I get a project, I give them a timeline like 10 to 15 days or 15 days later I will finish it off within this period. Okay. So based on the project, based on how big is the project, I divide my schedule into parts and complete the project. | | | | | | | | | | | | | | | |  |
|  |  | | | | | | | | | | | | | | | |  |
|  | | | | | | | | | | | | 2 |  | S |  | 6/17/2026 9:14 PM |  |
|  | I think it has helped me to finish those easy and quickly. There were sometimes, what I do at those times were I would set up an extra meeting, then gather all the requirements from the very beginning and just clear out what I, What I was not clear earlier. | | | | | | | | | | | | | | | |  |
|  |  | | | | | | | | | | | | | | | |  |
|  | | | | | | | | | | | | 3 |  | S |  | 6/17/2026 9:14 PM |  |
|  | My interest in doing RPA because I love, building automations for people. | | | | | | | | | | | | | | | |  |
|  |  | | | | | | | | | | | | | | | |  |
|  | | | | | | | | | | | | 4 |  | S |  | 6/18/2026 3:14 PM |  |
|  | Passion and other that that the money that I get after finishing the project. | | | | | | | | | | | | | | | |  |
|  |  | | | | | | | | | | | | | | | |  |
| Formatted Reports\\Coding Summary by Code Formatted Report | | | | | | | | | | Page 25 of 36 | | | | | | | |
| 6/18/2026 4:13 PM | | | | | | | | | | | | | | | | | |
|  | | | **Aggregate** |  | **Classification** |  | **Coverage** |  | **Number Of Coding References** | |  | **Reference Number** |  | **Coded By Initials** |  | **Modified On** |  |
|  | | | | | | | | | | | | | | | | | |
|  | | | | | | | | | | | | 5 |  | S |  | 6/17/2026 9:14 PM |  |
|  | So every single day I would allocate myself for like a 1 or 2 hour time slot to do my freelancing. So likewise, when I get a project I would break down my like if my project time period is ten days, I would break down those ten days into different time slots that I am going to work during these hours. | | | | | | | | | | | | | | | |  |
|  |  | | | | | | | | | | | | | | | |  |
|  | | | | | | | | | | | | 6 |  | S |  | 6/17/2026 9:14 PM |  |
|  | I think it makes me to it makes it easy for me to finish my projects on time. Then it's easy for me to work because I already know what the application is. What are the loopholes that I can use to finish the projects early. Likewise. | | | | | | | | | | | | | | | |  |
|  |  | | | | | | | | | | | | | | | |  |
|  | | | | | | | | | | | | 7 |  | S |  | 6/17/2026 9:14 PM |  |
|  | since I'm still an undergraduate, so I'm doing my university stuff at the same time, you know? Yeah. So it gives me the chance to, like, balance both of them at the same time because I don't have, like, a strict schedule. So whenever I have lectures, I can move on and reschedule my, whatever the work to a different time. Stuff like that. So it gives me the flexibility to balance stuff at the same time. | | | | | | | | | | | | | | | |  |
|  |  | | | | | | | | | | | | | | | |  |
|  | | | | | | | | | | | | 8 |  | S |  | 6/17/2026 9:14 PM |  |
|  | I think I'm getting to work with new people, even. I'm getting. I'm learning new things from those people. I'm doing different projects for different people. And I'm getting to know what their field is, what they're doing, and what their processes are. | | | | | | | | | | | | | | | |  |
|  |  | | | | | | | | | | | | | | | |  |
|  | | | | | | | | | | | | 9 |  | S |  | 6/17/2026 9:14 PM |  |
|  | So like when whenever like a client gives me like a good feedback. Like I'm very happy with your project. Something like that. It motivates me to do more projects, like get more projects from different people and start new projects. | | | | | | | | | | | | | | | |  |
|  |  | | | | | | | | | | | | | | | |  |
|  | | | | | | | | | | | | 10 |  | S |  | 6/17/2026 9:14 PM |  |
|  | I think it's the only way that I can find new clients because I don't like, go to their place and ask them like, do you have any projects? I'm doing freelancing. Can you give it to me? I think internet is the best place to find new clients and new projects. | | | | | | | | | | | | | | | |  |
|  |  | | | | | | | | | | | | | | | |  |
|  | | | **Files\\Participant 2** | | | | | | | | | | | | | |  |
|  |  |  | No |  |  |  | 0.0526 |  | 9 | |  | | | | | |  |
|  | | |  |  |  |  |  |  |  | |  | | | | | | |
|  | | | | | | | | | | | | 1 |  | S |  | 6/17/2026 9:14 PM |  |
|  | it is it is pretty important to have a communication skill when it comes to do freelancing stuff. | | | | | | | | | | | | | | | |  |
|  |  | | | | | | | | | | | | | | | |  |
|  | | | | | | | | | | | | 2 |  | S |  | 6/17/2026 9:14 PM |  |
|  | It's defenitly my art skills. Like, I get more and more of more of my clients based on my portfolios. | | | | | | | | | | | | | | | |  |
|  |  | | | | | | | | | | | | | | | |  |
|  | | | | | | | | | | | | 3 |  | S |  | 6/17/2026 9:14 PM |  |
|  | They gave me an instruction like, which is not really clear. Okay, so I did I an animation, a rough animation as what I thought, would be perfect. So somehow they didn't like it. | | | | | | | | | | | | | | | |  |
|  |  | | | | | | | | | | | | | | | |  |
|  | | | | | | | | | | | | 4 |  | S |  | 6/17/2026 9:14 PM |  |
|  | I'm still learning though even.after six year. | | | | | | | | | | | | | | | |  |
|  |  | | | | | | | | | | | | | | | |  |
|  | | | | | | | | | | | | 5 |  | S |  | 6/17/2026 9:14 PM |  |
|  | Committed in the sense of. I'm pretty disciplined about my my overall time schedule. So once I get to home, I'll start drawing, drawing on whatever the work I have. I'll just do my to do list. So im deciplined about my tasks, my everyday day to day tasks. So maybe I'm, I'm, I'm quite committed to something, but I add it in my schedule. I'm 100% committed to | | | | | | | | | | | | | | | |  |
|  |  | | | | | | | | | | | | | | | |  |
|  | | | | | | | | | | | | 6 |  | S |  | 6/17/2026 9:14 PM |  |
|  | I can reject, project if I don't like it. | | | | | | | | | | | | | | | |  |
|  |  | | | | | | | | | | | | | | | |  |
|  | | | | | | | | | | | | 7 |  | S |  | 6/17/2026 9:14 PM |  |
|  | I used to draw a lot with my iPad and, procreate. It's basically like, like an extension of my myself. So, im so used to it right now, | | | | | | | | | | | | | | | |  |
|  |  | | | | | | | | | | | | | | | |  |
| Formatted Reports\\Coding Summary by Code Formatted Report | | | | | | | | | | Page 26 of 36 | | | | | | | |
| 6/18/2026 4:13 PM | | | | | | | | | | | | | | | | | |
|  | | | **Aggregate** |  | **Classification** |  | **Coverage** |  | **Number Of Coding References** | |  | **Reference Number** |  | **Coded By Initials** |  | **Modified On** |  |
|  | | | | | | | | | | | | | | | | | |
|  | | | | | | | | | | | | 8 |  | S |  | 6/17/2026 9:14 PM |  |
|  | the valuable feedback I got from a client was my communication skills | | | | | | | | | | | | | | | |  |
|  |  | | | | | | | | | | | | | | | |  |
|  | | | | | | | | | | | | 9 |  | S |  | 6/17/2026 9:14 PM |  |
|  | I need to put some time to improve my skill. | | | | | | | | | | | | | | | |  |
|  |  | | | | | | | | | | | | | | | |  |
|  | | | **Files\\Participant 3** | | | | | | | | | | | | | |  |
|  |  |  | No |  |  |  | 0.0852 |  | 16 | |  | | | | | |  |
|  | | |  |  |  |  |  |  |  | |  | | | | | | |
|  | | | | | | | | | | | | 1 |  | S |  | 6/17/2026 9:14 PM |  |
|  | The main skill which we all need, even as a freelancer is the basic communication skills, | | | | | | | | | | | | | | | |  |
|  |  | | | | | | | | | | | | | | | |  |
|  | | | | | | | | | | | | 2 |  | S |  | 6/17/2026 9:14 PM |  |
|  | Right now, just social media content, because that's like a bit easy to time manage especially with the studies. | | | | | | | | | | | | | | | |  |
|  |  | | | | | | | | | | | | | | | |  |
|  | | | | | | | | | | | | 3 |  | S |  | 6/17/2026 9:14 PM |  |
|  | because I also need to keep myself busy. I would take up more like, every opportunity that comes, | | | | | | | | | | | | | | | |  |
|  |  | | | | | | | | | | | | | | | |  |
|  | | | | | | | | | | | | 4 |  | S |  | 6/17/2026 9:14 PM |  |
|  | the creative area of things, because that's a new, side of things, which is still developing and I'm still learning. | | | | | | | | | | | | | | | |  |
|  |  | | | | | | | | | | | | | | | |  |
|  | | | | | | | | | | | | 5 |  | S |  | 6/17/2026 9:14 PM |  |
|  | trying to learn more and more things about like the applications I use, for example in Photoshop. I'm still learning new things every day. So so that's like the main factor. | | | | | | | | | | | | | | | |  |
|  |  | | | | | | | | | | | | | | | |  |
|  | | | | | | | | | | | | 6 |  | S |  | 6/17/2026 9:14 PM |  |
|  | when you get to meet new people because I like to make new contacts and I get to know more people, | | | | | | | | | | | | | | | |  |
|  |  | | | | | | | | | | | | | | | |  |
|  | | | | | | | | | | | | 7 |  | S |  | 6/17/2026 9:14 PM |  |
|  | the more experience you have, the quicker you definitely try to finish it, because youre used to making it over and over, and using the same tools. The same creative mindset, which you apply for those kind of stuff. The posts that it just gets easier over time. | | | | | | | | | | | | | | | |  |
|  |  | | | | | | | | | | | | | | | |  |
|  | | | | | | | | | | | | 8 |  | S |  | 6/17/2026 9:14 PM |  |
|  | So I usually Adobe Suite, Photoshop and all that. And then when it comes to communicating, I use mainly whatsapp or LinkedIn. like in other cases, it depends on the people also. I use Instagram. | | | | | | | | | | | | | | | |  |
|  |  | | | | | | | | | | | | | | | |  |
|  | | | | | | | | | | | | 9 |  | S |  | 6/17/2026 9:14 PM |  |
|  | I know I'm quite familiar with all of them compared to when I first used them. | | | | | | | | | | | | | | | |  |
|  |  | | | | | | | | | | | | | | | |  |
|  | | | | | | | | | | | | 10 |  | S |  | 6/17/2026 9:14 PM |  |
|  | the more you use it to make something the more easy it gets. You will get used to all the controls and stuff. | | | | | | | | | | | | | | | |  |
|  |  | | | | | | | | | | | | | | | |  |
|  | | | | | | | | | | | | 11 |  | S |  | 6/17/2026 9:14 PM |  |
|  | the fact that I can apply my own style of creativity to it because when I did my 9 to 5 jobs, I had to stick to how they wanted or to be done, like their style of work all that. So the whole online gig thing and freelancing and all that, I can apply my thinking and then just create something with my style so that the client will also like understand, | | | | | | | | | | | | | | | |  |
|  |  | | | | | | | | | | | | | | | |  |
| Formatted Reports\\Coding Summary by Code Formatted Report | | | | | | | | | | Page 27 of 36 | | | | | | | |
| 6/18/2026 4:13 PM | | | | | | | | | | | | | | | | | |
|  | | | **Aggregate** |  | **Classification** |  | **Coverage** |  | **Number Of Coding References** | |  | **Reference Number** |  | **Coded By Initials** |  | **Modified On** |  |
|  | | | | | | | | | | | | | | | | | |
|  | | | | | | | | | | | | 12 |  | S |  | 6/17/2026 9:14 PM |  |
|  | It's very important because, because I have to balance my studies. Plus, the work. So that way, the flexibility part will be very important for me, because I don't know when I will be busy with my studies. | | | | | | | | | | | | | | | |  |
|  |  | | | | | | | | | | | | | | | |  |
|  | | | | | | | | | | | | 13 |  | S |  | 6/17/2026 9:14 PM |  |
|  | With the 9 to 5 there is a structure, I had to keep with the company. But when it compares to, like, freelancing, it's like it's like the total opposite, like the structure, its my structure plus the clients like how they want it also but mainly its how I design it. | | | | | | | | | | | | | | | |  |
|  |  | | | | | | | | | | | | | | | |  |
|  | | | | | | | | | | | | 14 |  | S |  | 6/17/2026 9:14 PM |  |
|  | I mean it gets annoying at some point because that feedback can be quite annoying, especially the timing of it. So I guess the timing. But the more feedback I do get, the more like for me. Apart from the annoy, it just also helps me understand how they do things. | | | | | | | | | | | | | | | |  |
|  |  | | | | | | | | | | | | | | | |  |
|  | | | | | | | | | | | | 15 |  | S |  | 6/17/2026 9:14 PM |  |
|  | I can try new things also just to see what makes them happy or like whether it will satisfy them. | | | | | | | | | | | | | | | |  |
|  |  | | | | | | | | | | | | | | | |  |
|  | | | | | | | | | | | | 16 |  | S |  | 6/17/2026 9:14 PM |  |
|  | I am getting more confident on using those tools and especially incorporating into my work. | | | | | | | | | | | | | | | |  |
|  |  | | | | | | | | | | | | | | | |  |
|  | | | **Files\\Participant 4** | | | | | | | | | | | | | |  |
|  |  |  | No |  |  |  | 0.0395 |  | 8 | |  | | | | | |  |
|  | | |  |  |  |  |  |  |  | |  | | | | | | |
|  | | | | | | | | | | | | 1 |  | S |  | 6/18/2026 3:50 PM |  |
|  | Maybe maybe three projects a week because I also don't want, don't want to overboard myself with projects because, there's something called burning out. | | | | | | | | | | | | | | | |  |
|  |  | | | | | | | | | | | | | | | |  |
|  | | | | | | | | | | | | 2 |  | S |  | 6/18/2026 3:56 PM |  |
|  | I would say its not even about money at this point because I had client who wanted to pay me low but give me a lot of work. And I say that this is not about money but about skill, the passion that I have. And if it was about money then I would have gone and worked a 9-5 job. Its not about money | | | | | | | | | | | | | | | |  |
|  |  | | | | | | | | | | | | | | | |  |
|  | | | | | | | | | | | | 3 |  | S |  | 6/17/2026 9:14 PM |  |
|  | I try to improve myself | | | | | | | | | | | | | | | |  |
|  |  | | | | | | | | | | | | | | | |  |
|  | | | | | | | | | | | | 4 |  | S |  | 6/18/2026 3:25 PM |  |
|  | So I was getting paid. An amount less than Rs. 100,000 a month and one month I did work freelance work at home for two hours. | | | | | | | | | | | | | | | |  |
|  |  | | | | | | | | | | | | | | | |  |
|  | | | | | | | | | | | | 5 |  | S |  | 6/18/2026 3:25 PM |  |
|  | And I got paid Rs. 150,000. | | | | | | | | | | | | | | | |  |
|  |  | | | | | | | | | | | | | | | |  |
|  | | | | | | | | | | | | 6 |  | S |  | 6/18/2026 3:25 PM |  |
|  | So that was 30 days, nine hour, nine, eight hours a day. And, that was Rs. 70,000 – 80,000. And, this was two hours and Rs. 150,000. | | | | | | | | | | | | | | | |  |
|  |  | | | | | | | | | | | | | | | |  |
|  | | | | | | | | | | | | 7 |  | S |  | 6/17/2026 9:14 PM |  |
|  | some effort as well. And maybe making more confident that what for your end result. | | | | | | | | | | | | | | | |  |
|  |  | | | | | | | | | | | | | | | |  |
|  | | | | | | | | | | | | 8 |  | S |  | 6/17/2026 9:14 PM |  |
|  | Yes, I am, more of, digital monkey. More on the softwares and, apps side. So yeah, I use a lot of them | | | | | | | | | | | | | | | |  |
|  |  | | | | | | | | | | | | | | | |  |
| Formatted Reports\\Coding Summary by Code Formatted Report | | | | | | | | | | Page 28 of 36 | | | | | | | |
| 6/18/2026 4:13 PM | | | | | | | | | | | | | | | | | |
|  | | | **Aggregate** |  | **Classification** |  | **Coverage** |  | **Number Of Coding References** | |  | **Reference Number** |  | **Coded By Initials** |  | **Modified On** |  |
|  | | | **Files\\Participant 5** | | | | | | | | | | | | | |  |
|  |  |  | No |  |  |  | 0.0678 |  | 16 | |  | | | | | |  |
|  | | |  |  |  |  |  |  |  | |  | | | | | | |
|  | | | | | | | | | | | | 1 |  | S |  | 6/17/2026 9:14 PM |  |
|  | some days I work from like 10 a.m. to 10 p.m. but other days I just do, like two drawings | | | | | | | | | | | | | | | |  |
|  |  | | | | | | | | | | | | | | | |  |
|  | | | | | | | | | | | | 2 |  | S |  | 6/17/2026 9:14 PM |  |
|  | I basically do is I finish most of the orders in the start of the month and the rest of the month I kind of keep for myself. | | | | | | | | | | | | | | | |  |
|  |  | | | | | | | | | | | | | | | |  |
|  | | | | | | | | | | | | 3 |  | S |  | 6/17/2026 9:14 PM |  |
|  | more likely it's it is my communication skills, something I get a lot from my customers | | | | | | | | | | | | | | | |  |
|  |  | | | | | | | | | | | | | | | |  |
|  | | | | | | | | | | | | 4 |  | S |  | 6/17/2026 9:14 PM |  |
|  | I know how to work my day off because I've been working with Krita for like like 3 or 4 years now. Right. So I think I'm pretty proficient in that | | | | | | | | | | | | | | | |  |
|  |  | | | | | | | | | | | | | | | |  |
|  | | | | | | | | | | | | 5 |  | S |  | 6/17/2026 9:14 PM |  |
|  | Usually message. I'm very like I, I talk to them like I talk to a friend, usually because I feel like that's the best way to, like, you know, make people feel comfortable. | | | | | | | | | | | | | | | |  |
|  |  | | | | | | | | | | | | | | | |  |
|  | | | | | | | | | | | | 6 |  | S |  | 6/17/2026 9:14 PM |  |
|  | it's always easier when you have like, the 1 to 1 connection | | | | | | | | | | | | | | | |  |
|  |  | | | | | | | | | | | | | | | |  |
|  | | | | | | | | | | | | 7 |  | S |  | 6/17/2026 9:14 PM |  |
|  | put up this wall between you and other person. And it feels that you can't be as comfortable. There's a bit more professionalism there. | | | | | | | | | | | | | | | |  |
|  |  | | | | | | | | | | | | | | | |  |
|  | | | | | | | | | | | | 8 |  | S |  | 6/17/2026 9:14 PM |  |
|  | the more people commission me, the more they'll tell their friends about me, and then they'll tell their friends | | | | | | | | | | | | | | | |  |
|  |  | | | | | | | | | | | | | | | |  |
|  | | | | | | | | | | | | 9 |  | S |  | 6/17/2026 9:14 PM |  |
|  | you know, like basically the liberty to allocate time to whatever I want as I see fit | | | | | | | | | | | | | | | |  |
|  |  | | | | | | | | | | | | | | | |  |
|  | | | | | | | | | | | | 10 |  | S |  | 6/17/2026 9:14 PM |  |
|  | when it comes to being at home and being a freelancer, you can just like the moment you're done with work, you can move on to something else, you know something else and be more productive. | | | | | | | | | | | | | | | |  |
|  |  | | | | | | | | | | | | | | | |  |
|  | | | | | | | | | | | | 11 |  | S |  | 6/17/2026 9:14 PM |  |
|  | the shortcuts and stuff I use up my muscle memory. So like, if it makes my work a lot faster | | | | | | | | | | | | | | | |  |
|  |  | | | | | | | | | | | | | | | |  |
|  | | | | | | | | | | | | 12 |  | S |  | 6/17/2026 9:14 PM |  |
|  | I like work way too much | | | | | | | | | | | | | | | |  |
|  |  | | | | | | | | | | | | | | | |  |
|  | | | | | | | | | | | | 13 |  | S |  | 6/17/2026 9:14 PM |  |
|  | I'm very happy right now with the customer base. | | | | | | | | | | | | | | | |  |
|  |  | | | | | | | | | | | | | | | |  |
|  | | | | | | | | | | | | 14 |  | S |  | 6/18/2026 3:18 PM |  |
|  | I actually find it encouraging when they give me feedback that positive or negative because that means that they really like care about the money that they invested in me. And they want you to turn Good. So if they give me an edit, that means that they like like That means that they genuinely love the thing I give them, you know So it's very motivating actually, the fact that they're like invested in that way. | | | | | | | | | | | | | | | |  |
|  |  | | | | | | | | | | | | | | | |  |
| Formatted Reports\\Coding Summary by Code Formatted Report | | | | | | | | | | Page 29 of 36 | | | | | | | |
| 6/18/2026 4:13 PM | | | | | | | | | | | | | | | | | |
|  | | | **Aggregate** |  | **Classification** |  | **Coverage** |  | **Number Of Coding References** | |  | **Reference Number** |  | **Coded By Initials** |  | **Modified On** |  |
|  | | | | | | | | | | | | | | | | | |
|  | | | | | | | | | | | | 15 |  | S |  | 6/17/2026 9:14 PM |  |
|  | having control over my own time is very important to me | | | | | | | | | | | | | | | |  |
|  |  | | | | | | | | | | | | | | | |  |
|  | | | | | | | | | | | | 16 |  | S |  | 6/17/2026 9:14 PM |  |
|  | I use Pinterest, because, that's something I'm using, like a platform that, sometimes I make a Pinterest board and send me because that gives me more than enough information of what I need, and so that it it's better to set. I'm pretty good at coming up with ideas in that sense | | | | | | | | | | | | | | | |  |
|  |  | | | | | | | | | | | | | | | |  |
|  | | | **Files\\Participant 6** | | | | | | | | | | | | | |  |
|  |  |  | No |  |  |  | 0.0625 |  | 21 | |  | | | | | |  |
|  | | |  |  |  |  |  |  |  | |  | | | | | | |
|  | | | | | | | | | | | | 1 |  | S |  | 6/17/2026 9:14 PM |  |
|  | the work I do now is part time because, I can focus on one thing specifically. I just have to manage with a whole bunch of other things, which, it gives me my own flexibility | | | | | | | | | | | | | | | |  |
|  |  | | | | | | | | | | | | | | | |  |
|  | | | | | | | | | | | | 2 |  | S |  | 6/17/2026 9:14 PM |  |
|  | there's like a lot of things that you have to learn from it, but like it's all about practice at the end of the day | | | | | | | | | | | | | | | |  |
|  |  | | | | | | | | | | | | | | | |  |
|  | | | | | | | | | | | | 3 |  | S |  | 6/18/2026 3:15 PM |  |
|  | I think I'm very confident in my video editing skills and of that and the, praise I have been given to. Yeah, the praise people have given me as also like, oh, like what clients I've talked about. It has given me more confidence in my abilities as a video editor, especially using Adobe. | | | | | | | | | | | | | | | |  |
|  |  | | | | | | | | | | | | | | | |  |
|  | | | | | | | | | | | | 4 |  | S |  | 6/17/2026 9:14 PM |  |
|  | lucky enough to have built a pretty big resume there myself | | | | | | | | | | | | | | | |  |
|  |  | | | | | | | | | | | | | | | |  |
|  | | | | | | | | | | | | 5 |  | S |  | 6/17/2026 9:14 PM |  |
|  | if they have like a good idea of what they want to do and incredible communication, then I'm all for it. | | | | | | | | | | | | | | | |  |
|  |  | | | | | | | | | | | | | | | |  |
|  | | | | | | | | | | | | 6 |  | S |  | 6/17/2026 9:14 PM |  |
|  | when it comes to some clients.So you know what they expect. | | | | | | | | | | | | | | | |  |
|  |  | | | | | | | | | | | | | | | |  |
|  | | | | | | | | | | | | 7 |  | S |  | 6/17/2026 9:14 PM |  |
|  | it's something I really enjoy doing in general. Like I really love video editing. So it wasn't exactly an issue for me | | | | | | | | | | | | | | | |  |
|  |  | | | | | | | | | | | | | | | |  |
|  | | | | | | | | | | | | 8 |  | S |  | 6/17/2026 9:14 PM |  |
|  | the ability to be flexible with your own time is so healthy | | | | | | | | | | | | | | | |  |
|  |  | | | | | | | | | | | | | | | |  |
|  | | | | | | | | | | | | 9 |  | S |  | 6/17/2026 9:14 PM |  |
|  | I want, like to pick up more gigs, but also that it's just the fact that you get to work with a lot of different, videos, like different video ideas and expertise. | | | | | | | | | | | | | | | |  |
|  |  | | | | | | | | | | | | | | | |  |
|  | | | | | | | | | | | | 10 |  | S |  | 6/17/2026 9:14 PM |  |
|  | I just had it scheduled in my head, and, like, select which gigs to prioritize and then just laid it down on a schedule | | | | | | | | | | | | | | | |  |
|  |  | | | | | | | | | | | | | | | |  |
|  | | | | | | | | | | | | 11 |  | S |  | 6/17/2026 9:14 PM |  |
|  | I'm very familiar now because I've been working with these softwares for quite some time | | | | | | | | | | | | | | | |  |
|  |  | | | | | | | | | | | | | | | |  |
|  | | | | | | | | | | | | 12 |  | S |  | 6/17/2026 9:14 PM |  |
|  | it's just more of the flexibility that I have. Because at a 9 to 5, it's a fixed timeline. | | | | | | | | | | | | | | | |  |
|  |  | | | | | | | | | | | | | | | |  |
| Formatted Reports\\Coding Summary by Code Formatted Report | | | | | | | | | | Page 30 of 36 | | | | | | | |
| 6/18/2026 4:13 PM | | | | | | | | | | | | | | | | | |
|  | | | **Aggregate** |  | **Classification** |  | **Coverage** |  | **Number Of Coding References** | |  | **Reference Number** |  | **Coded By Initials** |  | **Modified On** |  |
|  | | | | | | | | | | | | | | | | | |
|  | | | | | | | | | | | | 13 |  | S |  | 6/17/2026 9:14 PM |  |
|  | I'm very confident in myself as just a general video editor. So whatever video it is, I'll be very happy to work with it | | | | | | | | | | | | | | | |  |
|  |  | | | | | | | | | | | | | | | |  |
|  | | | | | | | | | | | | 14 |  | S |  | 6/17/2026 9:14 PM |  |
|  | It's pretty satisfying. Most of the work I do is really nice | | | | | | | | | | | | | | | |  |
|  |  | | | | | | | | | | | | | | | |  |
|  | | | | | | | | | | | | 15 |  | S |  | 6/17/2026 9:14 PM |  |
|  | the motivation, the that whole online persona have another book is like in just for me. It's like get more stuff onto my resume. | | | | | | | | | | | | | | | |  |
|  |  | | | | | | | | | | | | | | | |  |
|  | | | | | | | | | | | | 16 |  | S |  | 6/17/2026 9:14 PM |  |
|  | get some more exposure and you build up your resume, you're going to continue working at freelancer, but in the future you would your final goal would be to be. | | | | | | | | | | | | | | | |  |
|  |  | | | | | | | | | | | | | | | |  |
|  | | | | | | | | | | | | 17 |  | S |  | 6/17/2026 9:14 PM |  |
|  | point for positive feedback it so some does feel nice | | | | | | | | | | | | | | | |  |
|  |  | | | | | | | | | | | | | | | |  |
|  | | | | | | | | | | | | 18 |  | S |  | 6/17/2026 9:14 PM |  |
|  | end of the day. It's like a learning process, right? It's like you got to you try to listen to the criticism | | | | | | | | | | | | | | | |  |
|  |  | | | | | | | | | | | | | | | |  |
|  | | | | | | | | | | | | 19 |  | S |  | 6/17/2026 9:14 PM |  |
|  | getting used to and then like once you're used to it, then you keep going | | | | | | | | | | | | | | | |  |
|  |  | | | | | | | | | | | | | | | |  |
|  | | | | | | | | | | | | 20 |  | S |  | 6/17/2026 9:14 PM |  |
|  | then worked out it works out at the end of it gets simpler. | | | | | | | | | | | | | | | |  |
|  |  | | | | | | | | | | | | | | | |  |
|  | | | | | | | | | | | | 21 |  | S |  | 6/17/2026 9:14 PM |  |
|  | you learn more when you keep practicing and like, when you practice, like, you get much more fast | | | | | | | | | | | | | | | |  |
|  |  | | | | | | | | | | | | | | | |  |
|  | | | **Files\\Participant 7** | | | | | | | | | | | | | |  |
|  |  |  | No |  |  |  | 0.1673 |  | 24 | |  | | | | | |  |
|  | | |  |  |  |  |  |  |  | |  | | | | | | |
|  | | | | | | | | | | | | 1 |  | S |  | 6/17/2026 9:14 PM |  |
|  | the most important thing about our career is that, that we have experience in working in various projects. | | | | | | | | | | | | | | | |  |
|  |  | | | | | | | | | | | | | | | |  |
|  | | | | | | | | | | | | 2 |  | S |  | 6/17/2026 9:14 PM |  |
|  | I started my freelancing journey to get more, experience working with, different clients and working in different projects. | | | | | | | | | | | | | | | |  |
|  |  | | | | | | | | | | | | | | | |  |
|  | | | | | | | | | | | | 3 |  | S |  | 6/17/2026 9:14 PM |  |
|  | lots of benefit, in working as a freelancer in terms of finance and also in experience, working with different clients and, doing what you love. | | | | | | | | | | | | | | | |  |
|  |  | | | | | | | | | | | | | | | |  |
|  | | | | | | | | | | | | 4 |  | S |  | 6/17/2026 9:14 PM |  |
|  | when it comes to tasks, it's, mostly, the task I get assigned is to, frontend developing, right? So, frontend developing and, sometimes, QI/UX development. Yeah. So those two are the main two I focus on. | | | | | | | | | | | | | | | |  |
|  |  | | | | | | | | | | | | | | | |  |
|  | | | | | | | | | | | | 5 |  | S |  | 6/17/2026 9:14 PM |  |
|  | in the beginning, I was a little bit nervous because, when it comes to freelancing, you are all alone | | | | | | | | | | | | | | | |  |
|  |  | | | | | | | | | | | | | | | |  |
| Formatted Reports\\Coding Summary by Code Formatted Report | | | | | | | | | | Page 31 of 36 | | | | | | | |
| 6/18/2026 4:13 PM | | | | | | | | | | | | | | | | | |
|  | | | **Aggregate** |  | **Classification** |  | **Coverage** |  | **Number Of Coding References** | |  | **Reference Number** |  | **Coded By Initials** |  | **Modified On** |  |
|  | | | | | | | | | | | | | | | | | |
|  | | | | | | | | | | | | 6 |  | S |  | 6/17/2026 9:14 PM |  |
|  | So in the beginning, I, I was quite nervous about it, but with time, you know, I got used to it. And now I think I'm pretty confident about it. | | | | | | | | | | | | | | | |  |
|  |  | | | | | | | | | | | | | | | |  |
|  | | | | | | | | | | | | 7 |  | S |  | 6/17/2026 9:14 PM |  |
|  | So if the requirement is clear, it's easy for me to finish the work soon so that, you know we get less changes. So, yeah, good communication does make a difference. | | | | | | | | | | | | | | | |  |
|  |  | | | | | | | | | | | | | | | |  |
|  | | | | | | | | | | | | 8 |  | S |  | 6/17/2026 9:14 PM |  |
|  | because I was able to communicate easily with the client I had to get the requirements straight. I was able to finish the website just in three days. | | | | | | | | | | | | | | | |  |
|  |  | | | | | | | | | | | | | | | |  |
|  | | | | | | | | | | | | 9 |  | S |  | 6/17/2026 9:14 PM |  |
|  | I would say with flexibility and everything, I pick online freelancing. | | | | | | | | | | | | | | | |  |
|  |  | | | | | | | | | | | | | | | |  |
|  | | | | | | | | | | | | 10 |  | S |  | 6/17/2026 9:14 PM |  |
|  | I am someone who values my time and I think, you know, being stuck in a 9 to 5 job it will limit your ability. | | | | | | | | | | | | | | | |  |
|  |  | | | | | | | | | | | | | | | |  |
|  | | | | | | | | | | | | 11 |  | S |  | 6/17/2026 9:14 PM |  |
|  | you know, have to, you know, stay in a certain environment and implement what is told to you. It doesn't give you a free mindset to work on your own. | | | | | | | | | | | | | | | |  |
|  |  | | | | | | | | | | | | | | | |  |
|  | | | | | | | | | | | | 12 |  | S |  | 6/17/2026 9:14 PM |  |
|  | So when it comes to freelancing it to help you learn and also to explore new, things and new idea. So, that way I prefer, that's why I prefer online freelancing. | | | | | | | | | | | | | | | |  |
|  |  | | | | | | | | | | | | | | | |  |
|  | | | | | | | | | | | | 13 |  | S |  | 6/18/2026 3:57 PM |  |
|  | Every time I take a new gig, it's like a new, project, a new client. So it's like a new learning experience. | | | | | | | | | | | | | | | |  |
|  |  | | | | | | | | | | | | | | | |  |
|  | | | | | | | | | | | | 14 |  | S |  | 6/17/2026 9:14 PM |  |
|  | since I am from a software background, I think when I see an issue that doesn't demotivate me or anything. So because I believe, you know, that's fixable. So, yeah, I don't think it makes a difference in my work. | | | | | | | | | | | | | | | |  |
|  |  | | | | | | | | | | | | | | | |  |
|  | | | | | | | | | | | | 15 |  | S |  | 6/17/2026 9:14 PM |  |
|  | from my start to now, I mean, I have improved a lot | | | | | | | | | | | | | | | |  |
|  |  | | | | | | | | | | | | | | | |  |
|  | | | | | | | | | | | | 16 |  | S |  | 6/17/2026 9:14 PM |  |
|  | rather than working in a 9 to 5 job and doing the same thing over and over again with again, me working with different, clients and different projects that improve me a lot. And has improved my knowledge a lot. I think it has made and it has given me to manage my time more efficiently and effectively | | | | | | | | | | | | | | | |  |
|  |  | | | | | | | | | | | | | | | |  |
|  | | | | | | | | | | | | 17 |  | S |  | 6/17/2026 9:14 PM |  |
|  | since I have been working for a while, and since I have a knowledge in this, I think it makes me finish my task. | | | | | | | | | | | | | | | |  |
|  |  | | | | | | | | | | | | | | | |  |
|  | | | | | | | | | | | | 18 |  | S |  | 6/18/2026 4:00 PM |  |
|  | Okay, this is the. I'm going to get this task today at this time. And I'll make it completed at the end of this time. So like without having a constant or a specific time period do my task. You know, since I have the flexibility to do the task whenever I want within the day, like according to my, schedule and my timeline because, when it comes to a 9 to 5 job, usually the timeline for your work is set by another person, right? | | | | | | | | | | | | | | | |  |
|  |  | | | | | | | | | | | | | | | |  |
|  | | | | | | | | | | | | 19 |  | S |  | 6/17/2026 9:14 PM |  |
|  | But when it comes to freelancing, that's not the case because you are timeline by you itself. So I think that flexibility is what drives me more towards freelancing. | | | | | | | | | | | | | | | |  |
|  |  | | | | | | | | | | | | | | | |  |
| Formatted Reports\\Coding Summary by Code Formatted Report | | | | | | | | | | Page 32 of 36 | | | | | | | |
| 6/18/2026 4:13 PM | | | | | | | | | | | | | | | | | |
|  | | | **Aggregate** |  | **Classification** |  | **Coverage** |  | **Number Of Coding References** | |  | **Reference Number** |  | **Coded By Initials** |  | **Modified On** |  |
|  | | | | | | | | | | | | | | | | | |
|  | | | | | | | | | | | | 20 |  | S |  | 6/17/2026 9:14 PM |  |
|  | With my with my past experience, if I have a similar project, I know, you know, it's easy for me to get this done with this time because I have a specific idea, because I have done that project, a similar project or similar, what do you call, similar tool, like, if I have done that project already with my, in my past work, obviously, I know how long it would take me to do this thing.So I have a better understanding of the timeline. And, yeah. So I think definitely it has an effect and it helps me get the work done faster than the normal project. | | | | | | | | | | | | | | | |  |
|  |  | | | | | | | | | | | | | | | |  |
|  | | | | | | | | | | | | 21 |  | S |  | 6/17/2026 9:14 PM |  |
|  | I think, yeah, sometimes, you know, you get this, hard client, you just feel like just giving up on everything, but, but. Yeah, but some clients are very understanding. Like, I think I gave an example on how time sometimes can be not understanding when it comes to the timeline, | | | | | | | | | | | | | | | |  |
|  |  | | | | | | | | | | | | | | | |  |
|  | | | | | | | | | | | | 22 |  | S |  | 6/17/2026 9:14 PM |  |
|  | Sometimes, you know, even if it's, if it's an advice, if if it's if it comes in a nicer way, I think it will motivate me to do better. | | | | | | | | | | | | | | | |  |
|  |  | | | | | | | | | | | | | | | |  |
|  | | | | | | | | | | | | 23 |  | S |  | 6/17/2026 9:14 PM |  |
|  | So adaptability is challenging, but I think, as I told you, since I am from a software background, I think I can take it up, but it's definitely challenging. | | | | | | | | | | | | | | | |  |
|  |  | | | | | | | | | | | | | | | |  |
|  | | | | | | | | | | | | 24 |  | S |  | 6/17/2026 9:14 PM |  |
|  | when I started off, my confidence was really low. But since I have this experience and I know how this works and, all these things. So I think now with my I have a pretty good confidence in working online. | | | | | | | | | | | | | | | |  |
|  |  | | | | | | | | | | | | | | | |  |
|  | | | **Files\\Participant 8** | | | | | | | | | | | | | |  |
|  |  |  | No |  |  |  | 0.1447 |  | 22 | |  | | | | | |  |
|  | | |  |  |  |  |  |  |  | |  | | | | | | |
|  | | | | | | | | | | | | 1 |  | S |  | 6/17/2026 9:14 PM |  |
|  | whenever I have work, I try to fit it into my schedule with all the other things that I do | | | | | | | | | | | | | | | |  |
|  |  | | | | | | | | | | | | | | | |  |
|  | | | | | | | | | | | | 2 |  | S |  | 6/17/2026 9:14 PM |  |
|  | When it comes to graphic designing, I don't, I stick to more basic kind of graphic designing. So I mostly use Canva for that. | | | | | | | | | | | | | | | |  |
|  |  | | | | | | | | | | | | | | | |  |
|  | | | | | | | | | | | | 3 |  | S |  | 6/17/2026 9:14 PM |  |
|  | I, shoot content with my phone and then edit those stuff into, like, 30 to 40 second reels or maybe lesser, on Capcut. | | | | | | | | | | | | | | | |  |
|  |  | | | | | | | | | | | | | | | |  |
|  | | | | | | | | | | | | 4 |  | S |  | 6/17/2026 9:14 PM |  |
|  | whenever they send us like in inspo and, like a brief of what exactly they want, it's usually more easier to finish it. | | | | | | | | | | | | | | | |  |
|  |  | | | | | | | | | | | | | | | |  |
|  | | | | | | | | | | | | 5 |  | S |  | 6/17/2026 9:14 PM |  |
|  | Progressively I feel like I have gotten more confident. | | | | | | | | | | | | | | | |  |
|  |  | | | | | | | | | | | | | | | |  |
|  | | | | | | | | | | | | 6 |  | S |  | 6/17/2026 9:14 PM |  |
|  | basic stuff, which I was not aware of when I was, like, learning them myself. And then progressively, I also got into more self-learning and like watching YouTube videos and experimenting and all that | | | | | | | | | | | | | | | |  |
|  |  | | | | | | | | | | | | | | | |  |
|  | | | | | | | | | | | | 7 |  | S |  | 6/17/2026 9:14 PM |  |
|  | I do prefer freelancing than a 9 to 5 personally, because I do a lot of other things. So when I'm stuck in a 9 to 5, I have to beg the management for like leaves and I don’t have the freedom to, work whenever I want. Right. Which is why I, started freelancing and and which is why I'm trying to stick to it. | | | | | | | | | | | | | | | |  |
|  |  | | | | | | | | | | | | | | | |  |
|  | | | | | | | | | | | | 8 |  | S |  | 6/17/2026 9:14 PM |  |
|  | I try to find more gigs that are different than the ones that I usually do sometimes, because then I get more experience in terms of, like, different kinds of work. And I try to I try to find more, more clients like, who are different to each other, like in different, how do I say it, in different aspects of work, so that I understand all those aspects of work. | | | | | | | | | | | | | | | |  |
|  |  | | | | | | | | | | | | | | | |  |
| Formatted Reports\\Coding Summary by Code Formatted Report | | | | | | | | | | Page 33 of 36 | | | | | | | |
| 6/18/2026 4:13 PM | | | | | | | | | | | | | | | | | |
|  | | | **Aggregate** |  | **Classification** |  | **Coverage** |  | **Number Of Coding References** | |  | **Reference Number** |  | **Coded By Initials** |  | **Modified On** |  |
|  | | | | | | | | | | | | | | | | | |
|  | | | | | | | | | | | | 9 |  | S |  | 6/17/2026 9:14 PM |  |
|  | Since I’m not in a 9 to 5, there's no one to like pinpoint and teach me. Okay. Do try, try to learn this try to learn that. So I try to find different types of clients so that I can learn through the process. | | | | | | | | | | | | | | | |  |
|  |  | | | | | | | | | | | | | | | |  |
|  | | | | | | | | | | | | 10 |  | S |  | 6/17/2026 9:14 PM |  |
|  | Sometimes it's hard to like pick which gig to choose and all that. So then I try to, work through it, even if I feel like I can't do it. I try to like, find ways to learn how to do that, then, like, get it done. | | | | | | | | | | | | | | | |  |
|  |  | | | | | | | | | | | | | | | |  |
|  | | | | | | | | | | | | 11 |  | S |  | 6/17/2026 9:14 PM |  |
|  | Compared to when I started and where I'm, where I am now, I feel like my video editing has gotten comparatively better. because I feel like I do experiment with, like, transitions and all of that in Capcut. So yes, that does affect how much work I put into, particularly gigs, and how much I am willing to, like, go into it. | | | | | | | | | | | | | | | |  |
|  |  | | | | | | | | | | | | | | | |  |
|  | | | | | | | | | | | | 12 |  | S |  | 6/17/2026 9:14 PM |  |
|  | with experience, I have gotten better in like using the tools and all of that. so I feel like the quality of work has gotten better whenever I am more keen on learning about the platform and all that. | | | | | | | | | | | | | | | |  |
|  |  | | | | | | | | | | | | | | | |  |
|  | | | | | | | | | | | | 13 |  | S |  | 6/17/2026 9:14 PM |  |
|  | I am in control of the time that I work. | | | | | | | | | | | | | | | |  |
|  |  | | | | | | | | | | | | | | | |  |
|  | | | | | | | | | | | | 14 |  | S |  | 6/17/2026 9:14 PM |  |
|  | I still have the option to get the work done even at night if I want. | | | | | | | | | | | | | | | |  |
|  |  | | | | | | | | | | | | | | | |  |
|  | | | | | | | | | | | | 15 |  | S |  | 6/17/2026 9:14 PM |  |
|  | whenever I have to attend other stuff, if I'm not available during the usual work hours, it's very important to me, that I have the choice to come home, at, like, maybe, like, ten in the night and still get things done.So that is very important to me because it gives me the opportunity to do other things that I’m passionate about. | | | | | | | | | | | | | | | |  |
|  |  | | | | | | | | | | | | | | | |  |
|  | | | | | | | | | | | | 16 |  | S |  | 6/17/2026 9:14 PM |  |
|  | Since no one is like, forcing me to do like, certain tasks. Yes. I feel more confident and more willing to take more gigs so I can, like, choose what I can do. | | | | | | | | | | | | | | | |  |
|  |  | | | | | | | | | | | | | | | |  |
|  | | | | | | | | | | | | 17 |  | S |  | 6/17/2026 9:14 PM |  |
|  | I feel like I am pretty satisfied with the work | | | | | | | | | | | | | | | |  |
|  |  | | | | | | | | | | | | | | | |  |
|  | | | | | | | | | | | | 18 |  | S |  | 6/17/2026 9:14 PM |  |
|  | There are instances where I believe the output could have been better, but at the end of the day, if the clients like it, I am okay with it. | | | | | | | | | | | | | | | |  |
|  |  | | | | | | | | | | | | | | | |  |
|  | | | | | | | | | | | | 19 |  | S |  | 6/17/2026 9:14 PM |  |
|  | Like the client wants what they want so I have to somehow keep my motivation up and get it done. So I feel like with experience it has gotten better, | | | | | | | | | | | | | | | |  |
|  |  | | | | | | | | | | | | | | | |  |
|  | | | | | | | | | | | | 20 |  | S |  | 6/17/2026 9:14 PM |  |
|  | whenever I am typing stuff I use Grammarly to like check the spellings and grammar rather than putting that also into Chatgpt which also saves time. | | | | | | | | | | | | | | | |  |
|  |  | | | | | | | | | | | | | | | |  |
|  | | | | | | | | | | | | 21 |  | S |  | 6/17/2026 9:14 PM |  |
|  | Notion kind of helps me to be on track of everything. Cause everything is in place and I feel like its more user friendly, we do. | | | | | | | | | | | | | | | |  |
|  |  | | | | | | | | | | | | | | | |  |
|  | | | | | | | | | | | | 22 |  | S |  | 6/17/2026 9:14 PM |  |
|  | since I have been doing for a while, I am pretty confident. | | | | | | | | | | | | | | | |  |
|  |  | | | | | | | | | | | | | | | |  |
| Formatted Reports\\Coding Summary by Code Formatted Report | | | | | | | | | | Page 34 of 36 | | | | | | | |
| 6/18/2026 4:13 PM | | | | | | | | | | | | | | | | | |
|  | | | **Aggregate** |  | **Classification** |  | **Coverage** |  | **Number Of Coding References** | |  | **Reference Number** |  | **Coded By Initials** |  | **Modified On** |  |
|  | | | **Files\\Participant 9** | | | | | | | | | | | | | |  |
|  |  |  | No |  |  |  | 0.1469 |  | 15 | |  | | | | | |  |
|  | | |  |  |  |  |  |  |  | |  | | | | | | |
|  | | | | | | | | | | | | 1 |  | S |  | 6/17/2026 9:14 PM |  |
|  | I have been using, both of those, softwares, close to close to 7 to 8, 7 to 8 years. So, yeah, I am confident in using this softwares. | | | | | | | | | | | | | | | |  |
|  |  | | | | | | | | | | | | | | | |  |
|  | | | | | | | | | | | | 2 |  | S |  | 6/17/2026 9:14 PM |  |
|  | So the client clearly gave me the instructions that they want, you know, in the within the picture. So I was able to complete the tasks since I was given a very clear, clear view of what the client needs. So I was able to quickly finish the job. And, and satisfy the customer. | | | | | | | | | | | | | | | |  |
|  |  | | | | | | | | | | | | | | | |  |
|  | | | | | | | | | | | | 3 |  | S |  | 6/17/2026 9:14 PM |  |
|  | They usually give reference features, pictures or reference ideas, which they want me to do. So then, it it will clear any doubts which I have during the task. | | | | | | | | | | | | | | | |  |
|  |  | | | | | | | | | | | | | | | |  |
|  | | | | | | | | | | | | 4 |  | S |  | 6/17/2026 9:14 PM |  |
|  | I'm not really 100% familiar with what I'm doing. So at that time, I have to, search on the internet, on YouTube, platform, other forums, to find my solution. | | | | | | | | | | | | | | | |  |
|  |  | | | | | | | | | | | | | | | |  |
|  | | | | | | | | | | | | 5 |  | S |  | 6/17/2026 9:14 PM |  |
|  | I have improved a lot with time. So I have been able to I have been able to complete task more efficiently and effectively than usual. Then what I did, previously. | | | | | | | | | | | | | | | |  |
|  |  | | | | | | | | | | | | | | | |  |
|  | | | | | | | | | | | | 6 |  | S |  | 6/17/2026 9:14 PM |  |
|  | Since I have a week to do a gig, I take only one gig per week. I have more time. I can I can adjust my, 9 to 5 job | | | | | | | | | | | | | | | |  |
|  |  | | | | | | | | | | | | | | | |  |
|  | | | | | | | | | | | | 7 |  | S |  | 6/17/2026 9:14 PM |  |
|  | the more gigs I do, the more better I become in using the application. So with time, yes, I, I have improved my skills and I will improve, in the future. | | | | | | | | | | | | | | | |  |
|  |  | | | | | | | | | | | | | | | |  |
|  | | | | | | | | | | | | 8 |  | S |  | 6/17/2026 9:14 PM |  |
|  | I am in control of how many gigs I take per week or how many gigs I take, overall. | | | | | | | | | | | | | | | |  |
|  |  | | | | | | | | | | | | | | | |  |
|  | | | | | | | | | | | | 9 |  | S |  | 6/17/2026 9:14 PM |  |
|  | I have the freedom of, when to do my when and where to do my work. Whereas if I work, as a 9 to 5 job, as a 9 to 5, employee, I have a fixed, fixed time time schedule where I have to be. I have to start my work when I have, and when I have to finish my work and yeah, those kind of stuff. So I prefer this. | | | | | | | | | | | | | | | |  |
|  |  | | | | | | | | | | | | | | | |  |
|  | | | | | | | | | | | | 10 |  | S |  | 6/17/2026 9:14 PM |  |
|  | the freedom it gives me. I prefer it a lot. | | | | | | | | | | | | | | | |  |
|  |  | | | | | | | | | | | | | | | |  |
|  | | | | | | | | | | | | 11 |  | S |  | 6/17/2026 9:14 PM |  |
|  | since I do photography, and editorial stuff like, photo retouching and, color grading images is what I do anyway, so, so I am so, so I am, very familiar with what I do | | | | | | | | | | | | | | | |  |
|  |  | | | | | | | | | | | | | | | |  |
|  | | | | | | | | | | | | 12 |  | S |  | 6/17/2026 9:14 PM |  |
|  | It depends on the review or the feedback, which I get, I try my best to take, the feedback as a constructive criticism, without taking it personally. So if I, if there's anything which I can be, which I can improve in my skill, I would, I would do it. If not, if it's if not, I would, just disregard the review, which I have gotten | | | | | | | | | | | | | | | |  |
|  |  | | | | | | | | | | | | | | | |  |
|  | | | | | | | | | | | | 13 |  | S |  | 6/17/2026 9:14 PM |  |
|  | Positive or negative. As long as it's constructive. I am happy. | | | | | | | | | | | | | | | |  |
|  |  | | | | | | | | | | | | | | | |  |
|  | | | | | | | | | | | | 14 |  | S |  | 6/17/2026 9:14 PM |  |
|  | I find it very easy and convenient, whereas, without physically meeting people and, doing, like, doing all the hassle. It's, it's pretty easy to do the gigs online and to get paid instantly. | | | | | | | | | | | | | | | |  |
|  |  | | | | | | | | | | | | | | | |  |
| Formatted Reports\\Coding Summary by Code Formatted Report | | | | | | | | | | Page 35 of 36 | | | | | | | |
| 6/18/2026 4:13 PM | | | | | | | | | | | | | | | | | |
|  | | | **Aggregate** |  | **Classification** |  | **Coverage** |  | **Number Of Coding References** | |  | **Reference Number** |  | **Coded By Initials** |  | **Modified On** |  |
|  | | | | | | | | | | | | | | | | | |
|  | | | | | | | | | | | | 15 |  | S |  | 6/17/2026 9:14 PM |  |
|  | with time I have been, I have gotten more skilled, like I have I have no the I have gotten to know the inside and out of the computer | | | | | | | | | | | | | | | |  |
|  |  | | | | | | | | | | | | | | | |  |
|  | | | | | | | | | | | | | | | | | |
|  | | | | | | | | | | | | | | | | | |
|  | | | | | | | | | | | | | | | | | |
|  | | | | | | | | | | | | | | | | | |
|  | | | | | | | | | | | | | | | | | |
|  | | | | | | | | | | | | | | | | | |
|  | | | | | | | | | | | | | | | | | |
|  | | | | | | | | | | | | | | | | | |
|  | | | | | | | | | | | | | | | | | |
| Formatted Reports\\Coding Summary by Code Formatted Report | | | | | | | | | | Page 36 of 36 | | | | | | | |
