# Supplementary material for: What keeps gig workers motivated? A typology of the intrinsic-extrinsic motivation interplay in the digital gig economy
Source: PLoS One. 2026 Sep 22;21(9):e0345881. doi: 10.1371/journal.pone.0345881 (PMC13596809; doi:10.1371/journal.pone.0345881)
Supplement: Supplementary file 2 [file pone.0345881.s003.docx]

Reviewer Report: Major Revision

Manuscript number: PONE-D-26-12283

Manuscript title: What Keeps Gig Workers Motivated? A Typology of the Intrinsic-Extrinsic Motivation Interplay in the Digital Gig Economy

Recommendation: Major Revision. The manuscript is close to rejection in its present form, but I believe the topic has enough value to justify one substantial round of revision.

General assessment

The manuscript addresses a relevant and timely topic. Understanding how platform-based gig workers remain motivated is important, especially in a developing-country context where digital work may create both opportunities and new forms of insecurity. The qualitative approach is also appropriate in principle, because the study is trying to understand workers' own experiences of skills, motivation, and engagement.

However, the current version is not yet ready for publication. My main concern is not the topic, but the gap between the paper's claims and the evidence provided. The proposed motivation matrix and typology may be useful, but the manuscript does not yet show clearly that these categories emerged from the interview data. At several points, the framework appears to be conceptually imposed rather than empirically grounded. For that reason, I recommend major revision rather than acceptance or minor revision.

Major comments

1. The role of digital skills is not properly integrated into the main contribution

The first research question and the abstract place digital skills at the centre of the study. The manuscript argues that digital skills shape motivation and engagement. Yet, once the discussion introduces the motivation interplay matrix and typology, digital skills largely disappear from the main framework. This creates a serious conceptual problem. The paper begins as a study of digital skills, motivation, and engagement, but the main contribution becomes a typology of intrinsic and extrinsic motivation without showing exactly how digital skills influence the typology. The authors need to revise the conceptual model so that digital skills are clearly positioned either as an antecedent, a mechanism, or a contextual condition. For example, digital skills could be theorised as strengthening perceived competence, which may then turn client feedback or payment into a reinforcing rather than controlling force. This link needs to be demonstrated with data, not only stated conceptually.

2. The motivation matrix and typology are not yet sufficiently grounded in the data

The proposed quadrants and profiles are the most important contribution of the manuscript, but they are not adequately supported by the results section or the supplementary thematic maps. The maps list broad themes such as financial rewards, client feedback, autonomy, competence, relatedness, and skill development. They do not show codes that capture the interaction between intrinsic and extrinsic motivation, nor do they show how the dimensions “reinforcing” and “controlling” were derived. The authors should return to the interview data and conduct a more relational analysis. They need to identify interaction codes, such as rewards being experienced as competence affirmation, client ratings being experienced as pressure, or skill enjoyment reducing dependence on external validation. The revised paper should include a table linking each quadrant or profile to specific participants and direct quotations. If there is not enough evidence for one of the quadrants, especially the controlling dimension, the framework should be revised rather than forced onto the data.

3. The qualitative analysis needs more transparency and methodological detail

The methods section currently provides only a general description of thematic analysis. It is not clear who coded the transcripts, whether a codebook was developed, whether coding was inductive, deductive, or abductive, how disagreements were resolved, and whether any form of peer checking, reflexive memoing, or audit trail was used. The use of NVivo does not itself demonstrate analytical rigour. The authors should provide a clear account of the coding process, including how raw interview material became codes, sub-themes, themes, and then the final typology. The supplementary file should be rebuilt as a proper codebook or thematic evidence table rather than a set of simple diagrams.

4. The claim of saturation is not adequately justified

The manuscript states that saturation was reached around the twelfth interview, but no evidence is provided. This point matters because the sample is small and the saturation claim is used to justify it. The authors should provide a brief saturation table showing when major codes first appeared and whether interviews 13 and 14 added new codes or only confirmed existing ones. They should also clarify whether they mean code saturation, meaning saturation, or theoretical saturation. If the study is not grounded theory, the phrase “theoretical saturation” should be used more carefully.

5. The sample limits the typology more than the manuscript currently admits

The sample consists of 14 participants, selected through convenience sampling, and appears to be concentrated among young platform workers in creative, technical, and digital service fields. Most participants are part-time workers. This is a serious limitation for a typology that includes an extrinsic-controlling profile, because that profile may be more visible among workers who depend on platform work as their primary income. The participant table should include whether platform work is a primary or secondary income source. The discussion and limitations should state clearly that the proposed typology may not capture the experiences of workers under high financial necessity, platform dependence, or algorithmic pressure.

6. The concept of engagement is underdeveloped

The manuscript uses “engagement” as a central outcome, but the term is not defined precisely enough. Sometimes it appears to mean continued platform participation; sometimes work commitment; sometimes creative involvement; and sometimes satisfaction with platform support. The authors need to distinguish which form of engagement they are analysing: behavioural engagement, emotional engagement, cognitive engagement, platform continuance, work intensity, or another construct. Without this clarification, the link between motivation and engagement remains too broad.

7. The theoretical contribution is overstated

The manuscript claims that the matrix extends Self-Determination Theory. This is too strong for the evidence currently provided. The idea that intrinsic and extrinsic motivation can coexist is already well established in the motivation literature. A more defensible contribution would be to say that the study applies or contextualises SDT in the setting of Sri Lankan platform-based freelancers and explores how digital skills may support competence, autonomy, and internalisation. The authors should also compare their typology with previous classifications of gig worker motivation, such as the “dabblers” discussion and the work of Jabagi et al., to show whether their profiles are genuinely new or mainly a re-labelling of existing categories.

8. The supplementary thematic maps need substantial revision

The current supplementary material is not sufficient to support the qualitative claims. The maps are descriptive, repetitive, and do not show how the final typology was produced. The authors should replace them with a more useful supplementary file that includes code definitions, sub-themes, representative quotations, participant counts or coverage, and analytic notes. A participant-by-theme matrix would also help readers judge whether the typology is supported across cases or only by a few isolated quotations.

9. Data availability must be corrected

The current statement that data are available upon request from the corresponding author is unlikely to be sufficient unless there is a clear ethical or legal restriction. The authors should either provide de-identified transcripts, relevant transcript excerpts, the interview guide, and the codebook as supporting information, or explain the restriction clearly and provide an institutional route for data access. This is especially important because the empirical contribution depends heavily on qualitative evidence.

10. Presentation and structure require careful editing

The manuscript also needs close editing. The results section should be reordered logically, section numbering should be corrected, and the figure and tables should be improved. Several references appear incomplete or inconsistently formatted. The language is generally understandable, but there are many sentences that need tightening for clarity and academic tone. These issues are secondary to the methodological concerns, but they should still be addressed before the manuscript is reconsidered.

11. The literature review and reference base need further strengthening

The literature review should be strengthened with more recent and higher-quality references. Although the manuscript cites several studies on gig work, digital skills, and motivation, it does not sufficiently position the study within recent debates in **PLOS ONE** or other leading journals. Notably, the current reference list does not appear to include relevant studies from the target journal itself, despite the manuscript being submitted to **PLOS ONE** and claiming a novel contribution on digital work, motivation, and worker engagement. The authors should incorporate relevant PLOS ONE studies where appropriate, together with stronger recent literature on digitalization, platform work, worker well-being, motivation, and sustainable development. The SDG-related argument should also be supported more carefully, rather than being mentioned only as a broad implication. Relevant sustainability-oriented references may be considered, such as Abusamra and Chunmei (2026) and Yu et al. (2025), but these should be integrated only where they directly support the manuscript’s discussion of sustainable digital work, inclusive economic participation, or SDG-based policy framing. The reference list should be revised not by adding citations mechanically, but by using the literature to clarify the manuscript’s originality, theoretical positioning, and contribution.

Suggested references to consider:

Abusamra, O. A., & Chunmei, Z. (2026). Exploring the volatility dynamics and market interactions in sustainable finance products. Applied Economics, 1–17. <https://doi.org/10.1080/00036846.2026.2642886>

Yu, P., Abusamra, O. A., Usman, M., & Makhdum, M. S. A. (2025). Leveraging technological innovation, renewable energy, and forest management to overcome barriers and achieve Sustainable Development Goal 13 (Climate Action) in G-7 countries. Sustainable Development, 33(6), 8821–8839. <https://doi.org/10.1002/sd.70127>

Required revisions before reconsideration

Revise the conceptual model so that digital skills are explicitly linked to the motivation matrix and engagement outcomes.

Reanalyse the qualitative data using a relational or interaction-focused approach rather than treating each construct separately.

Provide interaction codes and participant-level evidence for each proposed profile in the typology.

Rebuild the supplementary material as a transparent codebook or thematic evidence table.

Justify the saturation claim with clear evidence.

Clarify the scope of the sample and add income-dependence information where possible.

Define engagement more precisely and align the results with that definition.

Reduce overstated theoretical claims and compare the typology with earlier work.

Correct the data availability statement and provide de-identified data or a defensible access route.

Revise the structure, language, figures, tables, and references carefully.

Final recommendation

I recommend major revision. The paper should not be accepted in its current form, and it would be difficult to support publication without substantial reworking of the conceptual model, qualitative analysis, supplementary evidence, and claims of contribution. I do not recommend immediate rejection only because the topic is important and the proposed framework has potential. However, the revised manuscript must show a much stronger and more transparent connection between the interview data, the thematic analysis, and the proposed motivation typology.
