# Supplementary material for: What keeps gig workers motivated? A typology of the intrinsic-extrinsic motivation interplay in the digital gig economy
Source: PLoS One. 2026 Sep 22;21(9):e0345881. doi: 10.1371/journal.pone.0345881 (PMC13596809; doi:10.1371/journal.pone.0345881)
Supplement: Supplementary file 3 [file pone.0345881.s005.docx]

**Point by point responses to the editor/reviewer comments.**

Dear Editor and Reviewers

Greetings

We extend our sincere appreciation for the valuable comments provided. We are genuinely grateful for the opportunity given to submit the revised version of our manuscript, titled “What Keeps Gig Workers Motivated? A Typology of the Intrinsic-Extrinsic Motivation Interplay in the Digital Gig Economy”.

We would like to express our gratitude for your dedicated time and effort and the insightful feedback from the editor and two reviewers, which has significantly enhanced the quality of our article. We have diligently incorporated the suggested improvements, and the revised document includes marked modifications. For your convenience, we have prepared a table detailing the changes, with line numbers corresponding to the revised manuscript incorporating track changes. Your continued support and guidance are highly valued, and we look forward to any further insights you or the reviewers may offer.

Please find the reference table below, aligning with the line numbers in the revised manuscript containing track changes. **(*** Please note that the manuscript with the track changes is attached towards the end of this document)**.

| **Editor’s Comments** | **Response to the Comment** |
| --- | --- |
| 1. Please ensure that your manuscript meets PLOS ONE's style requirements, including those for file naming. The PLOS ONE style templates can be found at <https://journals.plos.org/plosone/s/file?id=wjVg/PLOSOne_formatting_sample_main_body.pdf> and [https://journals.plos.org/plosone/s/file?id=ba62/PLOSOne_formatting_sample_title_authors_affiliations.pdf](https://track.editorialmanager.com/CL0/https:%2F%2Fjournals.plos.org%2Fplosone%2Fs%2Ffile%3Fid=ba62%2FPLOSOne_formatting_sample_title_authors_affiliations.pdf/1/010f019e247e2f19-30849d11-cb1f-4a21-b609-91866b683402-000000/-HpOTmFZUQjk-nvPUub2AmJ_rqad3w45tBQIGNEGjuo=258) | Thank you very much for bringing this to our attention.  In line with the feedback, we have included a revised first page according to the journal guidelines. |
| 2. In the ethics statement in the Methods, you have specified that verbal consent was obtained. Please provide additional details regarding how this consent was documented and witnessed, and state whether this was approved by the IRB. | Thank you very much for bringing this to our attention. Ethical approval was secured from the Sri Lanka Institute of Information Technology Ethics Review Committee prior to commencing the data collection. Next, verbal consent was established prior to commencing the interviews and was audio recorded alongside the interview itself. Hence, the interview transcripts also document the consent obtained. Additionally, the relevant transcripts have been handed over to the institute’s Ethics Review Committee Head, Professor Wasantha Rajapaksha.  This comment has been addressed in the revised manuscript as follows **(Line 334 -345)**:  *“This study rigorously adhered to established ethical standards surrounding primary research that incorporates human participants. An ethical approval was secured from Sri Lanka Institute of Information Technology Ethics Review Committee prior to data collection. The ethical approval reference no: SLIIT/ERC/SBS/2025/20 grants permission to conduct this study in all phases including participant recruitment and data analysis. In line with the institution’s ethics compliance process, all interview transcripts have been submitted to the Ethics Review committee head, Professor Wasantha Rajapaksha.* *Hence, all transcripts have been deposited in the institutional repository. Additionally, all participants interviewed were thoroughly informed regarding details surrounding the study’s purpose, contribution, data usage, and the voluntary right to withdraw participation during any stage of the interview. Consent was obtained from all participants through a verbal reaffirmation of their rights prior to conducting each interview session whereby each verbal consent was recorded.”* |
| 3. In the online submission form, you indicated that the data underlying the results presented in the study are available upon a written request to corresponding author.  All PLOS journals now require all data underlying the findings described in their manuscript to be freely available to other researchers, either 1. In a public repository, 2. Within the manuscript itself, or 3. Uploaded as supplementary information.  This policy applies to all data except where public deposition would breach compliance with the protocol approved by your research ethics board. If your data cannot be made publicly available for ethical or legal reasons (e.g., public availability would compromise patient privacy), please explain your reasons on resubmission and your exemption request will be escalated for approval. | Thank you very much for your valuable feedback. In line with PLOS journal policy, two supplementary files have been uploaded to make the data underlying the study freely accessible. S1 contains an Nvivo codebook documenting the first stage of the coding process. S2 contains a manual codebook documenting the major codes developed. Accordingly, the title page has been amended to reflect this change.  Ethical approval was secured from the Sri Lanka Institute of Information Technology Ethics Review Committee prior to commencing the data collection. Next, verbal consent was established prior to commencing the interviews and was audio recorded alongside the interview itself. Hence, the interview transcripts also document the consent obtained. Additionally, the relevant transcripts have been handed over to the institute’s Ethics Review Committee Head, Professor Wasantha Rajapaksha.  This comment has been addressed in the revised manuscript as follows **(Line 334 -345)**:  *“This study rigorously adhered to established ethical standards surrounding primary research that incorporates human participants. An ethical approval was secured from Sri Lanka Institute of Information Technology Research Ethics Review Committee prior to data collection. The ethical approval reference no: SLIIT/ERC/SBS/2025/20 grants permission to conduct this study in all phases including participant recruitment and data analysis. In line with the institution’s ethics compliance process, all interview transcripts have been submitted to the Ethics Review committee head, Professor Wasantha Rajapaksha.* *Hence, all transcripts have been deposited in the institutional repository. Additionally, all participants interviewed were thoroughly informed regarding details surrounding the study’s purpose, contribution, data usage, and the voluntary right to withdraw participation during any stage of the interview. Consent was obtained from all participants through a verbal reaffirmation of their rights prior to conducting each interview session whereby each verbal consent was recorded.”* |
| 4. If the reviewer comments include a recommendation to cite specific previously published works, please review and evaluate these publications to determine whether they are relevant and should be cited. There is no requirement to cite these works unless the editor has indicated otherwise. | Thank you very much for your valuable feedback. Any work cited has been double checked for relevancy and applicability. |

| **Reviewer’s Comments** | **Response to the Comment** |
| --- | --- |
| **1. The role of digital skills is not properly integrated into the main contribution**  The first research question and the abstract place digital skills at the centre of the study. The manuscript argues that digital skills shape motivation and engagement. Yet, once the discussion introduces the motivation interplay matrix and typology, digital skills largely disappear from the main framework. This creates a serious conceptual problem. The paper begins as a study of digital skills, motivation, and engagement, but the main contribution becomes a typology of intrinsic and extrinsic motivation without showing exactly how digital skills influence the typology. The authors need to revise the conceptual model so that digital skills are clearly positioned either as an antecedent, a mechanism, or a contextual condition. For example, digital skills could be theorised as strengthening perceived competence, which may then turn client feedback or payment into a reinforcing rather than controlling force. This link needs to be demonstrated with data, not only stated conceptually. | Thank you very much for bringing this to our attention.  We agree that the original manuscript contained a conceptual disconnection between the digital skills argument established in the findings and the motivation interplay matrix introduced in the discussion. This has been fully addressed by explicitly theorising digital skills as an antecedent mechanism that shapes the motivational experience a worker is deemed to have. In response to your insightful suggestion, a subheading *“5.1.1 Digital skills as an antecedent mechanism”* has been incorporated to thoroughly establish this mechanism. Kindly refer to line **(Line 939 – Line 979)** in the revised manuscript.  *“5.1.1 Digital skills as an antecedent mechanism*  *Prior research often characterises digital skills as a technical capability or key labour market asset that is crucial to build resilience in digital platforms [13]. However, the current findings offer a novel perspective by demonstrating its function as an antecedent mechanism that shapes the way gig workers experience motivation. Gig workers with advanced proficiencies recall their motivational experiences being characterised by confidence, creativity, and professional growth. Whereas gig workers who recognised skill gaps reflected on how skill deficiencies constrained potential motivational rewards. The findings of this study substantiate the antecedent positioning of digital skills through three particular sub-mechanisms present across multiple participant accounts.*  *The first sub-mechanism is competence enablement whereby digital skills emerged as a critical factor that cultivates perceptions of competence and influences whether external forces are experienced as an affirmation or a threat. According to SDT theory, perceived competence serves as a critical element that drives autonomous motivation [27]. Consequently, the current findings demonstrate that engaging in gig work alone does not guarantee motivational rewards as proficient digital skills serve as the structural condition whereby perceptions of competence are developed. Participant 5 reflects on this sentiment by emphasising how a skill deficiency in video editing prevented them from taking on more gigs, which hindered both income and competence satisfaction from completing tasks. Similarly, participant 1 contributes to this discourse by sharing a similar view from the opposite direction whereby the gig workers’ strong art skills played a critical role in attracting clients. In such instances, strong digital skills established the condition for recurrent and motivationally rewarding gig interactions.*  *Moreover, cognitive liberation emerged as the second sub-mechanism referring to the motivational condition that arises when digital skills exhibit exceptional proficiency to the point of automaticity, thereby minimising cognitive load and directing it towards intrinsic dimensions of gig work. Hence, cognitive liberation determines the depth and quality of motivational rewards once the competence threshold has been met. For instance, participant 5’s account of digital tool mastery reflects on how strong proficiencies aided in producing muscle memory which ultimately contributed to a more efficient output. This indicates that when technical execution requires minimal attention, the motivational space of the gig interaction expands allowing gig workers to experience the intrinsic components of completing the task.*  *Motivational recursion emerged as the third sub-mechanism referring to the awareness of skill development in influencing the overall motivational experience. This awareness encourages persistent, self-directed skill development that amplifies motivation overtime. Participant 14 highlights this sub-dimension by reflecting on the significance of proactive skill development to meet various client needs rather than as an external requirement. Each client presents a new learning opportunity that enhances skill growth and ultimately contributes to a more rewarding future engagement. Similarly, participant 8’s account of recognising that stronger skills would enable a more efficient task completion connects the anticipated efficiency to a deeper motivational experience. Hence, skill development progressively shifts gig workers towards an autonomous motivational experience overtime.”* |
| **2. The motivation matrix and typology are not yet sufficiently grounded in the data**  The proposed quadrants and profiles are the most important contribution of the manuscript, but they are not adequately supported by the results section or the supplementary thematic maps. The maps list broad themes such as financial rewards, client feedback, autonomy, competence, relatedness, and skill development. They do not show codes that capture the interaction between intrinsic and extrinsic motivation, nor do they show how the dimensions “reinforcing” and “controlling” were derived. The authors should return to the interview data and conduct a more relational analysis. They need to identify interaction codes, such as rewards being experienced as competence affirmation, client ratings being experienced as pressure, or skill enjoyment reducing dependence on external validation. The revised paper should include a table linking each quadrant or profile to specific participants and direct quotations. If there is not enough evidence for one of the quadrants, especially the controlling dimension, the framework should be revised rather than forced onto the data. | Thank you very much for this detailed and constructive critique.  The revised manuscript addresses this comprehensively through a complete reanalysis of the interview transcripts. The data analysis process consisted of two phases whereby the initial phase consists of the Nvivo coding which was utilised to identify recurring patterns in the data as supported by supplementary material S1. The next phase consisted of a manual two cycle coding process to gain deeper insights into the interactions between the variables, supported by the supplementary material S2.  Furthermore, A table linking participant accounts to each matrix quadrant has been incorporated in the discussion section (**Table 3**).  Additionally, the results section has been amended to adequately support the Motivation interplay matrix and typology. Kindly refer to **(line 346 – line 576)** in the revised manuscript or the heading *4. Results.* |
| **3. The qualitative analysis needs more transparency and methodological detail**  The methods section currently provides only a general description of thematic analysis. It is not clear who coded the transcripts, whether a codebook was developed, whether coding was inductive, deductive, or abductive, how disagreements were resolved, and whether any form of peer checking, reflexive memoing, or audit trail was used. The use of NVivo does not itself demonstrate analytical rigour. The authors should provide a clear account of the coding process, including how raw interview material became codes, sub-themes, themes, and then the final typology. The supplementary file should be rebuilt as a proper codebook or thematic evidence table rather than a set of simple diagrams. | Thank you very much for bringing this to our attention.  The supplementary file has been revised to provide the Nvivo Codebook alongside the manual codebook. Kindly refer to the documents **S1** and **S2.**  The data analysis section (*3.4 Data analysis*) has been revised to provide a detailed account of the coding process followed. Kindly refer to **(line 309 – line 333)** in the revised manuscript  *“3.4 Data analysis*  *Data analysis was conducted thematically [52] and was carried out in two complementary phases combining computer-assisted and manual techniques to strengthen the rigour and transparency of the process [53]. In the first phase, all interview transcripts were imported into NVivo and coded to identify recurring ideas and viewpoints illustrated by the participants. Through this, all authors worked simultaneously to ensure similar codes were grouped which formed four broad themes that captured the most prominent patterns across the dataset as supported S1. As these themes were relatively broad and did not fully* *capture the nuances within participant’s expressions, therefore, a second manual phase was commended using a spreadsheet by the main author. This enabled key quotes reflecting the primary themes to be organised and compared to surface more detailed patterns. This focused analysis refined the broad themes and led to the generation of first cycle codes which summarised the data alongside causation codes to causal relationships expressed by participants. Next, second cycle pattern codes were generated by grouping the first cycle codes into analytical labels that captured the relational configurations across participant accounts [54]. Hence, the causation codes developed served as the interaction codes through which relational patterns were observed between digital skills, motivation, and engagement as supported S2. This dual approach achieves the most robust* *results [53,55], with NVivo supporting efficient data management and manual analysis enabling deeper, more rigorous interpretation. Figure 1 illustrates the data analysis process with clarity*.” |
| **4. The claim of saturation is not adequately justified**  The manuscript states that saturation was reached around the twelfth interview, but no evidence is provided. This point matters because the sample is small, and the saturation claim is used to justify it. The authors should provide a brief saturation table showing when major codes first appeared and whether interviews 13 and 14 added new codes or only confirmed existing ones. They should also clarify whether they mean code saturation, meaning saturation, or theoretical saturation. If the study is not grounded theory, the phrase “theoretical saturation” should be used more carefully. | Thank you very much for this methodologically precise observation. Taking this comment into account, a saturation table has been incorporated into the manuscript **(Table 1).** Additionally, the saturation claim has been amended, kindly refer to **(line 273 – line 292).**  “3*.2 Data collection method*  *Primary data was collected through 14 in-depth semi-structured interviews with open-ended questions spanning the course of two weeks, with an average session being approximately 28.4 minutes. The interviews were conducted by two researchers at a time and participants were asked broad open-ended questions that focused on skill acquisition, factors that motivated them to engage in gig work, the influence of digital skills on participation in gig work, and challenges incurred while working on digital labour platforms. Through the interview series, the researchers discovered participants’ perceptions and experiences working in online freelancing platforms. During this stage, interviews were continuously reviewed to identify the emergence of new codes and themes. Interview process was concluded when code saturation was reached approximately at the 12th interview as code generation progressively declined. Table 1 illustrates the saturation table, whereby participant 12 generates only one new code (2.22%) indicating near saturation and aligning with [50] ≤5% threshold. Hence, interviews 13 and 14 were conducted to primarily strengthen existing themes and contributed to thematic robustness [50]. All participants were assigned pseudonyms to safeguard their privacy. Interviews were conducted online through Microsoft Teams with permission granted from participants to record each session. Transcripts were generated using the tool Premiere Pro from Adobe Creative Suite which were further* *spot-checked by the authors for accuracy and clarification, to ensure transcript verification [51]. Table 2 provides an overview of the participants’ demographics and characteristics.”* |
| **5. The sample limits the typology more than the manuscript currently admits**  The sample consists of 14 participants, selected through convenience sampling, and appears to be concentrated among young platform workers in creative, technical, and digital service fields. Most participants are part-time workers. This is a serious limitation for a typology that includes an extrinsic-controlling profile, because that profile may be more visible among workers who depend on platform work as their primary income. The participant table should include whether platform work is a primary or secondary income source. The discussion and limitations should state clearly that the proposed typology may not capture the experiences of workers under high financial necessity, platform dependence, or algorithmic pressure. | Thank you for your valuable observations. Upon careful reexamination of the manuscript during the revision process, we acknowledge that the sampling approach has been incorrectly described as convenience sampling in the original manuscript. The approach used more accurately reflects snowball sampling where initial participants who met the inclusion criteria, suggest subsequent participants from their professional network. This sampling technique is particularly suitable for the purpose of this study as there is no documented consensus of digital gig workers in Sri Lanka.  However, in terms of participant characteristics, it is important to note that gig workers represent a relatively homogenous sample as they share common structural conditions [3,15]. Furthermore, according to [7] gig workers are predominantly young and urban based and engage in short-term flexible work across creative and professional industries in platforms such as Fiverr and Upwork. However, for the purpose of the Extrinsic Dominant quadrant, three specific changes have been made to amend the revised manuscript. First, the participant table (**Table 2**) has been revised to include a dedicated column to document whether gig workers engaged part time or full time in gig work. Second, the limitations section has been amended to acknowledge the limitation in sample size, demonstrated by **(line 1183 to line 1191).** Kindly refer to the manuscript excerpt:  *“Second, the sample consisted of predominantly young digital gig workers concentrated in fields such as creative and technical platform work, with a large majority engaging in gig work as a part-time source of income. Therefore, the participant accounts may largely reflect the experiences of gig workers who possess greater occupational autonomy and flexibility than gig workers who exclusively depend on the platform as their primary source of income. This serves as a limitation particularly for the extrinsic-controlling motivational profile which captures the experiences of gig workers under high financial necessity. Hence, future research can offer deeper insights into this motivational profile by examining whether the current findings are replicated among workers with high platform dependence or algorithmic pressure.”*  Third, this limitation has also been briefly acknowledged in the discussion of this quadrant **(line 1047– line 1048).**  *“The relative scarcity of this motivational profile reflects a limitation in sample size rather than a rarity of the condition itself in gig work.”* |
| **6. The concept of engagement is underdeveloped**  The manuscript uses “engagement” as a central outcome, but the term is not defined precisely enough. Sometimes it appears to mean continued platform participation; sometimes work commitment; sometimes creative involvement; and sometimes satisfaction with platform support. The authors need to distinguish which form of engagement they are analysing: behavioural engagement, emotional engagement, cognitive engagement, platform continuance, work intensity, or another construct. Without this clarification, the link between motivation and engagement remains too broad. | Thank you for this valuable observation. We agree that the concept of engagement was insufficiently specified in the original manuscript, which may have created ambiguity regarding the outcome examined in the study. In response, we have substantially revised the manuscript to provide a clearer conceptualisation of engagement.  These changes are reflected through the sub heading *2.4 Conceptualising engagement within digital gig work* located in **(line 227 to line 249)** reflected in the excerpt below:  **“***2.4 Conceptualising engagement within digital gig work*  *Engagement is a multidimensional construct that has been conceptualised through behavioural, cognitive, and emotional dimensions, reflecting the extent to which individuals invest their effort, attention, and psychological resources in work-related activities [44,45]. However, in digitally mediated labour markets, engagement extends beyond psychological attachment and is increasingly reflected in workers’ continued participation within platforms. Prior research suggests that the sustainability of digital labour platforms depends not merely on workers’ temporary involvement but on their continuous and uninterrupted participation over time [29]. Similarly, gig economy scholars argue that engagement is reflected through workers’ willingness to remain active, repeatedly accept work opportunities, and sustain their involvement despite the inherent uncertainties of platform-based employment [3,43]. Drawing on this perspective, the present study conceptualises engagement as continuance engagement, defined as the extent to which gig workers sustain their participation in digital labour platforms through continued acceptance of projects, ongoing involvement in gig activities, and persistence in platform-based work overtime. This conceptualisation is particularly appropriate given that participation in gig work is largely voluntary and influenced by workers’ motivational experiences. Therefore, while factors such as creative autonomy, work commitment, client relationships, and satisfaction with platform support emerged during the interviews, these are interpreted as antecedents that facilitate engagement rather than engagement itself. Accordingly, this study focuses specifically on the behavioural outcome of sustained platform participation rather than emotional attachment, cognitive absorption, job satisfaction, or organisational commitment, thereby examining how intrinsic and extrinsic motivational forces contribute to the continued participation of gig workers within the digital gig economy.”* |
| **7. The theoretical contribution is overstated**  The manuscript claims that the matrix extends Self-Determination Theory. This is too strong for the evidence currently provided. The idea that intrinsic and extrinsic motivation can coexist is already well established in the motivation literature. A more defensible contribution would be to say that the study applies or contextualises SDT in the setting of Sri Lankan platform-based freelancers and explores how digital skills may support competence, autonomy, and internalisation. The authors should also compare their typology with previous classifications of gig worker motivation, such as the “dabblers” discussion and the work of Jabagi et al., to show whether their profiles are genuinely new or mainly a re-labelling of existing categories. | Thank you for this valuable observation. The theoretical implications have been revised to reflect the study’s contributions as a contextualisation of SDT theory rather than an extension. This is reflected in **(line 1110 – line 1112)** and demonstrated in the excerpt below:  *“First, the motivation interplay matrix contextualises Self-Determination Theory [27] within the platform-based gig economy of Sri Lanka by demonstrating the role of digital skills in shaping the interaction between intrinsic and extrinsic motivation.”*  The typology has also been compared with previous work by Jabagi et al., and Dunn (2020). Kindly refer to **(line 1136 – 1142)** and **(line 1121 – line 1127)**  **(line 1136 – 1142)**  *“The four motivational profiles share similarities with [32] five category typology with the most relevant comparison being between the “Dabbler” profile and intrinsic dominant profile in the current study. While “Dabblers” share similarities with intrinsic dominant gig workers in terms of being less financially precarious, the current study reveals that intrinsic dominant workers are heavily governed by passion, creative autonomy, and well-being orientation which serves as a contrast to the passive non-attachment that governs “Dabblers”.”*  **(line 1121 – line 1127)**  *“In fact, this study responds to the suggestion of [16] who stated that “future work exploring motivation within the gig-economy should consider the dynamics between these types of motivation”, referring to intrinsic and extrinsic motivation. Therefore, the motivation interplay matrix provides fresh insights about the dynamic interplay between intrinsic and extrinsic motivation, demonstrating that both forces can coexist through mutual reinforcement or control one another’s presence.”* |
| **8. The supplementary thematic maps need substantial revision**  The current supplementary material is not sufficient to support the qualitative claims. The maps are descriptive, repetitive, and do not show how the final typology was produced. The authors should replace them with a more useful supplementary file that includes code definitions, sub-themes, representative quotations, participant counts or coverage, and analytic notes. A participant-by-theme matrix would also help readers judge whether the typology is supported across cases or only by a few isolated quotations. | Thank you for this valuable insight. The revised manuscript replaces the thematic maps with two supplementary files to make the data underlying the study freely accessible. S1 contains an Nvivo codebook documenting the first stage of the coding process. S2 contains a manual codebook documenting the major codes developed. |
| **9. Data availability must be corrected**  The current statement that data are available upon request from the corresponding author is unlikely to be sufficient unless there is a clear ethical or legal restriction. The authors should either provide de-identified transcripts, relevant transcript excerpts, the interview guide, and the codebook as supporting information, or explain the restriction clearly and provide an institutional route for data access. This is especially important because the empirical contribution depends heavily on qualitative evidence. | Thank you very much for your valuable feedback. In line with PLOS journal policy, two supplementary files have been uploaded to make the data underlying the study freely accessible. S1 contains an Nvivo codebook documenting the first stage of the coding process. S2 contains a manual codebook documenting the major codes developed. Accordingly, the title page has been amended to reflect this change.  Ethical approval was secured from the Sri Lanka Institute of Information Technology Ethics Review Committee prior to commencing the data collection. Next, verbal consent was established prior to commencing the interviews and was audio recorded alongside the interview itself. Hence, the interview transcripts also document the consent obtained. Additionally, the relevant transcripts have been submitted to the institute’s Ethics Review Committee Head, Professor Wasantha Rajapaksha and deposited in the institutions data repository,  This comment has been addressed in the revised manuscript as follows **(Line 334 -345)**:  *“This study rigorously adhered to established ethical standards surrounding primary research that incorporates human participants. An ethical approval was secured from Sri Lanka Institute of Information Technology Research Ethics Review Committee prior to data collection. The ethical approval reference no: SLIIT/ERC/SBS/2025/20 grants permission to conduct this study in all phases including participant recruitment and data analysis. In line with the institution’s ethics compliance process, all interview transcripts have been submitted to the Ethics Review Committee head, Professor Wasantha Rajapaksha.* *Hence, all transcripts have been deposited in the institutional repository. Additionally, all participants interviewed were thoroughly informed regarding details surrounding the study’s purpose, contribution, data usage, and the voluntary right to withdraw participation during any stage of the interview. Consent was obtained from all participants through a verbal reaffirmation of their rights prior to conducting each interview session whereby each verbal consent was recorded.”* |
| **10. Presentation and structure require careful editing**  The manuscript also needs close editing. The results section should be reordered logically, section numbering should be corrected, and the figure and tables should be improved. Several references appear incomplete or inconsistently formatted. The language is generally understandable, but there are many sentences that need tightening for clarity and academic tone. These issues are secondary to the methodological concerns, but they should still be addressed before the manuscript is reconsidered. | Thank you very much for bringing this to our attention.  In line with the feedback, we have revised the heading numbering, references, tables, and improved language for clarity. |
| **11. The literature review and reference base need further strengthening**  The literature review should be strengthened with more recent and higher-quality references. Although the manuscript cites several studies on gig work, digital skills, and motivation, it does not sufficiently position the study within recent debates in **PLOS ONE** or other leading journals. Notably, the current reference list does not appear to include relevant studies from the target journal itself, despite the manuscript being submitted to **PLOS ONE** and claiming a novel contribution on digital work, motivation, and worker engagement. The authors should incorporate relevant PLOS ONE studies where appropriate, together with stronger recent literature on digitalization, platform work, worker well-being, motivation, and sustainable development. The SDG-related argument should also be supported more carefully, rather than being mentioned only as a broad implication. Relevant sustainability-oriented references may be considered, such as Abusamra and Chunmei (2026) and Yu et al. (2025), but these should be integrated only where they directly support the manuscript’s discussion of sustainable digital work, inclusive economic participation, or SDG-based policy framing. The reference list should be revised not by adding citations mechanically, but by using the literature to clarify the manuscript’s originality, theoretical positioning, and contribution.  Suggested references to consider:  Abusamra, O. A., & Chunmei, Z. (2026). Exploring the volatility dynamics and market interactions in sustainable finance products. Applied Economics, 1–17. <https://doi.org/10.1080/00036846.2026.2642886>  Yu, P., Abusamra, O. A., Usman, M., & Makhdum, M. S. A. (2025). Leveraging technological innovation, renewable energy, and forest management to overcome barriers and achieve Sustainable Development Goal 13 (Climate Action) in G-7 countries. Sustainable Development, 33(6), 8821–8839. <https://doi.org/10.1002/sd.70127> | Thank you very much for your valuable feedback. To adhere to the comments, several new references have been incorporated to the literature review. Kindly refer to **(line 149 to line 154), (199 – 205),** and **(214- 219)** in the revised manuscript.  **(line 149 to line 154)**  *“[26] evidence strengthens this broader view by showing that digital competence is not limited to basic technical ability but reflects a multidimensional capability needed to function effectively in digital environments. In particular, in this study digital transformation skills identifies skillsets such as digital work, communication, collaboration, adaptation, and evidence-based work as core components of digital competence, which supports the argument that gig workers need more than operational platform knowledge to succeed in digitally mediated work*.”  **(199 – 205)**  *“Recent empirical evidence also suggests that payment design in gig work does not operate in isolation, because workers’ responses to incentives are shaped by their intrinsic task motivation and perceptions of control. In a field experiment on online freelancers, [39] found that a payment scheme combining commission with a fixed component reduced performance among less intrinsically motivated workers but improved performance among highly intrinsically motivated workers, showing that extrinsic incentives can have different effects depending on worker motivation profiles.”*  **(214- 219)**  *“Gamified recognition should also be interpreted cautiously, because feedback systems may function as both motivational support and behavioural control in digital labour settings. When platform feedback is tied to visibility, ranking, or access to future work, recognition can reinforce engagement while simultaneously increasing pressure to conform to platform expectations. This makes praise and recognition a potentially mixed extrinsic driver rather than a uniformly positive one [43]”* |

| **Overall Comments** | **Response to the Comment** |
| --- | --- |
| 1.Introduction: a) It would be helpful if the authors briefly described the types of labor typically employed on platform-based gig work sites like Fiverr and Upwork. b) The phrasing of the study's research questions are written in such a way that a quantitative research design would more aptly answer them. Consider re-phrasing them to be more open-ended and centered on capturing themes of lived human experience, rather than an investigation of the relationships between operationalized variables. | Thank you for this valuable feedback.  a) The various types of labour undertaken in platform-based gig work has been briefly incorporated in the introduction. Kindly refer to (**line 68 – line 69)** and the following excerpt:  *“The most commonly undertaken gigs in platform-based gig work ranges from graphic design, web development, content writing, and project-based gig work [7]”*  b) We have taken this feedback into account and amended the research question 1 as follows:  *“1) How do gig workers describe the role of digital skills in shaping motivational experiences in digital gig platforms?”* |
| Population and sample: a) The sampling of acquaintances is a major methodological concern that is not sufficiently addressed. The explanation offered ("Despite the population being known, the absence of the count of digital gig workers in Sri Lanka led to the application of a non-probability approach enabling the researcher to offer credible and meaningful results [44]") does not meaningfully justify this methodological decision; moreover, the citation offered does not make any supporting claims of this methodological decision as it restricts its argument to quantitative research. The authors must discuss steps taken to ensure bias was accounted for in both the interview and the data analysis processes. | We thank the reviewer for this valuable observation. Upon careful reexamination of the manuscript during the revision process, we acknowledge that the sampling approach has been incorrectly described as convenience sampling in the original manuscript. The approach used more accurately reflects snowball sampling where initial participants who met the inclusion criteria, suggest subsequent participants from their professional network. This sampling technique is particularly suitable for the purpose of this study as there is no documented consensus of digital gig workers in Sri Lanka.  This has been amended in the manuscript under the heading *3.1 Population and sample* present in **(Line 256- Line 272):**  *“The population considered were full-time and part-time digital gig workers in Sri Lanka above the age of 18. The participants were recruited using snowball sampling whereby initial participants who met the inclusion criteria later recommended the subsequent participants [47]. This sampling technique is methodologically appropriate in situations where the target population is difficult to reach through conventional sampling techniques [48]. The absence of a* *standard count of digital gig workers in Sri Lanka led to the application of a non-probability approach enabling the researcher to offer credible and meaningful results [49].”* |
| 3.2 Data collection method: a) Please share more details about who conducted the interviews. Was it just one of the researchers, multiple, or other people entirely? b) Please describe which core open-ended questions were asked during the interviews. | Thank you for this valuable comment. The interviews were conducted by the authors with two authors actively participating to conduct the interviews. This has been addressed in section *3.2 Data collection method* through (**line 276 – line 279**). The revised manuscript excerpt is as follows:  “*The interviews were conducted by two researchers at a time and participants were asked broad open-ended questions that focused on skill acquisition, factors that motivated them to engage in gig work, the influence of digital skills on participation in gig work, and challenges incurred while working on digital labour platforms.”* |
| 3.3 Participants: a) Participants are skewed almost exclusively male and emerging adult, with the jobs exclusively relying on STEM-based knowledge. This severely limits the scope of the themes the authors are developing from the data. Please acknowledge this, either in this section or in the limitations. b) It is unclear if gig work is participants' sole job. If data is available describing participants' primary employment, consider reporting it. | Thank you very much for bringing this to our attention.  In terms of participant characteristics, it is important to note that gig workers represent a relatively homogenous sample as they share common structural conditions [3,15]. However, for the purpose of the Extrinsic Dominant quadrant, three specific changes have been made to amend the revised manuscript. First, the participant table **(Table 2)** has been revised to include a dedicated column to document whether gig workers engaged part time or full time in gig work. Second, the limitations section has been amended to acknowledge the limitation in sample size for this quadrant, demonstrated by (line 1183 to line 1191). Kindly refer to the manuscript excerpt:  *“Second, the sample consisted of predominantly young digital gig workers concentrated in fields such as creative and technical platform work, with a large majority engaging in gig work as a part-time source of income. Therefore, the participant accounts may largely reflect the experiences of gig workers who possess greater occupational autonomy and flexibility than gig workers who exclusively depend on the platform as their primary source of income. This serves as a limitation particularly for the extrinsic-controlling motivational profile which captures the experiences of gig workers under high financial necessity. Hence, future research can offer deeper insights into this motivational profile by examining whether the current findings are replicated among workers with high platform dependence or algorithmic pressure”*  Third, this limitation has also been briefly acknowledged in the discussion of this quadrant (line 1047– line 1048).  *“The relative scarcity of this motivational profile reflects a limitation in sample size rather than a rarity of the condition itself in gig work.”* |
| 3.4 Data analysis: a) While NVivo is an appropriate tool for qualitative analysis, the authors must still expand on their a priori frameworks and epistemological assumptions going into the analysis. Since thematic analysis is a (mostly) ideologically independent analytic framework, it is thus important for the authors to note their own lived experiences, personal investments, or ideological presuppositions with regard to the topic of research and the data at hand via some researcher reflexivity or positionally subsection, since these elements are unavoidably omnipresent throughout the qualitative research process. Moreover, the authors must make more clear the steps taken in analyzing the data for themes by ensuring each step is included and describing what each step entailed. The authors must also report on if one researcher analyzed the data, or if multiple researchers collaborated to develop themes (in which case, a statistical test for inter-rater reliability would be necessary). | Thank you for this valuable feedback. The data analysis section *(3.4 Data analysis)* has been revised to provide a detailed account of the coding process followed. Kindly refer to (line 309 – line 333) in the revised manuscript  *“3.4 Data analysis*  *Data analysis was conducted thematically [52] and was carried out in two complementary phases combining computer-assisted and manual techniques to strengthen the rigour and transparency of the process [53]. In the first phase, all interview transcripts were imported into NVivo and coded to identify recurring ideas and viewpoints illustrated by the participants. Through this, all authors worked simultaneously to ensure similar codes were grouped which formed four broad themes that captured the most prominent patterns across the dataset as supported S1. As these themes were relatively broad and did not fully capture the nuances within participant’s expressions, therefore, a second manual phase was commended using a spreadsheet by the main author. This enabled key quotes reflecting the primary themes to be organised and compared to surface more detailed patterns. This focused analysis refined the broad themes and led to the generation of first cycle codes which summarised the data alongside causation codes to causal relationships expressed by participants. Next, second cycle pattern codes were generated by grouping the first cycle codes into analytical labels that captured the relational configurations across participant accounts [54]. Hence, the causation codes developed served as the interaction codes through which relational patterns were observed between digital skills, motivation, and engagement as supported S2. This dual approach achieves the most robust results [53,55], with NVivo supporting efficient data management and manual analysis enabling deeper, more rigorous interpretation. Figure 1 illustrates the data analysis process with clarity.”* |
| 4 Results: a) Overall, please ensure that all claims remain within the scope of what your data can explain. Some interpretations of the data go beyond what the excerpts provided seem to suggest. b) Please ensure all sections are where they belong. It looks like 4.2 got moved below 4.4. | Thank you for your valuable insights. The theoretical implications have been revised to reflect the study’s contributions as a contextualisation of SDT theory rather than an extension. This is reflected in (line 1111 – line 1114) and demonstrated in the excerpt below:  *“First, the motivation interplay matrix contextualises Self-Determination Theory [27] within the platform-based gig economy of Sri Lanka by demonstrating the role of digital skills in shaping the interaction between intrinsic and extrinsic motivation.”*  Additionally, the section headings have been revised with formatting precision. |
| 5.1 Mapping intrinsic and extrinsic drivers in a Motivation Interplay Matrix: a) While there may be a strong theoretical basis for this model based on previous literature, the excerpts provided do not sufficiently illustrate such a model. It also does not seem appropriate to use data that is not necessarily generalizable as the basis for a generalizable theoretical model. As it stands, this matrix may be more usefully deemed beyond the scope of the present paper, and may be more strong as a standalone piece. | Thank you for this valuable observation. While we do agree that the original manuscript did not sufficiently illustrate this model, The revised manuscript addresses this comprehensively through a complete reanalysis of the interview transcripts.  The data analysis process consisted of two phases whereby the initial phase consists of the Nvivo coding which was utilised to identify recurring patterns in the data as supported by supplementary material 2. The next phase consisted of a manual two cycle coding process to gain deeper insights into the interactions between the variables, supported by Supplementary material 1.  Furthermore, A table linking participant accounts to each matrix quadrant has been incorporated in the discussion section (**Table 3**).  Additionally, the results section has been amended to adequately support the Motivation interplay matrix and typology. Kindly refer to **(line 346 – line 576)** in the revised manuscript or the heading *4. Results.* |
| 7. Conclusion and limitations: a) Based on the feedback provided, please ensure the study's limitations are expanded to include theoretical and methodological concerns. | Thank you for your insightful comment. We have addressed the applicable limitations accordingly. The limitations section has been amended to acknowledge the limitation in sample size, demonstrated by **(line 1184 to line 1192)**. Kindly refer to the manuscript excerpt:  *“Second, the sample consisted of predominantly young digital gig workers concentrated in fields such as creative and technical platform work, with a large majority engaging in gig work as a part-time source of income. Therefore, the participant accounts may largely reflect the experiences of gig workers who possess greater occupational autonomy and flexibility than gig workers who exclusively depend on the platform as their primary source of income. This serves as a limitation particularly for the extrinsic-controlling motivational profile which captures the experiences of gig workers under high financial necessity. Hence, future research can offer deeper insights into this motivational profile by examining whether the current findings are replicated among workers with high platform dependence or algorithmic pressure”* |
