# Supplementary material for: What keeps gig workers motivated? A typology of the intrinsic-extrinsic motivation interplay in the digital gig economy
Source: PLoS One. 2026 Sep 22;21(9):e0345881. doi: 10.1371/journal.pone.0345881 (PMC13596809; doi:10.1371/journal.pone.0345881)
Supplement: Peer review history

# Peer review: Decision Letter 0

**DOI:** 10.1371/journal.pone.0345881.r001

**Authors:** Amruta Deshpande

13 May 2026

PONE-D-26-12283

What Keeps Gig Workers Motivated? A Typology of the Intrinsic-Extrinsic Motivation Interplay in the Digital Gig Economy

PLOS One?

Dear Dr. Wisenthige,

Please submit your revised manuscript by Jun 27 2026 11:59PM. If you will need more time than this to complete your revisions, please reply to this message or contact the journal office at plosone@plos.org. When you're ready to submit your revision, log on to https://www.editorialmanager.com/pone/ and select the 'Submissions Needing Revision' folder to locate your manuscript file.

- A letter that responds to each point raised by the academic editor and reviewer(s). You should upload this letter as a separate file labeled 'Response to Reviewers'.
- A marked-up copy of your manuscript that highlights changes made to the original version. You should upload this as a separate file labeled 'Revised Manuscript with Track Changes'.
- An unmarked version of your revised paper without tracked changes. You should upload this as a separate file labeled 'Manuscript'.

If you would like to make changes to your financial disclosure, please include your updated statement in your cover letter. Guidelines for resubmitting your figure files are available below the reviewer comments at the end of this letter.

If applicable, we recommend that you deposit your laboratory protocols in protocols.io to enhance the reproducibility of your results. Protocols.io assigns your protocol its own identifier (DOI) so that it can be cited independently in the future. For instructions see: https://journals.plos.org/plosone/s/submission-guidelines#loc-laboratory-protocols. Additionally, PLOS ONE offers an option for publishing peer-reviewed Lab Protocol articles, which describe protocols hosted on protocols.io. Read more information on sharing protocols at https://plos.org/protocols?utm_medium=editorial-email&utm_source=authorletters&utm_campaign=protocols.

As the corresponding author, your ORCID iD is verified in the submission system and will appear in the published article. PLOS supports the use of ORCID, and we encourage all coauthors to register for an ORCID iD and use it as well. Please encourage your coauthors to verify their ORCID iD within the submission system before final acceptance, as unverified ORCID iDs will not appear in the published article. *Only*  the individual author can complete the verification step; PLOS staff *cannot*  verify ORCID iDs on behalf of authors.

We look forward to receiving your revised manuscript.

Kind regards,

Amruta Deshpande

Academic Editor

PLOS One

Journal Requirements:

When submitting your revision, we need you to address these additional requirements.

1. Please ensure that your manuscript meets PLOS ONE's style requirements, including those for file naming. The PLOS ONE style templates can be found at

https://journals.plos.org/plosone/s/file?id=wjVg/PLOSOne_formatting_sample_main_body.pdf and

https://journals.plos.org/plosone/s/file?id=ba62/PLOSOne_formatting_sample_title_authors_affiliations.pdf

2. In the ethics statement in the Methods, you have specified that verbal consent was obtained. Please provide additional details regarding how this consent was documented and witnessed, and state whether this was approved by the IRB.

3. In the online submission form, you indicated that the data underlying the results presented in the study are available upon a written request to corresponding author.

All PLOS journals now require all data underlying the findings described in their manuscript to be freely available to other researchers, either 1. In a public repository, 2. Within the manuscript itself, or 3. Uploaded as supplementary information.

This policy applies to all data except where public deposition would breach compliance with the protocol approved by your research ethics board. If your data cannot be made publicly available for ethical or legal reasons (e.g., public availability would compromise patient privacy), please explain your reasons on resubmission and your exemption request will be escalated for approval.

4. If the reviewer comments include a recommendation to cite specific previously published works, please review and evaluate these publications to determine whether they are relevant and should be cited. There is no requirement to cite these works unless the editor has indicated otherwise.

Additional Editor Comments:

We invite authors to submit revised manuscript

[Note: HTML markup is below. Please do not edit.]

Reviewers' comments:

Reviewer's Responses to Questions

**Comments to the Author**

1. Is the manuscript technically sound, and do the data support the conclusions?

Reviewer #1: Yes

Reviewer #2: Partly

Reviewer #3: Yes

**********

2. Has the statistical analysis been performed appropriately and rigorously?

Reviewer #1: No

Reviewer #2: N/A

Reviewer #3: No

**********

3. Have the authors made all data underlying the findings in their manuscript fully available?

The PLOS Data policy

Reviewer #1: No

Reviewer #2: Yes

Reviewer #3: No

**********

4. Is the manuscript presented in an intelligible fashion and written in standard English?

Reviewer #1: No

Reviewer #2: Yes

Reviewer #3: Yes

**********

Reviewer #1: Major comments

1. The role of digital skills is not properly integrated into the main contribution

The first research question and the abstract place digital skills at the centre of the study. The manuscript argues that digital skills shape motivation and engagement. Yet, once the discussion introduces the motivation interplay matrix and typology, digital skills largely disappear from the main framework. This creates a serious conceptual problem. The paper begins as a study of digital skills, motivation, and engagement, but the main contribution becomes a typology of intrinsic and extrinsic motivation without showing exactly how digital skills influence the typology. The authors need to revise the conceptual model so that digital skills are clearly positioned either as an antecedent, a mechanism, or a contextual condition. For example, digital skills could be theorised as strengthening perceived competence, which may then turn client feedback or payment into a reinforcing rather than controlling force. This link needs to be demonstrated with data, not only stated conceptually.

2. The motivation matrix and typology are not yet sufficiently grounded in the data

The proposed quadrants and profiles are the most important contribution of the manuscript, but they are not adequately supported by the results section or the supplementary thematic maps. The maps list broad themes such as financial rewards, client feedback, autonomy, competence, relatedness, and skill development. They do not show codes that capture the interaction between intrinsic and extrinsic motivation, nor do they show how the dimensions “reinforcing” and “controlling” were derived. The authors should return to the interview data and conduct a more relational analysis. They need to identify interaction codes, such as rewards being experienced as competence affirmation, client ratings being experienced as pressure, or skill enjoyment reducing dependence on external validation. The revised paper should include a table linking each quadrant or profile to specific participants and direct quotations. If there is not enough evidence for one of the quadrants, especially the controlling dimension, the framework should be revised rather than forced onto the data.

3. The qualitative analysis needs more transparency and methodological detail

The methods section currently provides only a general description of thematic analysis. It is not clear who coded the transcripts, whether a codebook was developed, whether coding was inductive, deductive, or abductive, how disagreements were resolved, and whether any form of peer checking, reflexive memoing, or audit trail was used. The use of NVivo does not itself demonstrate analytical rigour. The authors should provide a clear account of the coding process, including how raw interview material became codes, sub-themes, themes, and then the final typology. The supplementary file should be rebuilt as a proper codebook or thematic evidence table rather than a set of simple diagrams.

4. The claim of saturation is not adequately justified

The manuscript states that saturation was reached around the twelfth interview, but no evidence is provided. This point matters because the sample is small and the saturation claim is used to justify it. The authors should provide a brief saturation table showing when major codes first appeared and whether interviews 13 and 14 added new codes or only confirmed existing ones. They should also clarify whether they mean code saturation, meaning saturation, or theoretical saturation. If the study is not grounded theory, the phrase “theoretical saturation” should be used more carefully.

5. The sample limits the typology more than the manuscript currently admits

The sample consists of 14 participants, selected through convenience sampling, and appears to be concentrated among young platform workers in creative, technical, and digital service fields. Most participants are part-time workers. This is a serious limitation for a typology that includes an extrinsic-controlling profile, because that profile may be more visible among workers who depend on platform work as their primary income. The participant table should include whether platform work is a primary or secondary income source. The discussion and limitations should state clearly that the proposed typology may not capture the experiences of workers under high financial necessity, platform dependence, or algorithmic pressure.

6. The concept of engagement is underdeveloped

The manuscript uses “engagement” as a central outcome, but the term is not defined precisely enough. Sometimes it appears to mean continued platform participation; sometimes work commitment; sometimes creative involvement; and sometimes satisfaction with platform support. The authors need to distinguish which form of engagement they are analysing: behavioural engagement, emotional engagement, cognitive engagement, platform continuance, work intensity, or another construct. Without this clarification, the link between motivation and engagement remains too broad.

7. The theoretical contribution is overstated

The manuscript claims that the matrix extends Self-Determination Theory. This is too strong for the evidence currently provided. The idea that intrinsic and extrinsic motivation can coexist is already well established in the motivation literature. A more defensible contribution would be to say that the study applies or contextualises SDT in the setting of Sri Lankan platform-based freelancers and explores how digital skills may support competence, autonomy, and internalisation. The authors should also compare their typology with previous classifications of gig worker motivation, such as the “dabblers” discussion and the work of Jabagi et al., to show whether their profiles are genuinely new or mainly a re-labelling of existing categories.

8. The supplementary thematic maps need substantial revision

The current supplementary material is not sufficient to support the qualitative claims. The maps are descriptive, repetitive, and do not show how the final typology was produced. The authors should replace them with a more useful supplementary file that includes code definitions, sub-themes, representative quotations, participant counts or coverage, and analytic notes. A participant-by-theme matrix would also help readers judge whether the typology is supported across cases or only by a few isolated quotations.

9. Data availability must be corrected

The current statement that data are available upon request from the corresponding author is unlikely to be sufficient unless there is a clear ethical or legal restriction. The authors should either provide de-identified transcripts, relevant transcript excerpts, the interview guide, and the codebook as supporting information, or explain the restriction clearly and provide an institutional route for data access. This is especially important because the empirical contribution depends heavily on qualitative evidence.

10. Presentation and structure require careful editing

The manuscript also needs close editing. The results section should be reordered logically, section numbering should be corrected, and the figure and tables should be improved. Several references appear incomplete or inconsistently formatted. The language is generally understandable, but there are many sentences that need tightening for clarity and academic tone. These issues are secondary to the methodological concerns, but they should still be addressed before the manuscript is reconsidered.

11. The literature review and reference base need further strengthening

The literature review should be strengthened with more recent and higher-quality references. Although the manuscript cites several studies on gig work, digital skills, and motivation, it does not sufficiently position the study within recent debates in PLOS ONE or other leading journals. Notably, the current reference list does not appear to include relevant studies from the target journal itself, despite the manuscript being submitted to PLOS ONE and claiming a novel contribution on digital work, motivation, and worker engagement. The authors should incorporate relevant PLOS ONE studies where appropriate, together with stronger recent literature on digitalization, platform work, worker well-being, motivation, and sustainable development. The SDG-related argument should also be supported more carefully, rather than being mentioned only as a broad implication. Relevant sustainability-oriented references may be considered, such as Abusamra and Chunmei (2026) and Yu et al. (2025), but these should be integrated only where they directly support the manuscript’s discussion of sustainable digital work, inclusive economic participation, or SDG-based policy framing. The reference list should be revised not by adding citations mechanically, but by using the literature to clarify the manuscript’s originality, theoretical positioning, and contribution.

Suggested references to consider:

Abusamra, O. A., & Chunmei, Z. (2026). Exploring the volatility dynamics and market interactions in sustainable finance products. Applied Economics, 1–17. https://doi.org/10.1080/00036846.2026.2642886

Yu, P., Abusamra, O. A., Usman, M., & Makhdum, M. S. A. (2025). Leveraging technological innovation, renewable energy, and forest management to overcome barriers and achieve Sustainable Development Goal 13 (Climate Action) in G-7 countries. Sustainable Development, 33(6), 8821–8839. https://doi.org/10.1002/sd.70127

Reviewer #2: This manuscript discusses an important topic that, if published, may meaningfully improve the freelance work experience of others. First, issues primarily relating to methodology and interpretation of results need to be addressed before this paper can move further in the review process.

Overall comments:

a) Given the paper's interpretive qualitative approach, the authors may consider avoiding making claims and interpreting data from a positivist stance, i.e. that results and conclusions should be generalizable, in order to maintain some level of epistemological consistency. As it stands, the nature of the data (few participants, rich transcripts) appears to contradict the nature of these generalizing claims.

b) Please double-check check the paper for clarity and brevity.

1. Introduction:

a) It would be helpful if the authors briefly described the types of labor typically employed on platform-based gig work sites like Fiverr and Upwork.

b) The phrasing of the study's research questions are written in such a way that a quantitative research design would more aptly answer them. Consider re-phrasing them to be more open-ended and centered on capturing themes of lived human experience, rather than an investigation of the relationships between operationalized variables.

3.1 Population and sample:

a) The sampling of acquaintances is a major methodological concern that is not sufficiently addressed. The explanation offered ("Despite the population being known, the absence of the count of digital gig workers in Sri Lanka led to the application of a non-probability approach enabling the researcher to offer credible and meaningful results [44]") does not meaningfully justify this methodological decision; moreover, the citation offered does not make any supporting claims of this methodological decision as it restricts its argument to quantitative research. The authors must discuss steps taken to ensure bias was accounted for in both the interview and the data analysis processes.

3.2 Data collection method:

a) Please share more details about who conducted the interviews. Was it just one of the researchers, multiple, or other people entirely?

b) Please describe which core open-ended questions were asked during the interviews.

3.3 Participants:

a) Participants are skewed almost exclusively male and emerging adult, with the jobs exclusively relying on STEM-based knowledge. This severely limits the scope of the themes the authors are developing from the data. Please acknowledge this, either in this section or in the limitations.

b) It is unclear if gig work is participants' sole job. If data is available describing participants' primary employment, consider reporting it.

3.4 Data analysis:

a) While NVivo is an appropriate tool for qualitative analysis, the authors must still expand on their a priori frameworks and epistemological assumptions going into the analysis. Since thematic analysis is a (mostly) ideologically independent analytic framework, it is thus important for the authors to note their own lived experiences, personal investments, or ideological presuppositions with regard to the topic of research and the data at hand via some researcher reflexivity or positionally subsection, since these elements are unavoidably omnipresent throughout the qualitative research process. Moreover, the authors must make more clear the steps taken in analyzing the data for themes by ensuring each step is included and describing what each step entailed. The authors must also report on if one researcher analyzed the data, or if multiple researchers collaborated to develop themes (in which case, a statistical test for inter-rater reliability would be necessary).

4 Results:

a) Overall, please ensure that all claims remain within the scope of what your data can explain. Some interpretations of the data go beyond what the excerpts provided seem to suggest.

b) Please ensure all sections are where they belong. It looks like 4.2 got moved below 4.4.

5.1 Mapping intrinsic and extrinsic drivers in a Motivation Interplay Matrix:

a) While there may be a strong theoretical basis for this model based on previous literature, the excerpts provided do not sufficiently illustrate such a model. It also does not seem appropriate to use data that is not necessarily generalizable as the basis for a generalizable theoretical model. As it stands, this matrix may be more usefully deemed beyond the scope of the present paper, and may be more strong as a standalone piece.

7. Conclusion and limitations:

a) Based on the feedback provided, please ensure the study's limitations are expanded to include theoretical and methodological concerns.

Reviewer #3: This article is interesting and has good potential.

Kindly engage with deeper SDT Concepts like ' controlled verses autonomous motivation.'

And try to position your study against existing frameworks in literature.

The literature review should be synthesised instead of just summaries. Compare debates, identify problems that have not been resolved.

You also need to justify why your sampling technique is best for this study, why 14 interviews was enough. Give us enough details that will make the study replicable.

Also engage a proofreader for grammar and other minor issues.

Your Data availability conflicts with PLOS Requirements

**********

what does this mean?). If published, this will include your full peer review and any attached files.

If you choose “no”, your identity will remain anonymous but your review may still be made public.

**Do you want your identity to be public for this peer review?**  For information about this choice, including consent withdrawal, please see our Privacy Policy

Reviewer #1: No

Reviewer #2: No

Reviewer #3: **Yes:** John Adjah

**********

[NOTE: If reviewer comments were submitted as an attachment file, they will be attached to this email and accessible via the submission site. Please log into your account, locate the manuscript record, and check for the action link "View Attachments". If this link does not appear, there are no attachment files.]

To ensure your figures meet our technical requirements, please review our figure guidelines: https://journals.plos.org/plosone/s/figures

You may also use PLOS’s free figure tool, NAAS, to help you prepare publication quality figures: https://journals.plos.org/plosone/s/figures#loc-tools-for-figure-preparation.

NAAS will assess whether your figures meet our technical requirements by comparing each figure against our figure specifications.

Attachment Submitted filename: Reviewer_Report_PONE-D-26-12283.docx

# Author comment: Author response to Decision Letter 1

**DOI:** 10.1371/journal.pone.0345881.r002

6 Jul 2026

Please refer to the Response of the reviewer document.

Attachment Submitted filename: Response to reviewers- final.docx

# Peer review: Decision Letter 1

**DOI:** 10.1371/journal.pone.0345881.r003

**Authors:** Amruta Deshpande

1 Sep 2026

What Keeps Gig Workers Motivated? A Typology of the Intrinsic-Extrinsic Motivation Interplay in the Digital Gig Economy

PONE-D-26-12283R1

Dear Dr. Wisenthige,

We’re pleased to inform you that your manuscript has been judged scientifically suitable for publication and will be formally accepted for publication once it meets all outstanding technical requirements.

Within one week, you’ll receive an e-mail detailing the required amendments. When these have been addressed, you’ll receive a formal acceptance letter and your manuscript will be scheduled for publication.

An invoice will be generated when your article is formally accepted. Please note, if your institution has a publishing partnership with PLOS and your article meets the relevant criteria, all or part of your publication costs will be covered. Please make sure your user information is up-to-date by logging into Editorial Manager at Editorial Manager® and clicking the ‘Update My Information' link at the top of the page. For questions related to billing, please contact billing support.

If your institution or institutions have a press office, please notify them about your upcoming paper to help maximize its impact. If they’ll be preparing press materials, please inform our press team as soon as possible -- no later than 48 hours after receiving the formal acceptance. Your manuscript will remain under strict press embargo until 2 pm Eastern Time on the date of publication. For more information, please contact onepress@plos.org.

Kind regards,

Amruta Deshpande

Academic Editor

PLOS One

Additional Editor Comments (optional):

ACcept

Reviewers' comments:

Reviewer's Responses to Questions

**Comments to the Author**

Reviewer #4: All comments have been addressed

**********

2. Is the manuscript technically sound, and do the data support the conclusions?

Reviewer #4: Yes

**********

3. Has the statistical analysis been performed appropriately and rigorously?

Reviewer #4: Yes

**********

4. Have the authors made all data underlying the findings in their manuscript fully available?

The PLOS Data policy

Reviewer #4: Yes

**********

5. Is the manuscript presented in an intelligible fashion and written in standard English?

Reviewer #4: Yes

**********

Reviewer #4: The authors have addressed the comments fully all the way through. I recommend the publication of the article in its current form.

**********

what does this mean?). If published, this will include your full peer review and any attached files.

If you choose “no”, your identity will remain anonymous but your review may still be made public.

**Do you want your identity to be public for this peer review?**  For information about this choice, including consent withdrawal, please see our Privacy Policy

Reviewer #4: No

**********

# Editor report: Acceptance letter

**DOI:** 10.1371/journal.pone.0345881.r004

**Authors:** Amruta Deshpande

PONE-D-26-12283R1

PLOS One

Dear Dr. Wisenthige,

I'm pleased to inform you that your manuscript has been deemed suitable for publication in PLOS One. Congratulations! Your manuscript is now being handed over to our production team.

At this stage, our production department will prepare your paper for publication. This includes ensuring the following:

* All references, tables, and figures are properly cited

* All relevant supporting information is included in the manuscript submission,

* There are no issues that prevent the paper from being properly typeset

You will receive further instructions from the production team, including instructions on how to review your proof when it is ready. Please keep in mind that we are working through a large volume of accepted articles, so please give us a few days to review your paper and let you know the next and final steps.

Lastly, if your institution or institutions have a press office, please let them know about your upcoming paper now to help maximize its impact. If they'll be preparing press materials, please inform our press team within the next 48 hours. Your manuscript will remain under strict press embargo until 2 pm Eastern Time on the date of publication. For more information, please contact onepress@plos.org.

You will receive an invoice from PLOS for your publication fee after your manuscript has reached the completed accept phase. If you receive an email requesting payment before acceptance or for any other service, this may be a phishing scheme. Learn how to identify phishing emails and protect your accounts at https://explore.plos.org/phishing.

If we can help with anything else, please email us at customercare@plos.org.

Thank you for submitting your work to PLOS One and supporting open access.

Kind regards,

PLOS One Editorial Office Staff

on behalf of

Dr. Amruta Deshpande

Academic Editor

PLOS One
